# Activated ATF6α is a hepatic tumour driver restricting immunosurveillance

**DOI:** 10.1038/s41586-025-10036-8

**Published:** 2026-02-04

**Authors:** Xin Li, Cynthia Lebeaupin, Aikaterini Kadianaki, Clementine Druelle-Cedano, Niklas Vesper, Charlotte Rennert, Júlia Huguet-Pradell, Borja Gomez Ramos, Chaofan Fan, Robert Stefan Piecyk, Laimdota Zizmare, Pierluigi Ramadori, Luqing Li, Lukas Frick, Menjie Qiu, Cangang Zhang, Luiza Martins Nascentes Melo, Vikas Prakash Ranvir, Peng Shen, Johannes Hanselmann, Jan Kosla, Mirian Fernández-Vaquero, Mihael Vucur, Praveen Baskaran, Xuanwen Bao, Olivia I. Coleman, Yingyue Tang, Miray Cetin, Zhouji Chen, Insook Jang, Stefania Del Prete, Mohammad Rahbari, Peng Zhang, Timothy V. Pham, Yushan Hou, Aihua Sun, Li Gu, Laura C. Kim, Ulrike Rothermel, Danijela Heide, Adnan Ali, Suchira Gallage, Nana Talvard-Balland, Marta Piqué-Gili, Albert Gris-Oliver, Alessio Bevilacqua, Lisa Schlicker, Alec Duffey, Kristian Unger, Marta Szydlowska, Jenny Hetzer, Duncan T. Odom, Tim Machauer, Daniele Bucci, Pooja Sant, Jun-Hoe Lee, Jonas Rösler, Sven W. Meckelmann, Johannes Schreck, Sue Murray, M. Celeste Simon, Sven Nahnsen, Almut Schulze, Ping-Chih Ho, Manfred Jugold, Kai Breuhahn, Jan-Philipp Mallm, Peter Schirmacher, Susanne Roth, Nuh Rahbari, Darjus F. Tschaharganeh, Stephanie Roessler, Benjamin Goeppert, Bertram Bengsch, Geoffroy Andrieux, Melanie Boerries, Nisar P. Malek, Marco Prinz, Achim Weber, Robert Zeiser, Pablo Tamayo, Peter Bronsert, Konrad Kurowski, Robert Thimme, Detian Yuan, Rafael Carretero, Tom Luedde, Roser Pinyol, Felix J. Hartmann, Michael Karin, Alpaslan Tasdogan, Christoph Trautwein, Moritz Mall, Maike Hofmann, Josep M. Llovet, Dirk Haller, Randal J. Kaufman, Mathias Heikenwälder

**Affiliations:** 1 Division of Chronic Inflammation and Cancer, German Cancer Research Center (DKFZ), Heidelberg, Germany; 2 Faculty of Biosciences, Heidelberg University, Heidelberg, Germany; 3 Center for Metabolic and Liver Disease, Sanford Burnham Prebys Medical Discovery Institute, La Jolla, CA USA; 4 Department of Medicine II, University Hospital Freiburg, Faculty of Medicine, University of Freiburg, Freiburg, Germany; 5 Faculty of Biology, University of Freiburg, Freiburg, Germany; 6 Liver Cancer Translational Research Group, Liver Unit, Institut d’Investigacions Biomèdiques August Pi i Sunyer (IDIBAPS)-Hospital Clínic, Universitat de Barcelona, Barcelona, Spain; 7 Mount Sinai Liver Cancer Program, Divisions of Liver Diseases, Department of Hematology/Oncology, Department of Medicine, Tisch Cancer Institute, Icahn School of Medicine at Mount Sinai, New York, NY USA; 8 Division of Cell Fate Engineering and Disease Modeling, German Cancer Research Center (DKFZ) and DKFZ-ZMBH Alliance, Heidelberg, Germany; 9 HITBR Hector Institute for Translational Brain Research, Heidelberg, Germany; 10 Central Institute of Mental Health, Medical Faculty Mannheim, Heidelberg University, Mannheim, Germany; 11 Department of Radiation Oncology, University Hospital, LMU Munich, Munich, Germany; 12 Bavarian Cancer Research Center (BZKF), Munich, Germany; 13 Werner Siemens Imaging Center (WSIC), Department of Preclinical Imaging and Radiopharmacy, Eberhard Karls University Tübingen, Tübingen, Germany; 14 Cluster of Excellence iFIT (EXC 2180) ‘Image-Guided and Functionally Instructed Tumor Therapies’, University of Tübingen, Tübingen, Germany; 15 M3-Research Center for Malignome, Metabolome and Microbiome, Institute for Interdisciplinary Research on Cancer Metabolism and Chronic Inflammation, Faculty of Medicine, University of Tübingen, Tübingen, Germany; 16 Institute of Neuropathology, University Hospital Zurich, University of Zurich, Zurich, Switzerland; 17 Department of Pathogenic Microbiology and Immunology, School of Basic Medical Sciences, Xi’an Jiaotong University, Xi’an, China; 18 Department of Dermatology, University Hospital Essen, Essen, Germany; 19 German Cancer Consortium (DKTK) Partner Site, Essen, Germany; 20 Department of Gastroenterology, Hepatology and Infectious Diseases, University Hospital Düsseldorf, Medical Faculty at Heinrich-Heine-University, Düsseldorf, Germany; 21 Quantitative Biology Center (QBiC), Eberhard Karls University Tübingen, Tübingen, Germany; 22 Department of Medical Oncology, The First Affiliated Hospital, Zhejiang University School of Medicine, Hangzhou, China; 23 Chair of Nutrition and Immunology, Technische Universität München, Freising, Germany; 24 Institute of Pathology, University Hospital Heidelberg, Heidelberg, Germany; 25 Systems Immunology and Single-Cell Biology, German Cancer Research Center (DKFZ), Heidelberg, Germany; 26 Division of Regulatory Genomics and Cancer Evolution, German Cancer Research Center (DKFZ), Heidelberg, Germany; 27 Department of Biochemistry and Molecular Biology, School of Basic Medical Sciences, Cheeloo College of Medicine, Shandong University, Jinan, China; 28 Center for Novel Therapeutics and Moores Cancer Center, University of California San Diego, La Jolla, CA USA; 29 State Key Laboratory of Proteomics, Beijing Proteome Research Center, National Center for Protein Sciences (Beijing), Institute of Lifeomics, Beijing, China; 30 Laboratory of Gene Regulation and Signal Transduction, Departments of Pharmacology and Pathology, School of Medicine, University of California San Diego, La Jolla, CA USA; 31 Department of Laboratory Medicine, West China Hospital, Sichuan University, Chengdu, China; 32 Clinical Laboratory Medicine Research Center, West China Hospital, Sichuan University, Chengdu, China; 33 Abramson Family Cancer Research Institute, Perelman School of Medicine, University of Pennsylvania, Philadelphia, PA USA; 34 Department of Cell and Developmental Biology, University of Pennsylvania, Philadelphia, PA USA; 35 Department of Hematology and Oncology, Freiburg University Medical Centre, Freiburg, Germany; 36 Department of Oncology, University of Lausanne, Epalinges, Switzerland; 37 Division of Tumor Metabolism and Microenvironment, German Cancer Research Center (DKFZ), Heidelberg, Germany; 38 German Cancer Consortium (DKTK), Partner Site Munich, German Cancer Research Center (DKFZ), Heidelberg, Germany; 39 Comprehensive Cancer Center Munich, LMU University Hospital, Munich, Germany; 40 Research Unit Translational Metabolic Oncology (TMO), Institute for Diabetes and Cancer (IDC), Helmholtz Diabetes Center, Helmholtz Munich, Neuherberg, Germany; 41 German Center for Diabetes Research (DZD), Neuherberg, Germany; 42 Single-Cell Open Lab, German Cancer Research Center (DKFZ), Heidelberg, Germany; 43 Applied Analytical Chemistry, University of Duisburg-Essen, Essen, Germany; 44 Ionis Pharmaceuticals, Carlsbad, CA USA; 45 Biomedical Data Science, Department of Computer Science, University of Tübingen, Tübingen, Germany; 46 Ludwig institute for Cancer Research, University of Lausanne, Epalinges, Switzerland; 47 Core Facility Small Animal Imaging, German Cancer Research Center Heidelberg, Heidelberg, Germany; 48 Department of General, Visceral and Transplantation Surgery, University of Heidelberg, Heidelberg, Germany; 49 Department of General, Visceral and Transplantation Surgery, Tübingen University Hospital, Tübingen, Germany; 50 Institute of Pathology, RKH Hospital, Ludwigsburg, Germany; 51 Institute of Tissue Medicine and Pathology, University of Berne, Berne, Switzerland; 52 Signalling Research Centres BIOSS and CIBSS, University of Freiburg, Freiburg, Germany; 53 Institute of Medical Bioinformatics and Systems Medicine, Medical Center-University of Freiburg, Faculty of Medicine, University of Freiburg, Freiburg, Germany; 54 German Cancer Consortium (DKTK), Partner site Freiburg, a partnership between DKFZ and Medical Center-University of Freiburg, Freiburg, Germany; 55 Department Internal Medicine I, University Hospital Tübingen, Tübingen, Germany; 56 Institute of Neuropathology, Medical Faculty, University of Freiburg, Freiburg, Germany; 57 CIBSS Centre for Integrative Biological Signalling Studies, University of Freiburg, Freiburg, Germany; 58 Center for Brain Research and Advancements in Neuroimmunology (BRAIN), Faculty of Medicine, University of Freiburg, Freiburg, Germany; 59 Department of Pathology and Molecular Pathology, University Hospital of Zurich, Zurich, Switzerland; 60 Institute of Molecular Cancer Research, University of Zurich, Zurich, Switzerland; 61 Division of Genomics and Precision Medicine, Department of Medicine, University of California San Diego, La Jolla, CA USA; 62 Core Facility for Histopathology and Digital Pathology, University Hospital Freiburg, Faculty of Medicine, University of Freiburg, Freiburg, Germany; 63 Institute for Surgical Pathology, Medical Center, Faculty of Medicine, University of Freiburg, Freiburg, Germany; 64 DKFZ-Bayer Immunotherapeutic Lab, German Cancer Research Center (DKFZ), Heidelberg, Germany; 65 Core Facility Metabolomics, Medical Faculty University of Tübingen, Tübingen, Germany; 66 Institució Catalana de Recerca i Estudis Avançats (ICREA), Barcelona, Spain; 67 ZIEL Institute for Food and Health, Technische Universität München, Freising, Germany; 68 Cluster of Excellence EXC 2124 Controlling Microbes to Fight Infections, University of Tübingen, Tübingen, Germany

**Keywords:** Liver cancer, Cancer metabolism, Cancer therapy

## Abstract

Hepatocellular carcinoma (HCC) is the fastest growing cause of cancer-related mortality and there are limited therapies^1^. Although endoplasmic reticulum (ER) stress and the unfolded protein response (UPR) are implicated in HCC, the involvement of the UPR transducer ATF6α remains unclear^2^. Here we demonstrate the function of ATF6α as an ER-stress-inducing tumour driver and metabolic master regulator restricting cancer immunosurveillance for HCC, in contrast to its well-characterized role as an adaptive response to ER stress^3^. ATF6α activation in human HCC is significantly correlated with an aggressive tumour phenotype, characterized by reduced patient survival, enhanced tumour progression and local immunosuppression. Hepatocyte-specific ATF6α activation in mice induced progressive hepatitis with ER stress, immunosuppression and hepatocyte proliferation. Concomitantly, activated ATF6α increased glycolysis and directly repressed the gluconeogenic enzyme FBP1 by binding to gene regulatory elements. Restoring FBP1 expression limited ATF6α-activation-related pathologies. Prolonged ATF6α activation in hepatocytes triggered hepatocarcinogenesis, intratumoural T cell infiltration and nutrient-deprived immune exhaustion. Immune checkpoint blockade (ICB)^4^ restored immunosurveillance and reduced HCC. Consistently, patients with HCC who achieved a complete response to immunotherapy displayed significantly increased ATF6α activation compared with those with a weaker response. Targeting *Atf6* through germline ablation, hepatocyte-specific ablation or therapeutic hepatocyte delivery of antisense oligonucleotides dampened HCC in preclinical liver cancer models. Thus, prolonged ATF6α activation drives ER stress, leading to glycolysis-dependent immunosuppression in liver cancer and sensitizing to ICB. Our findings suggest that persistently activated ATF6α is a tumour driver, a potential stratification marker for ICB response and a therapeutic target for HCC.

## Abstract (web summary)

ATF6α activation in human and preclinical models of hepatocellular carcinoma is significantly associated with an aggressive tumour phenotype characterized by reduced survival, glycolytic reprogramming and local immunosuppression.

## Main

HCC accounts for 80–85% of primary liver cancers^1^ and derives mostly from malignant, transformed hepatocytes in chronic hepatitis^5,6^. Despite advances in immunotherapies that improved survival, the complex genetic, metabolic and inflammatory interactions remain a barrier towards effective treatment. HCC-infiltrating lymphocytes express exhaustion markers (such as PD-1, CTLA-4) leading to poor prognosis^7,8^. Improving T-cell-mediated tumour surveillance with ICB (atezolizumab) and VEGF blockade (bevacizumab) is the standard of care for unresectable HCC^4,9–11^. Metabolic reprogramming, including glucose deprivation in HCC and intratumoural hypoxia, was proposed to reduce anti-tumour therapy efficacy and enhance malignancy^12^. Thus, new strategies are needed to overcome metabolism-related tumour escape and immunosuppression.

ER stress and UPR activation represent negative prognostic factors in cancer^2,13^ and are implicated in liver diseases^14^. The UPR encompasses three ER transmembrane proteins with downstream signalling cascades: (1) PKR-like ER kinase (PERK); (2) inositol-requiring enzyme 1 alpha (IRE1α); and (3) activating transcription factor 6 alpha (ATF6α), which is cleaved to generate an N-terminal p50 fragment (nATF6α) that enters the nucleus to activate ER chaperones and lipid synthesis genes^14,15^. While PERK and IRE1α may promote cancer hallmarks^2^, less is known about ATF6α, of which the chronic activation in intestinal epithelial cells induces microbiota-dependent colon adenomas^15^. Here, in contrast to its characterized adaptive role in acute ER stress^3,16^, we describe ATF6α as a tumour driver and master regulator of glucose metabolism contributing to immunosuppression, with implications for HCC therapy.

## ATF6α activation marks aggressive HCC

**Fig. 1:**
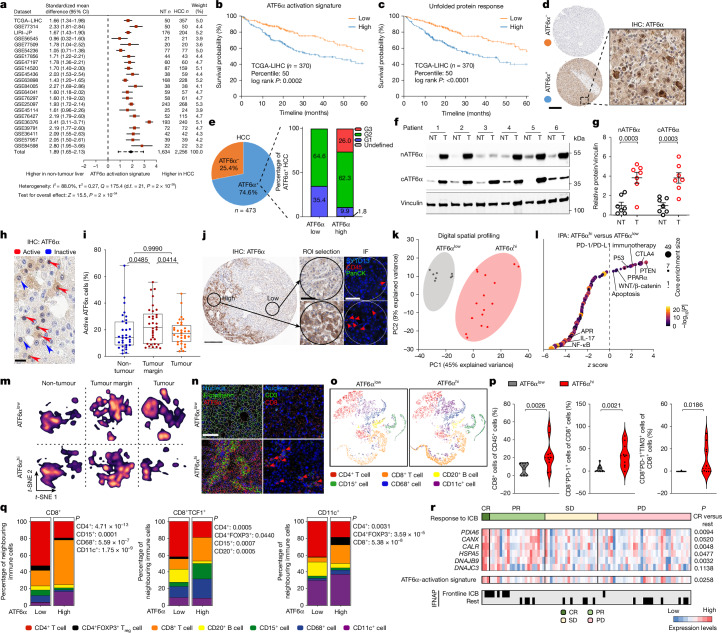
Hepatic ATF6α activation is prognostic in human HCC. **a**, Forest plot of the human ATF6α*-*activation signature in HCC versus non-tumour liver in 22 datasets. CI, confidence interval. **b**,**c**, Kaplan–Meier survival curves for TCGA-LIHC patients split by median enrichment of the human ATF6α-activation signature (**b**) or the Reactome_Unfolded_Protein_Response_UPR (**c**) MSigDB^17^ human gene set. **d**,**e**, Representative ATF6α IHC identifying ATF6α^−^ or ATF6α^+^ samples in human HCC TMAs (**d**) and ATF6α^+^ tumour grading (**e**; G1–G3)^20^. Scale bar, 200 μm (**d**). **f**,**g**, Representative immunoblot (**f**) and quantification (**g**) of ATF6α in non-tumour (NT) and tumour (T) lysates from patients with HCC. Vinculin was used as the loading control, run on a cATF6α blot. **h**, IHC analysis of active (nuclear) and inactive ATF6α in human HCC. Scale bar, 20 µm. **i**, The percentage of active ATF6α cells in non-tumour liver, tumour margin or tumour centre from patients with HCC. **j**, Human HCC TMAs with low or high ATF6α expression as determined using IHC (left) were selected for spatial transcriptomics from annotated 300-µm-diameter circular ROIs (right). Scale bars, 400 μm (left) and 100 μm (right). IF, immunofluorescence. **k**, Principal component analysis (PCA) of spatial transcriptomics with low versus high ATF6α ROIs. **l**, Ingenuity pathway analysis (IPA) of differentially expressed genes in high versus low ATF6α ROIs. **m**, *t*-Distributed stochastic neighbour embedding (*t*-SNE) plot of liver cell types by IMC in ATF6α^low^ versus ATF6α^hi^ livers from patients with HCC. **n**, Representative IMC of ATF6α^low^ and ATF6α^hi^ HCC. ATF6α (red), E-cadherin (green) and nuclei (blue, left), and CD3^+^ T cells (green), CD8^+^ T cells (red) and nuclei (blue, right) are shown. Scale bar, 60 μm. **o**,**p**, *t*-SNE plots of the immune cell distribution (**o**) and CD8^+^ T cell subset composition (**p**) in ATF6α^low^ and ATF6α^hi^ HCC, as determined using IMC. **q**, Immune neighbourhood analysis of CD8^+^, CD8^+^TCF1^+^ and CD11c^+^ cells in ATF6α^low^ and ATF6α^hi^ HCC. **r**, ATF6α target gene expression and the human ATF6α-activation signature sorted by response to anti-PD-1 monotherapy^25^. CR, complete response; PD, progressive disease; PR, partial response; SD, stable disease. Sample sizes, biological replicates and statistical tests are described in the Methods and Source data. Source data

**Extended Data Fig. 1:**
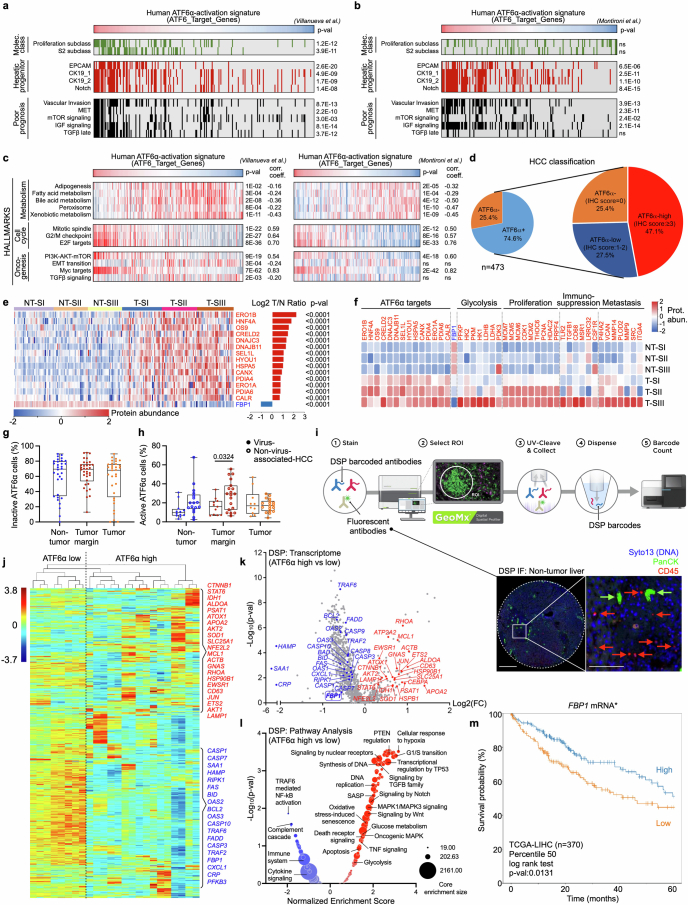
High levels of ATF6α activation are associated with aggressive HCC subtypes and *Fbp1* downregulation in humans, Related to Fig. 1. (**a-b**) Heatmap of HCC molecular subtype, hepatic progenitor and poor prognosis gene signatures in 228 HCC tumours^18^ (**a**) and 171 HCC tumours^19^ (**b**) distributed by high to low enrichment of human ATF6α-activation signature (MSigDB^17^ Human Gene Set: ATF6_Target_Genes^75^). (**c**) Heatmap of metabolism, cell cycle, or oncogenesis MSigDB Hallmark gene sets of tumour samples^18,19^ in 228 HCC tumours^18^ (left) and 171 HCC tumours^19^ (right) distributed by high to low enrichment of human ATF6α-activation signature. (**d**) Tumour grade distribution of ATF6α-negative and ATF6α-positive (high and low) HCC samples from human HCC tissue microarrays (TMAs) (n = 473; ATF6α-: n = 120 and ATF6α+ : n = 353 patients (low: n = 130; high: n = 223)). Compared to ATF6α-negative samples (IHC score=0, 25.4%), the remaining 74.6% ATF6α-positive samples were binned into distinct groups based on ATF6α activation: (i) low (IHC score 1-2; 27.5% of total) or (ii) high (IHC score ≥3; 47.1% of total). (**e-f**) Heatmap of nATF6α target protein expression (**e**) regrouped by signalling pathway (**f**) in non-tumour liver (NT) or paired tumour (T) tissue from the Chinese Human Proteome Project HCC patients of increasingly aggressive S-I, S-II, and S-III subtypes^21^ (n = 101: S-I, n = 36; S-II, n = 32; S-III, n = 33). (**g**) Percentage of cells with inactive (cytoplasmic) ATF6α expression in non-tumour tissue (n = 32), tumour margin (n = 34) or tumour (n = 32) based on IHC of ATF6α in HCC patients. (**h**) Percentage of cells with active (nuclear) ATF6α expression distributed by viral (n = 12 non-tumour, 11 tumour margin, 10 tumour) or non-viral (n = 17 non-tumour, 19 tumour margin, 18 tumour) hepatitis HCC patients. (**i**) DSP workflow, adapted from user’s manual (image provided by Bruker Spatial Biology). Representative DSP immunofluorescence staining in liver tissue of HCC patient as technical control (below). Red arrows indicate CD45+ (immune) cells and green arrows indicate PanCK+ (biliary) cells. (**j**) Heatmap of unsupervised hierarchical clustering of nearly 1200 genes from the Cancer Transcriptome Atlas panel revealing distinct high-ATF6α versus low-ATF6α HCC ROIs. (**k**) Volcano plot of significantly downregulated (blue) and upregulated (red) transcripts in high-ATF6α versus low-ATF6α ROIs. (**l**) DSP Pathway Analysis of differentially expressed genes in high-ATF6α versus low-ATF6α HCC ROIs. (**m**) Kaplan-Meier survival curves for TCGA-LIHC patients divided into two groups by median split based on high (n = 185) or low *FBP1* (n = 185) mRNA expression. Scatter dot plot data are presented as mean values ± SEM. Box and whisker plot data are presented with boxes spanning 25th to 75th percentiles, with a line at the median, and whiskers from min to max. Data in 1a-c with continuous variables were analysed by Pearson correlation, while categorical variables were compared by Wilcoxon test. Data in 1e were mined and re-processed for analysis by Wilcoxon rank sum test. Data in 1g-h were analysed by one-way ANOVA with Geisser-Greenhouse correction, Tukey multiple comparison correction. D’Agostino-Pearson, Shapiro-Wilk, and Kolmogorov-Smirnov tests were performed to assess for normal distribution. Lognormally distributed cohorts were log-transformed prior to testing. Data in 1m were analysed by median split and log-rank test. Source data

Patient-derived liver sections showed nuclear ATF6α expression (Supplementary Fig. 1a–d), suggesting ER stress and ATF6α activation in chronic hepatitis. In 22 distinct human HCC datasets, *ATF6* mRNA (encoding ATF6α) and the human ATF6α-activation signature derived from the Molecular Signatures Database (MSigDB)^17^ were consistently and significantly increased in HCC compared with non-tumour livers (Fig. 1a and Supplementary Fig. 1e). The human ATF6α-activation signature and UPR signature from MSigDB^17^ were significantly associated with reduced patient survival in The Cancer Genome Atlas Liver Hepatocellular Carcinoma (TCGA-LIHC) dataset (Fig. 1b,c). ATF6α activation appeared to be the primary UPR driver and was uniquely associated with HCC compared with various cancers; other canonical UPR components did not correlate with survival probability in TCGA-LIHC and the human ATF6α-activation signature was not associated with reduced survival in other TCGA cancer datasets (Fig. 1b,c and Supplementary Fig. 1f,g). Patient-derived HCC samples^18,19^ stratified by the human ATF6α-activation signature were enriched in hepatic progenitor transformation, poor prognosis, cell cycle, oncogenesis hallmarks and lower scores of metabolism-related signatures (Extended Data Fig. 1a–c).

Immunohistochemical scoring of human HCC tissue microarray (TMA) revealed that, among the 74.6% ATF6α-positive samples, those with high versus low ATF6α activation were associated with high-grade (G3) HCC^20^ (Fig. 1d,e and Extended Data Fig. 1d). Evaluation of the Chinese Human Proteome Project^21^ corroborated that tumour samples of subgroup SIII of patients with HCC, characterized by low overall survival and poor prognosis after first-line surgery, exhibited increased expression of ATF6α targets and glycolysis-related proteins, coupled with significantly reduced FBP1 expression, and enrichment of proteins linked to proliferation, immunosuppression and metastasis (Extended Data Fig. 1e,f). Immunoblotting of protein lysates from patients with HCC showed preferential accumulation of cleaved ATF6α (cATF6α, C terminal, inactive; nATF6α, N terminal, active) in tumours (Fig. 1f,g). Whereas nATF6α localized to hepatocyte nuclei at the tumour margin of patients with non-virus-associated HCC, inactive, cytoplasmic ATF6α remained unchanged (Fig. 1h,i and Extended Data Fig. 1g,h).

## ATF6α activation is a tumour driver

Using spatial transcriptomics, we sequenced regions of interest (ROIs) based on immunohistochemistry (IHC)-stained human HCC serial sections for high and low ATF6α expression (Fig. 1j–l and Extended Data Fig. 1i–l). The ATF6α-specific chaperone *HSP90B1* and cancer-related genes were significantly increased in transcriptionally distinct ATF6α^hi^ versus ATF6α^low^ regions (Fig. 1k and Extended Data Fig. 1j,k). High ATF6α expression was associated with PD-1–PD-L1, CTLA-4 signalling, hypoxia, cell cycle progression and glycolysis, the latter of which was linked to downregulated *FBP1*; the low expression of *FBP1* was correlated with an aggressive HCC subtype (T-SIII; Extended Data Fig. 1e,f) and reduced survival of patients with HCC (Fig. 1l and Extended Data Fig. 1k–m). Reduced FBP1 in stressed hepatocytes and HCC progenitor cells acts as a metabolic switch: reversing senescence, supporting proliferation and promoting DNA-damage-induced mutations in MASH-HCC^22^.

Imaging mass cytometry (IMC) demonstrated altered site-specific (that is, non-tumour, tumour margin, tumour) cellular composition, including parenchymal, stromal and immune cells, particularly within samples from patients with HCC exhibiting high ATF6α activation (Fig. 1m and Supplementary Fig. 2). ATF6α^hi^ versus ATF6α^low^ tumour regions presented increased CD8^+^ T cell infiltration and other immune cell abundance (for example, CD11c^+^ cells), where the percentage of CD8^+^PD-1^+^ T cells and CD8^+^PD-1^+^TIM3^+^ terminally exhausted T cells was significantly higher (Fig. 1n–p and Supplementary Fig. 2f). Neighbourhood analysis of CD8^+^ T cells revealed expanded CD8^+^ T cells clustering within a hub comprising CD11c^+^ dendritic cells (DCs) and CD4^+^ T cells in ATF6α^hi^ tumours, reminiscent of recently reported hepatic niches locally promoting CD8^+^ T cell responses^23,24^. Preferential colocalization of CD11c^+^ DCs with CD8^+^ T cells and FOXP3^+^CD4^+^ regulatory T (T_reg_) cells in ATF6α^hi^ HCC revealed increased immunosuppression, while the ICB-responsive CD8^+^TCF1^+^ T cell subset hardly colocalized with T_reg_ cells in ATF6α^hi^ HCC (Fig. 1q). As this may reveal mechanisms underlying sensitization of ATF6α^hi^ tumours to ICB, we investigated 83 samples from patients with advanced HCC receiving anti-PD-1 monotherapy^25^ and found that those with a complete response had significantly higher expression of ATF6α target genes and the human ATF6α-activation signature (Fig. 1r).

## Hepatic ATF6α activation induces injury

**Fig. 2:**
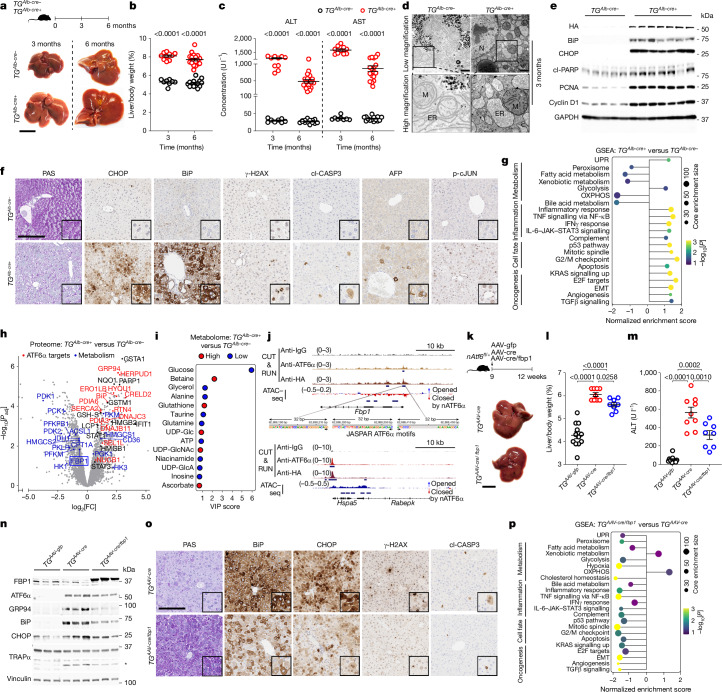
Persistent ATF6α activation in hepatocytes induces liver injury and glucose metabolic dysfunction through FBP1 repression. **a**, Schematic and liver images of *TG*^*Alb-cre−*^ and *TG*^*Alb-cre+*^ mice euthanized aged 3 or 6 months. Scale bar, 1 cm. **b**,**c**, The liver-to-body weight (**b**) and serum ALT and AST levels (**c**) of 3- and 6-month-old *TG*^*Alb-cre−*^ and *TG*^*Alb-cre+*^ mice. **d**, Representative liver TEM images of *TG*^*Alb-cre−*^ and *TG*^*Alb-cre+*^ mice showing ER, mitochondria (M) and nucleus (N). Scale bar, 1 µm. Quantification is shown in Extended Data Fig. 2g. **e**, Representative immunoblot of liver lysates from 3-month-old *TG*^*Alb-cre−*^ and *TG*^*Alb-cre+*^ mice. GAPDH was used as the loading control, run on a BiP blot. Quantification is shown in Extended Data Fig. 2i. **f**, Representative periodic acid–Schiff (PAS) staining and IHC for the indicated proteins in the livers of 3-month-old *TG*^*Alb-cre−*^ and *TG*^*Alb-cre+*^ mice. Scale bar, 200 µm. Quantification is shown in Extended Data Fig. 2j. **g**–**i**, GSEA^27^ of liver RNA-seq (**g**), volcano plot of liver proteomic analysis (**h**) and NMR-based liver metabolic analysis (**i**) of 3-month-old *TG*^*Alb-cre+*^ versus *TG*^*Alb-cre−*^ mice.  EMT, epithelial-mesenchymal transition; FC, fold change; VIP score, variable importance score. **j**, CUT&RUN and ATAC–seq analysis of livers from 3-month-old *TG*^*Alb-cre+*^ versus *TG*^*Alb-cre−*^ mice. Anti-IgG (control), anti-ATF6α (endogenous ATF6α) or anti-HA (exogenous nATF6α-HA) antibodies are indicated for CUT&RUN, while three JASPAR motifs aligning with the *Fbp1* promoter predicted to bind to ATF6α motifs are shown. *Hspa5* was used as the positive control. *Rabepk* is the neighbouring gene. **k**, Schematic of AAV8-gfp-, AAV8-cre- or AAV8-cre/fbp1-injected mice euthanized 3 weeks after injection, with liver images of *TG*^*AAV-cre*^ and *TG*^*AAV-cre/fbp1*^ mice. Scale bar, 1 cm. **l**,**m**, The liver-to-body weight (**l**) and serum ALT levels (**m**) of *TG*^*AAV-gfp*^, *TG*^*AAV-cre*^ and *TG*^*AAV-cre/fbp1*^ mice. **n**, Representative immunoblot of liver lysates from *TG*^*AAV-gfp*^, *TG*^*AAV-cre*^ and *TG*^*AAV-cre/fbp1*^ mice. Vinculin was used as the loading control, run on a TRAPα blot. Quantification is shown in Extended Data Fig. 3d. **o**, Representative PAS staining and IHC for indicated proteins in *TG*^*AAV-cre*^ and *TG*^*AAV-cre/fbp1*^ mouse livers. Scale bar, 200 µm. Quantification is shown in Extended Data Fig. 3e. **p**, GSEA^27^ of liver RNA-seq data from *TG*^*AAV-cre/fbp1*^ versus *TG*^*AAV-cre*^ mice. Sample sizes, biological replicates and statistical tests are described in the Methods and Source data. Source data

**Extended Data Fig. 2:**
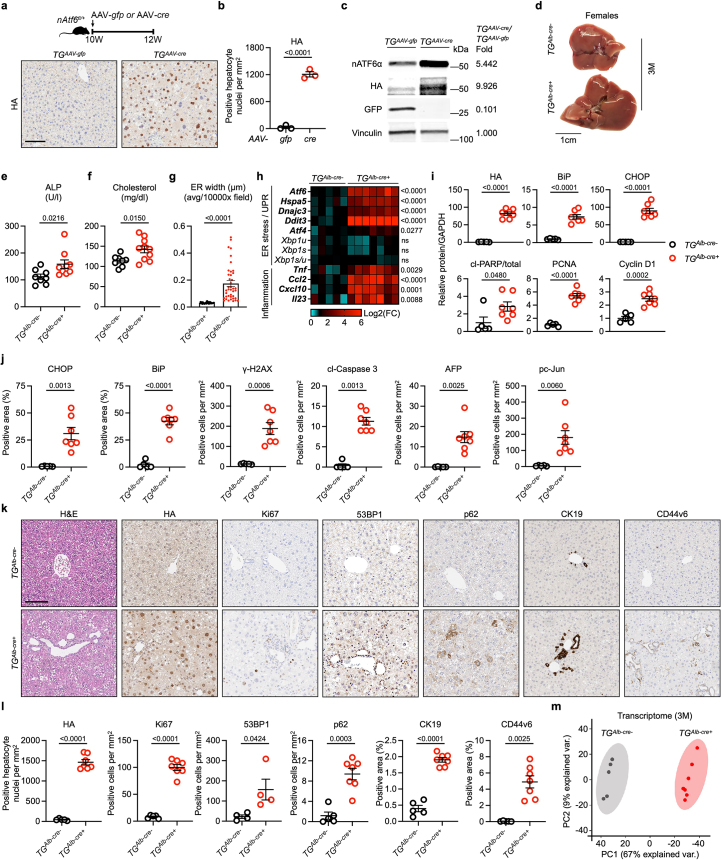
Persistent ATF6α activation causes ER stress-driven liver injury in 3-month-old mice, Related to Fig. 2. (**a-b**) Scheme (top) of *nAtf6*^*fll+*^ mice injected with AAV8-*gfp* (n = 3) or AAV8-*cre* (n = 3), the latter inducing heterozygous expression of nATF6-HA in hepatocytes, with representative liver IHC for HA (bottom, **a**; scale bar = 200 µm) and related quantification (**b**). (**c**) Representative immunoblot analysis to validate ATF6α and HA expression in livers of *TG*^*AAV-cre*^ mice, and GFP expression in livers of *TG*^*AAV-gfp*^ mice. VINCULIN as loading control, ran on GFP blot. kDa: kilodalton. (**d**) Representative liver macroscopy of female *TG*^*Alb-cre−*^ and *TG*^*Alb-cre+*^ mice sacrificed at 3 months of age. (**e-f**) Serum alkaline phosphatase (ALP, **e**) levels of *TG*^*Alb-cre−*^ (n = 8) and *TG*^*Alb-cre+*^ (n = 9) mice and cholesterol (**f**) levels of *TG*^*Alb-cre−*^ (n = 8) and *TG*^*Alb-cre+*^ (n = 10) mice. (**g**) ER width quantification of TEM images shown in Fig. 2d from livers of *TG*^*Alb-cre−*^ (n = 28 fields of view (FOV), 2 mice) and *TG*^*Alb-cre+*^ (n = 40 FOV, 4 mice). (**h**) qRT-PCR analysis of indicated mRNAs from livers of *TG*^*Alb-cre−*^ (n = 5) and *TG*^*Alb-cre+*^ (n = 7) mice. Bold labelling depicts significant genes. (**i**) Quantification of immunoblot analysis in Fig. 2e from livers of *TG*^*Alb-cre−*^ (n = 5) and *TG*^*Alb-cre+*^ (n = 7) mice. (**j**) Quantification of IHC in Fig. 2f from livers of *TG*^*Alb-cre−*^ (n = 5) and *TG*^*Alb-cre+*^ (n = 7) mice. (**k-l**) Representative H&E staining and IHC (**k**; scale bar = 100μm) with quantification (**l**) for HA, Ki67, 53BP1, p62, CK19, and CD44v6 from livers of 3-month-old *TG*^*Alb-cre−*^ (n = 4 for 53BP1, n = 5 for the rest) and *TG*^*Alb-cre+*^ (n = 4 for 53BP1, n = 7 for the rest) mice. (**m**) PCA of RNA-seq data from livers of 3-month-old *TG*^*Alb-cre-*^ (n = 5) and *TG*^*Alb-cre+*^ (n = 7) mice, showing unsupervised clustering of liver transcriptome. Scatter dot plot data are presented as mean values ± SEM. Data in 2b,e-j,l were analysed by two-tailed unpaired *t*-test or Mann-Whitney test based on data normality distribution. Mouse icon was created in BioRender. Heikenwälder, M. (2026) https://BioRender.com/lgjnsy9. Source data

To investigate the role of ATF6α activation in hepatocytes, we generated heterozygous transgenic mice with hepatocyte-specific Cre-inducible HA-tagged nATF6α (*TG*^*Alb-cre+*^, *TG*^*AAV-cre*^) and controls (*TG*^*Alb-cre−*^, *TG*^*AAV-gfp*^) (Extended Data Fig. 2a–c and Supplementary Table 1). *TG*^*Alb-cre+*^ mice at 3 and 6 months of age exhibited hepatomegaly and liver damage, characterized by increased liver-to-body weight and serum alanine–aspartate aminotransferases (ALT–AST), among others (Fig. 2a–c, Extended Data Fig. 2d–f and Supplementary Fig. 3a–d). Transmission electron microscopy (TEM) analysis demonstrated ER stress in 3-month-old *TG*^*Alb-cre+*^ livers by notable swelling and disruption of the stacked ER lamellar sheet structure (Fig. 2d and Extended Data Fig. 2g), corroborated by increased mRNA and protein expression of ATF6α and UPR targets (Fig. 2e–h and Extended Data Fig. 2h–j). Western blot and IHC analyses of ATF6α (or HA tag) confirmed successful expression and nuclear translocation of activated nATF6α (Fig. 2e, Extended Data Fig. 2a–c,i,k,l and Supplementary Fig. 3e,f), showing similar fold induction to nATF6α levels in patients with hepatitis (Supplementary Fig. 1a–d). Hepatocyte ATF6α activation increased liver cell proliferation (for example, Ki-67, PCNA, cyclin D1), DNA damage and cell death (for example, γ-H2AX, cleaved PARP (cl-PARP), cleaved caspase 3 (cl-CASP3)), HCC marker AFP, cancer stem cell marker CD44v6, oncogenic p62^26^ and tumour suppressor p53-binding protein 1 (53BP1) (Fig. 2e,f and Extended Data Fig. 2i–l). RNA-sequencing (RNA-seq) followed by gene set enrichment analysis (GSEA)^27^ revealed upregulated UPR, glycolysis, inflammation, cell fate (for example, cell cycle, division, death) and oncogenic signalling, but downregulated detoxification and respiration pathways (for example, oxidative phosphorylation), in transcriptionally distinct *TG*^*Alb-cre+*^ versus *TG*^*Alb-cre−*^ livers (Fig. 2g and Extended Data Fig. 2m). Paralleling with human HCC (Extended Data Fig. 1c), fatty acid metabolism was transcriptionally downregulated, whereas the expression of cholesterol biosynthesis and homeostasis proteins (for example, HMGCS1, CD36) was increased in the livers of *TG*^*Alb-cre+*^ versus *TG*^*Alb-cre−*^ mice (Fig. 2g,h). ATF6α activation increased expression of glycolysis-related enzymes (for example, PKM, PGK1) but suppressed rate-limiting gluconeogenic enzymes, including FBP1, causing hepatic glycogen and glucose depletion in *TG*^*Alb-cre+*^ mice compared with the control mice (Fig. 2f–i). At 6 months of age, glycolysis, immune cell infiltration and oncogenic pathways remained enriched in the livers of *TG*^*Alb-cre+*^ mice (Supplementary Fig. 3g,h).

Chromatin binding of nATF6α in livers from *TG*^*Alb-cre+*^ mice was determined by CUT&RUN using antibodies detecting endogenous ATF6α or exogenous HA-tagged nATF6α, therefore identifying direct binding of nATF6α to the promoter region of the tumour suppressor FBP1^28^. Strong binding to *Hspa5* (encoding BiP), an inducible target of ATF6α, served as a positive control (Fig. 2j and Supplementary Fig. 4a,b). Assay for transposase-accessible chromatin using sequencing (ATAC–seq) analysis revealed reduced chromatin accessibility of the *Fbp1* promoter region in livers of *TG*^*Alb-cre+*^ versus *TG*^*Alb-cre−*^ mice (Fig. 2j (top) and Supplementary Fig. 4a,c), highlighting chromatin closure at the *Fbp1* locus after ATF6α activation.

## FBP1 counters nATF6α-altered metabolism

**Extended Data Fig. 3:**
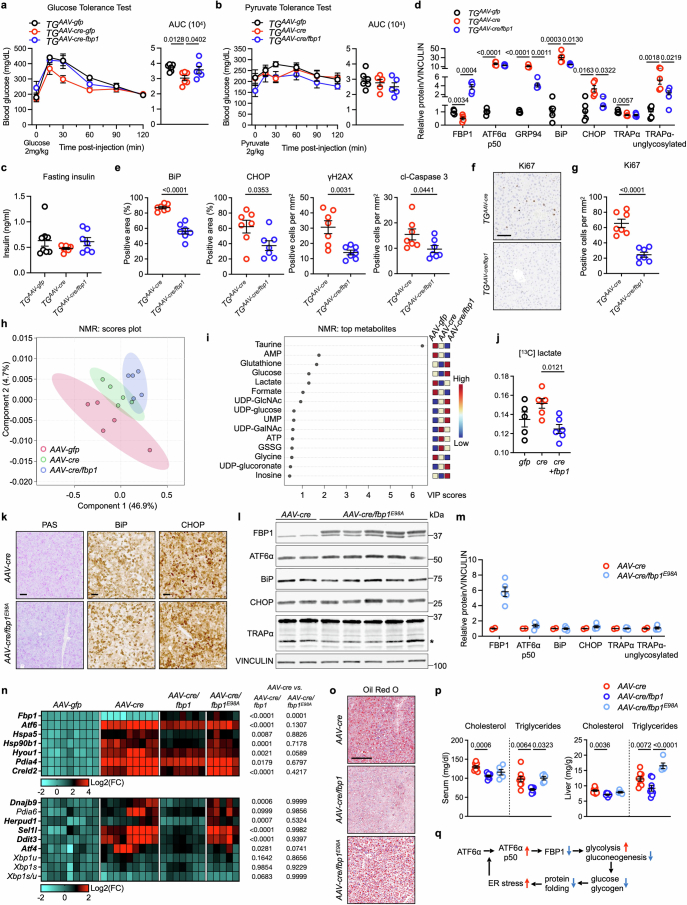
Catalytically functional FBP1 in hepatocytes mitigates ATF6α-activation-driven dysregulation of glucose- and lipid-metabolism, ER stress, and liver injury, Related to Fig. 2. (**a**) Glucose tolerance test of 16h-fasted *TG*^*AAV-gfp*^, *TG*^*AAV-cre*^, and *TG*^*AAV-cre/fbp1*^ mice (n = 6/group). (**b**) Pyruvate tolerance test and excursion of 16h-fasted *TG*^*AAV-gfp*^ (n = 6), *TG*^*AAV-cre*^ (n = 5), and *TG*^*AAV-cre/fbp1*^ (n = 5) mice. (**c**) Insulin levels of 16h-fasted *TG*^*AAV-gfp*^ (n = 8), *TG*^*AAV-cre*^ (n = 8), and *TG*^*AAV-cre/fbp1*^ (n = 7) mice measured by ELISA. (**d**) Quantification of immunoblot analysis from livers of *TG*^*AAV-gfp*^, *TG*^*AAV-cre*^, and *TG*^*AAV-cre/fbp1*^ mice in Fig. 2n (n = 5/group). (**e**) Quantification of IHC in Fig. 2o from livers of *TG*^*AAV-cre*^ and *TG*^*AAV-cre/fbp1*^ mice (n = 7/group). (**f-g**) IHC of Ki67 (**f**; scale bar = 100 µm) with related quantification (**g**) from livers of *TG*^*AAV-cre*^ and *TG*^*AAV-cre/fbp1*^ mice (n = 7/group). (**h**) NMR metabolomics partial least squares discriminant analysis (PLS − DA) of liver samples from *TG*^*AAV-gfp*^, *TG*^*AAV-cre*^, and *TG*^*AAV-cre/fbp1*^ mice (n = 5/group) with coloured 95% confidence intervals illustrating individual group separation. (**i**) Top 15 NMR metabolite concentration changes and their VIP score based on the PLS − DA regression model. (**j**) [^13^C]-labelled lactate uptake measured by GC-MS analysis from livers of *TG*^*AAV-gfp*^ (n = 5), *TG*^*AAV-cre*^ (n = 6), and *TG*^*AAV-cre/fbp1*^ (n = 6) mice. (**k**) Representative liver tissue showing similar PAS staining and IHC of BiP and CHOP expression in *TG*^*AAV-cre*^ and hepatocyte-specific FBP1-catalytically dead mutant *TG*^*AAV-cre/fbp1E98A*^ mice. Scale bar = 100 µm. (**l-m**) Representative immunoblot analysis (**l**) and quantification (**m**) of indicated proteins from livers of *TG*^*AAV-gfp*^ (n = 2) and *TG*^*AAV-cre/fbp1E98A*^ (n = 5) mice. VINCULIN as loading control, ran on FBP1 blot. kDa: kilodalton. (**n**) qRT-PCR analysis of indicated mRNAs from livers of *TG*^*AAV-gfp*^ (n = 9), *TG*^*AAV-cre*^ (n = 9), *TG*^*AAV-cre/fbp1*^ (n = 7), and *TG*^*AAV-cre/fbp1E98A*^ (n = 5) mice. Bold labelling depicts significant genes. (**o**) Representative liver Oil Red O staining for lipid accumulation from *TG*^*AAV-cre*^, *TG*^*AAV-cre/fbp1*^, and *TG*^*AAV-cre/fbp1E98A*^ mice (scale bar = 100 µm). (**p**) Total cholesterol or triglyceride levels measured in sera (left) or livers (right) of *TG*^*AAV-cre*^ (n = 9), *TG*^*AAV-cre/fbp1*^ (n = 8), and *TG*^*AAV-cre/fbp1E98A*^ (n = 5) mice. (**q**) Schematic by which ATF6α activation causes dysregulated glucose metabolism through FBP1 downregulation that perpetuates ER stress in a feed-forward loop. Scatter dot plot data and line graph data are presented as mean values ± SEM. Data in 3a-d,j,p were analysed by one-way ANOVA. Data in 3e,g were analysed by two-tailed unpaired *t*-test or Mann-Whitney test based on data normality distribution. Data in 3n were analysed by two-way ANOVA. Source data

Persistent ATF6α activation rendered *TG*^*Alb-cre+*^ mice more glucose tolerant, without affecting insulin tolerance, compared with *TG*^*Alb-cre−*^ mice that had similar locomotion, respiration, and food and water intake (Supplementary Fig. 4d–i). ATF6α activation by AAV8-cre transduction into hepatocytes induced hepatomegaly and liver injury after 2–3 weeks in *TG*^*AAV-cre*^ mice (Fig. 2k–m and Supplementary Fig. 4j–l). This was associated with ATF6α-dependent ER stress and deficient protein *N*-glycosylation, the latter caused by lack of glycogen/glucose, essential substrates for proper glycoprotein folding^29^. Accordingly, FBP1 levels were reduced in *TG*^*AAV-cre*^ versus *TG*^*AAV-gfp*^ mice after overnight fasting (Fig. 2n, Extended Data Fig. 3a–d and Supplementary Fig. 4m–o).

Given the direct repression of FBP1 by activated ATF6α, we hypothesized that restoring FBP1 could suppress glycolysis, reactivate gluconeogenesis and potentially prevent ATF6α-activation-mediated liver injury (Fig. 2k–p). AAV8-induced hepatocyte FBP1 reduced the liver-to-body weight, ALT–AST levels and glucose sensitivity without affecting gluconeogenesis or fasting insulin levels, while simultaneously limiting ATF6α-dependent signalling (that is, reduced ER stress, UPR induction, TRAPα hypoglycosylation) in the livers of *TG*^*AAV-cre*^ mice (Fig. 2k–n and Extended Data Fig. 3a–d). FBP1 re-expression in hepatocytes of *TG*^*AAV-cre*^ mice mitigated DNA damage, cell death and cell proliferation (Fig. 2o,p and Extended Data Fig. 3e–g). *TG*^*AAV-cre/fbp1*^ livers presented downregulated glycolysis and hypoxia in favour of oxidative phosphorylation (OXPHOS), reduced levels of taurine, known to promote glycolysis and metastasis in HCC^30^, increased glucose levels and reduced the uptake of ^13^C-labelled lactate compared with *TG*^*AAV-cre*^ livers (Fig. 2o,p and Extended Data Fig. 3h–j).

Cytosolic and catalytically active FBP1 is required for gluconeogenesis, whereas nuclear FBP1 inhibits hypoxia-inducible factors, independent of its enzymatic properties^31–33^. Catalytically active FBP1, but not the inactive mutant (*Fbp1*^*E98A*^)^28^, restored glycogen, limited induction of ATF6α targets in the liver, and reduced serum and liver lipid accumulation in *TG*^*AAV-cre*^ mice (Fig. 2o and Extended Data Fig. 3k–p). Decreased FBP1 activity prevents gluconeogenesis and accelerates glycolytic flux^31^, thereby depleting glycogen/glucose needed for protein *N*-glycosylation and ER function, further perpetuating ER stress and ATF6α activation (Extended Data Fig. 3q). Accordingly, FBP1 re-expression promoted gluconeogenesis, increased liver hexose phosphate levels and prevented the ATF6α-activation-driven increase in tricarboxylic-acid-cycle-related oncometabolites (Supplementary Fig. 5). Thus, metabolic reprogramming occurs through the repression of catalytically functional FBP1 in livers of mice with activated ATF6α.

## ATF6α activation causes mouse liver cancer

**Fig. 3:**
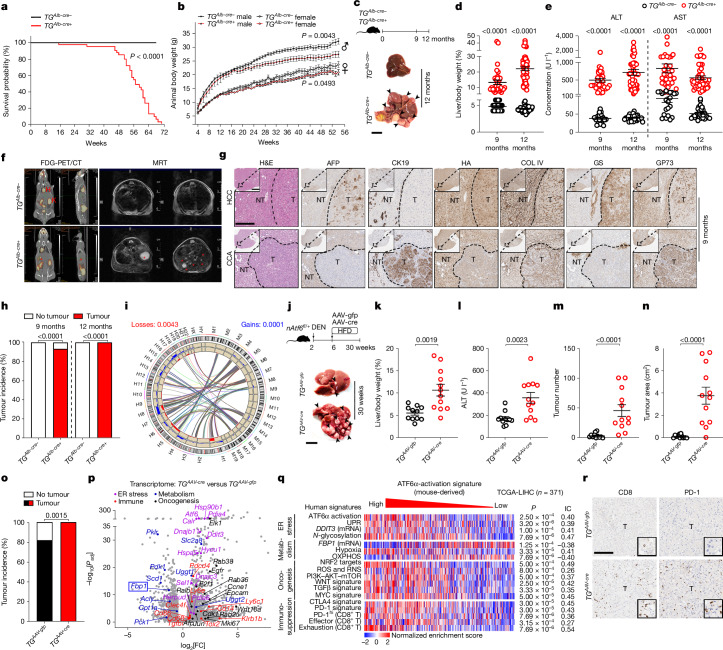
Prolonged ATF6α activation induces spontaneous, primary liver cancer in mice. **a**,**b**, Kaplan–Meier survival (**a**) and body-weight (**b**) curves of *TG*^*Alb-cre−*^ and *TG*^*Alb-cre+*^ mice. **c**, Schematic of *TG*^*Alb-cre−*^ and *TG*^*Alb-cre+*^ mice euthanized aged 9 and 12 months, the latter including liver images; the arrowheads indicate tumours. Scale bar, 1 cm. **d**,**e**, The liver-to-body weight (**d**) and serum ALT/AST levels (**e**) of 9- or 12-month-old *TG*^*Alb-cre−*^ and *TG*^*Alb-cre+*^ mice. **f**, Representative fluorodeoxyglucose positron emission tomography–computed tomography (FDG-PET/CT) (left; coronal and sagittal images from left to right) and magnetic resonance tomography (MRT) (right; axial images) of 9-month-old *TG*^*Alb-cre−*^ and *TG*^*Alb-cre+*^ mice. H, heart; K, kidney. The red asterisks denote tumour nodules/lesions. **g**, Representative haematoxylin and eosin (H&E) staining and IHC analysis of the indicated proteins in tumour or non-tumour liver from 9-month-old *TG*^*Alb-cre+*^ mice. Scale bar, 200 µm. **h**, The tumour incidence in 9- and 12-month-old *TG*^*Alb-cre+*^ and *TG*^*Alb-cre−*^ mice. **i**, Synteny analysis for chromosomal gains (blue) and losses (red) in 12-month-old *TG*^*Alb-cre+*^ mice (M1–19) and human HCC (H1–22). The outer circle represents mouse (M) and human (H) chromosomes. **j**, Schematic and liver images of *nAtf6*^*fl/+*^ mice intraperitoneally (i.p.) injected with DEN, followed by AAV8-gfp or AAV8-cre injection and HFD feeding. The arrowheads indicate tumours. Scale bar, 1 cm. **k**–**o**, The liver-to-body weight (**k**), serum ALT levels (**l**), tumour number (**m**) and area (**n**; cm^2^) per liver, and the tumour incidence (**o**) of DEN/HFD-treated *TG*^*AAV-gfp*^ and *TG*^*AAV-cre*^ mice. **p**, Liver RNA-seq analysis of DEN/HFD-treated *TG*^*AAV-cre*^ versus *TG*^*AAV-gfp*^ mice. **q**, Human gene sets expressed in TCGA-LIHC HCC samples were sorted by high to low enrichment of the mouse-derived ATF6α-activation signature. IC, information coefficient; RNS, reactive nitrogen species; ROS, reactive oxygen species. **r**, Representative liver IHC for CD8^+^ and PD-1^+^ in *TG*^*AAV-gfp*^ and *TG*^*AAV-cre*^ HCC samples. Scale bar, 200 µm. Quantification is shown in Extended Data Fig. 5n. The sample sizes, biological replicates and statistical tests are described in the Methods and Source data. Source data

**Extended Data Fig. 4:**
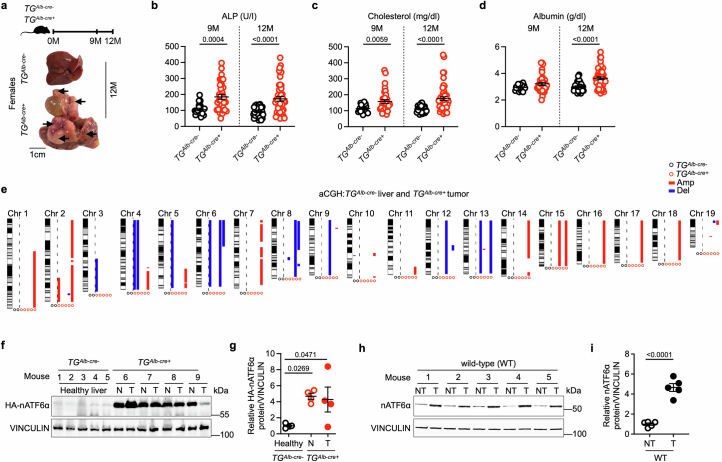
ATF6α activation spontaneously drives hepatocarcinogenesis, related to Fig. 3. (**a**) Scheme of *TG*^*Alb-cre−*^ and *TG*^*Alb-cre+*^ mice sacrificed at 9 or 12 months old (top) with representative liver macroscopy of 12-month-old female mice (bottom). (**b-d**) Serum ALP (**b**), cholesterol (**c**), and albumin (**d**) levels (9 M: *TG*^*Alb-cre−*^ n = 20 (10 males, 10 females); *TG*^*Alb-cre+*^ n = 33 (13 males, 20 females). 12 M: *TG*^*Alb-cre−*^ n = 29 (12 males, 17 females); *TG*^*Alb-cre+*^ n = 49 (25 males, 24 females)). (**e**) Genomic aberrations detected by aCGH of representative 12-month-old *TG*^*Alb-cre−*^ mouse livers and *TG*^*Alb-cre+*^ mouse tumour tissue. (**f-g**) Representative immunoblot analysis (**f**) and quantification (**g**) of HA-nATF6α in paired non-tumour liver (N) and tumour (T) lysates of *TG*^*Alb-cre−*^ (n = 5) and *TG*^*Alb-cre+*^ (n = 4) mice. VINCULIN as loading control, ran on a separate blot, processed in parallel. kDa: kilodalton. (**h-i**) Representative immunoblot analysis (**h**) and quantification (**i**) of nATF6α in paired non-tumour (NT) liver and tumour (T) lysates of wild-type (WT) mice injected with diethylnitrosamine (DEN; 25 mg/kg) at two weeks of age prior to high-fat diet (HFD) feeding and sacrificed at 38 weeks of age (n = 5 mice/group). VINCULIN as loading control, ran on nATF6α blot. Scatter dot plot data are presented as mean values ± SEM. Data in 4b-d,i were analysed by two-tailed unpaired *t*-test or Mann-Whitney test based on data normality distribution. Data in 4g were analysed by one-way ANOVA. Mouse icon was created in BioRender. Heikenwälder, M. (2026) https://BioRender.com/lgjnsy9. Source data

Considering the progression of chronic hepatitis to HCC, hepatocyte-specific ATF6α activation in *TG*^*Alb-cre+*^ mice reduced body-weight gain and lifespan compared with the *TG*^*Alb-cre−*^ controls due to spontaneous primary liver cancer (Fig. 3a–c). Hepatomegaly and liver injury persisted in 9- and 12-month-old *TG*^*Alb-cre+*^ mice (Fig. 3d,e and Extended Data Fig. 4a–d). Medical imaging detected tumour nodules in livers of 9-month-old *TG*^*Alb-cre+*^ mice, and subsequent dissection confirmed macroscopically visible liver tumours in 97.5% of them (Fig. 3f–h). Among the analysed tumours, 95% were HCC (AFP^+^, reduced intratumoural collagen type IV (COL IV), GS^+^, GP73^+^; nATF6α-HA^+^), and the remainder were cholangiocarcinoma (CCA; AFP^−^, CK19^+^) (Fig. 3g). Prolonged ATF6α activation caused primary liver cancer in all 12-month-old *TG*^*Alb-cre+*^ mice (Fig. 3h). Synteny analysis by array-based comparative genomic hybridization (aCGH) revealed that *TG*^*Alb-cre+*^ mice developed HCC with a comparable burden of chromosomal aberrations to human HCC (Fig. 3i and Extended Data Fig. 4e), and the extent of ATF6α activation in their livers/tumours was similar to that in human and mouse HCC (Fig. 1f,g and Extended Data Fig. 4f–i).

**Extended Data Fig. 5:**
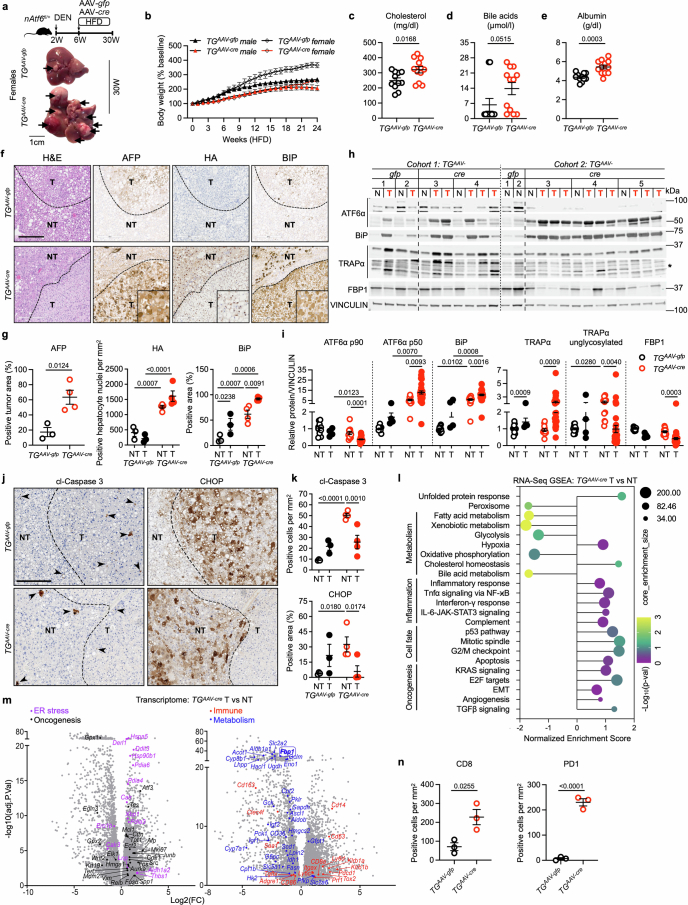
ATF6α activation accelerates hepatocarcinogenesis in a carcinogen and dietary preclinical model, related to Fig. 3. (**a**) Scheme of *nAtf6*^*fl/+*^ mice intraperitoneally injected with diethylnitrosamine (DEN; 25 mg/kg) prior to AAV8-*gfp* or AAV8-*cre* injection at the start of 60% high-fat diet (HFD) feeding and sacrificed at 30 weeks of age (top). Representative liver macroscopy of 30-week-old female mice (bottom). (**b**) Body weight gain of DEN/HFD-treated *TG*^*AAV-gfp*^ mice (n = 11; 6 males, 5 females) and *TG*^*AAV-cre*^ mice (n = 12; 7 males, 5 females). (**c-e**) Serum cholesterol (**c**), bile acids (**d**), and albumin (**e**) levels of DEN/HFD-treated *TG*^*AAV-gfp*^ (n = 11) and *TG*^*AAV-cre*^ (n = 12) mice. (**f-g**) Representative tumour (T) and non-tumour (NT) H&E staining and IHC for AFP, HA, BiP (**f**; scale bar = 300 µm), with quantification (**g**) from livers of *TG*^*AAV-gfp*^ (n = 3) and *TG*^*AAV-cre*^ (n = 4) mice. (**h-i**) Representative immunoblot analysis (**h**) and quantification (**i**) of indicated proteins from non-tumour (N) or tumour (T) liver lysates of *TG*^*AAV-gfp*^ (n = 11NT, with 4 T) and *TG*^*AAV-cre*^ mice (n = 12NT, with 31 T). VINCULIN as loading control, ran on a separate blot, processed in parallel. kDa: kilodalton. (**j-k**) Representative tumour (T) and non-tumour (NT) liver IHC of cl-Caspase 3 and CHOP (**j**; scale bar = 300 µm), with quantification (**k**) in *TG*^*AAV-gfp*^ (n = 3) and *TG*^*AAV-cre*^ (n = 4) mice. (**l**) GSEA of RNA-seq data shows negatively and positively enriched pathways in tumour (T) versus non-tumour (NT) livers of *TG*^*AAV-cre*^ mice (n = 5/group). (**m**) Volcano plot of RNA-seq data in tumour (T) versus paired non-tumour (NT) livers of *TG*^*AAV-cre*^ mice (n = 5/group). Purple, red, blue, and black dots represent ER-stress-related, immune-related, metabolic, and oncogenic genes, respectively. (**n**) Quantification of IHC in Fig. 3r from tumour tissue of *TG*^*AAV-gfp*^ and *TG*^*AAV-cre*^ mice (n = 3/group). Scatter dot plot data and line graph data are presented as mean values ± SEM. Data in 5c-e,g,n were analysed by two-tailed unpaired *t*-test or Mann-Whitney test based on data normality distribution. Data in 5g,i,k were analysed by two-way ANOVA. Data in 5l were analysed by GSEA based on the Kolmogorov-Smirnov test. Mouse icon was created in BioRender. Heikenwälder, M. (2026) https://BioRender.com/lgjnsy9. Source data

To investigate whether postnatal ATF6α activation (AAV8-cre) accelerates tumorigenesis, mice were subjected to diethylnitrosamine (DEN) and high-fat diet (HFD) feeding (DEN/HFD)^34^. Sustained ATF6α activation with DEN/HFD expedited hepatomegaly and liver damage, without significantly affecting body-weight gain in *TG*^*AAV-cre*^ versus *TG*^*AAV-gfp*^ mice (Fig. 3j–l and Extended Data Fig. 5a–e). Whereas 80% of *TG*^*AAV-gfp*^ mice presented early tumour lesions, 100% of *TG*^*AAV-cre*^ mice exhibited liver tumours, significantly greater in number, surface area and AFP positivity (Fig. 3m–o and Extended Data Fig. 5f,g). Tumours from *TG*^*AAV-cre*^ mice presented increased nuclear localization of ATF6α, together with elevated BiP and incompletely glycosylated TRAPα (Extended Data Fig. 5f–i). Non-tumour tissue showed an increase in apoptotic cells in *TG*^*AAV-cre*^ versus *TG*^*AAV-gfp*^ mice, while cleaved CASP3 and CHOP were significantly reduced in tumour versus non-tumour *TG*^*AAV-cre*^ liver (Extended Data Fig. 5j,k). In mouse livers with ATF6α activation, CHOP may induce UPR-specific hepatocyte death and promote compensatory proliferation, while established tumour cells downregulate CHOP to evade such death^35^. Transcriptomic analysis of DEN/HFD-treated *TG*^*AAV-cre*^ versus *TG*^*AAV-gfp*^ livers showed increased ER stress, inflammation and oncogenic signalling, with downregulated *Fbp1*, especially in tumour versus non-tumour *TG*^*AAV-cre*^ samples (Fig. 3p and Extended Data Fig. 5l,m).

A mouse-derived ATF6α-activation signature was custom-generated by single-sample GSEA (ssGSEA)^27^ with RNA-seq data from the livers of DEN/HFD-treated *TG*^*AAV-cre*^ versus *TG*^*AAV-gfp*^ mice to compare with human HCC (Fig. 3q). TCGA-LIHC samples sorted by high-to-low enrichment of the mouse-derived ATF6α-activation signature correlated with the human ATF6α-activation signature (Fig. 1), ER stress and UPR, *N*-glycosylation machinery, hypoxia, oncogenesis and immunosuppression (Fig. 3q). Consistent with the correlation between ATF6α activation and an immune-exhausted profile in human liver cancer (Fig. 1), tumour-infiltrating CD8^+^ T cells and PD-1^+^ cells were significantly increased in DEN/HFD-treated *TG*^*AAV-cre*^ versus *TG*^*AAV-gfp*^ livers (Fig. 3r and Extended Data Fig. 5n).

**Extended Data Fig. 6:**
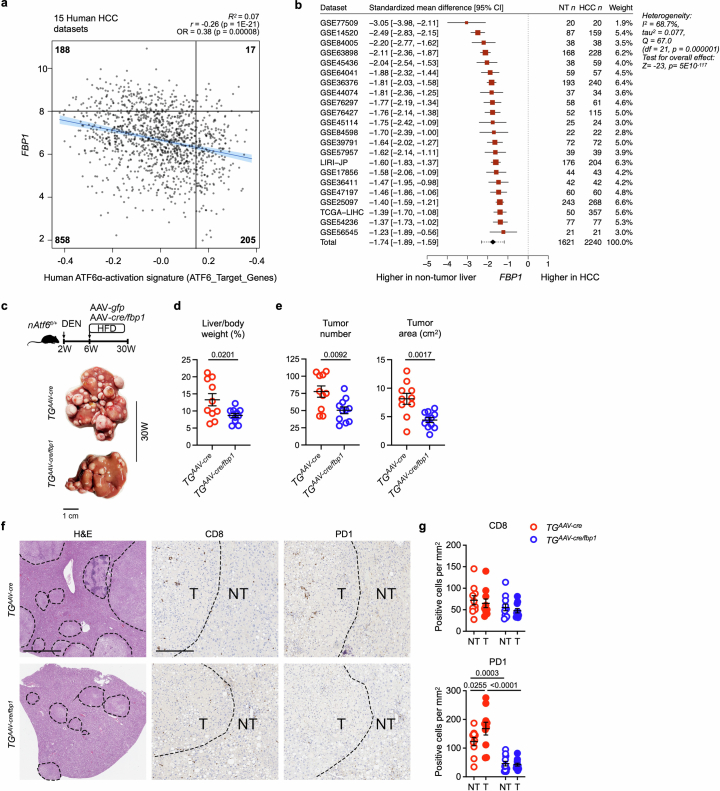
ATF6α activation in HCC is associated with downregulated FBP1, while restoring FBP1 reduces tumour burden and immunosuppression in mice, related to Fig. 3. (**a**) Correlation analysis and regression line of *FBP1* mRNA to the human ATF6α-activation signature (ATF6_Target_Genes) in 15 publicly available datasets. (**b**) Forest plot of *FBP1* mRNA expression in human HCC in relation to non-tumour liver tissue in 22 publicly available datasets. (**c**) Scheme (top) of DEN/HFD-treated *TG*^*AAV-cre*^ (n = 10) and *TG*^*AAV-cre/fbp1*^ (n = 11) mice, with representative macroscopic liver images at 30 weeks of age (bottom). (**d-e**) Liver-to-body weight (**d**), tumour number (**e**, left) and area (**e**, right, cm^2^) per liver of DEN/HFD-treated *TG*^*AAV-cre*^ (n = 10) and *TG*^*AAV-cre/fbp1*^ (n = 11) mice. (**f-g**) Representative liver H&E staining and IHC for CD8 and PD1 (**f**) with quantification (**g**) in tumour (T) versus paired non-tumour (NT) livers from DEN/HFD-treated *TG*^*AAV-cre*^ (n = 10) and *TG*^*AAV-cre/fbp1*^ (n = 11) mice. H&E: scale bar = 3 mm; IHC: scale bar = 300 µm. Scatter dot plot data are presented as mean values ± SEM. Data in 6a,b were computed by Pearson correlation coefficient and p-value based on a cut-off of +1 standard deviation from the mean using Fisher’s exact test. Data in 6d,e were analysed by two-tailed unpaired *t*-test based on data normality distribution. Data in 6g were analysed by two-way ANOVA. Mouse icon was created in BioRender. Heikenwälder, M. (2026) https://BioRender.com/lgjnsy9. Source data

In 15 datasets of patients with HCC, *FBP1* mRNA was inversely correlated with ATF6α-activation signatures and significantly reduced in human HCC versus non-tumour liver (Fig. 3q and Extended Data Fig. 6a,b). Restoring hepatic FBP1 expression in DEN/HFD-treated *TG*^*AAV-cre*^ mice significantly reduced ATF6α-activation-driven HCC development, as shown by reduced liver-to-body weight, tumour number and size (Extended Data Fig. 6c–e). Although the number of CD8^+^ T cells was similar between *TG*^*AAV-cre/fbp1*^ and *TG*^*AAV-cre*^ livers, FBP1 expression blunted PD-1^+^ cell accumulation in non-tumour and tumour tissue of *TG*^*AAV-cre*^ mice (Extended Data Fig. 6f,g). This suggests that CD8^+^ T cells were present but less exhausted in the livers of *TG*^*AAV-cre/fbp1*^ versus *TG*^*AAV-cre*^ mice.

## *Atf6* deletion reduces mouse HCC

**Fig. 4:**
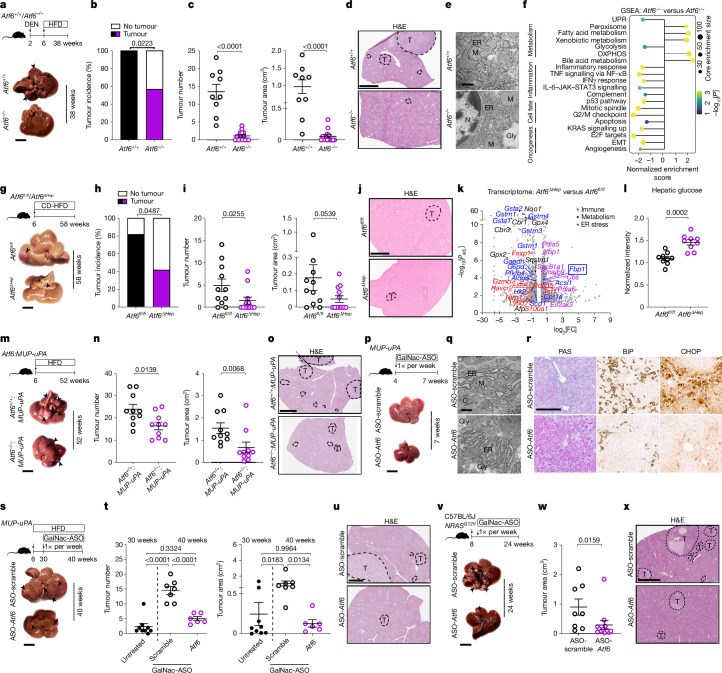
Prophylactic or therapeutic *Atf6* targeting reduces HCC. **a**, Schematic and liver images of DEN/HFD-treated *Atf6*^*+/+*^ and *Atf6*^*−/−*^ mice. Scale bar, 1 cm. **b**,**c**, The tumour incidence (**b**), number (**c**; left) and area (**c**; right, cm^2^) of DEN/HFD-treated *Atf6*^*+/+*^ and *Atf6*^*−/−*^ mice. **d**, Representative liver H&E staining of DEN/HFD-treated *Atf6*^*+/+*^ and *Atf6*^*−/−*^ mice. Scale bar, 3 mm. **e**, Representative liver TEM images of DEN/HFD-treated *Atf6*^*+/+*^ and *Atf6*^*−/−*^ mice. Scale bar, 1 µm. Quantification is shown in Extended Data Fig. 7h. Gly, glycogen. **f**, GSEA^27^ of liver tumour RNA-seq data from DEN/HFD-treated *Atf6*^*+/+*^ and *Atf6*^*−/−*^ mice. **g**, Schematic and liver images of CD-HFD-fed *Atf6*^*fl/fl*^ and *Atf6*^*ΔHep*^ mice. Scale bar, 1 cm. **h**,**i**, The tumour incidence (**h**), number and area (**i**; cm^2^) of CD-HFD-fed *Atf6*^*fl/fl*^ and *Atf6*^*ΔHep*^ mice. **j**, Representative liver H&E staining of CD-HFD-fed *Atf6*^*fl/fl*^ and *Atf6*^*ΔHep*^ mice. Scale bar, 3 mm. **k**, Volcano plot of liver RNA-seq data from *Atf6*^*ΔHep*^ versus *Atf6*^*fl/fl*^ mice. **l**, Liquid chromatography–mass spectrometry (LC–MS) analysis of hepatic glucose in *Atf6*^*fl/fl*^ and *Atf6*^*ΔHep*^ mice. **m**, Schematic and liver images of *Atf6*^*+/+*^ and *Atf6*^*−/−*^ mice crossed with *MUP-uPA* mice and fed a HFD. Scale bar, 1 cm. **n**, The tumour number (left) and area (right; cm^2^) of *Atf6*^*+/+*^*:MUP-uPA* and *Atf6*^*−/−*^*:MUP-uPA* mice. **o**, Representative liver H&E staining of HFD-fed *Atf6*^*+/+*^*:MUP-uPA* and *Atf6*^*−/−*^*:MUP-uPA* mice. Scale bar, 3 mm. **p**, Schematic and liver images of *MUP-uPA* mice administered GalNac-ASO-scramble or GalNac-ASO-*Atf6*. Scale bar, 1 cm. **q**, Representative liver TEM images of ASO-scramble- or ASO-*Atf6*-treated *MUP-uPA* mice. Scale bar, 500 nm. Quantification is shown in Extended Data Fig. 8f. C, collagen. **r**, Representative liver PAS staining and IHC for BiP and CHOP in ASO-scramble- or ASO-*Atf6*-treated *MUP-uPA* mice. Scale bar, 200 µm. Quantification is shown in Extended Data Fig. 8e. **s**, Schematic and liver images of HFD-fed *MUP-uPA* mice administered GalNac-ASO-scramble or GalNac-ASO-*Atf6*. Scale bar, 1 cm. **t**, The tumour number (left) and area (right) of 30-week-old untreated or 40-week-old ASO-scramble- or ASO-*Atf6*-treated *MUP-uPA* mice. **u**, Representative liver H&E staining of ASO-scramble- or ASO-*Atf6*-treated *MUP-uPA* mice. Scale bar, 3 mm. **v**, Schematic and liver images of GalNac-ASO-scramble- or GalNac-ASO-*Atf6*-treated C57BL/6J + *NRAS*^*G12V*^ mice. Scale bar, 1 cm. **w**, Tumour area (cm^2^) of ASO-scramble- or ASO-*Atf6*-treated C57BL/6J + *NRAS*^*G12V*^ mice. **x**, Representative liver H&E staining of ASO-scramble- or ASO-*Atf6*-treated C57BL/6J + *NRAS*^*G12V*^ mice. Scale bar, 3 mm. The sample sizes, biological replicates and statistical tests are described in the Methods and Source data. Source data

**Extended Data Fig. 7:**
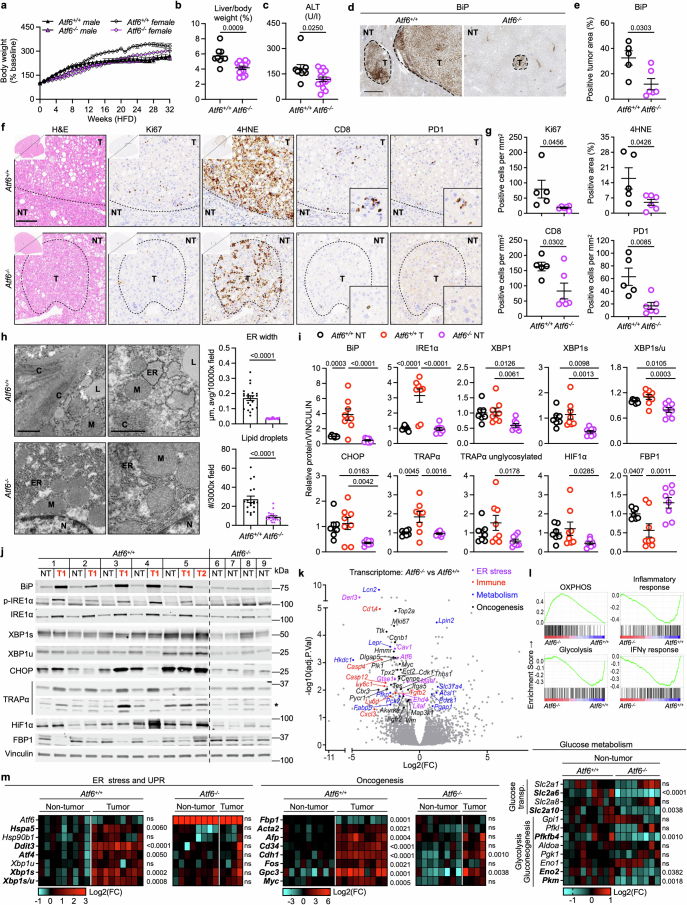
Targeting ATF6α activation protects against MASH-HCC hallmarks, Related to Fig. 4. (**a**) Body weight gain during HFD-feeding of *Atf6*^*+/+*^ (n = 9; 3 males, 6 females) and *Atf6*^−/−^ (n = 14; 9 males, 5 females) mice injected with diethylnitrosamine (DEN; 25 mg/kg) at 2 weeks of age and sacrificed at 38 weeks of age. (**b**) Liver-to-body weight of DEN/HFD-treated *Atf6*^*+/+*^ (n = 9) and *Atf6*^−/−^ (n = 14) mice. (**c**) Serum ALT levels of DEN/HFD-treated *Atf6*^*+/+*^ (n = 9) and *Atf6*^−/−^ (n = 14) mice. (**d-e**) Representative IHC (**d**, scale bar = 500 µm) and quantification (**e**) of BiP in liver tumour from DEN/HFD-treated *Atf6*^*+/+*^ (n = 5) and *Atf6*^−/−^ (n = 6) mice. (**f-g**) Representative H&E staining and IHC for Ki67, 4HNE, CD8, and PD1 (**f**; scale bar = 100 µm) in tumour (T) and non-tumour liver (NT) of DEN/HFD-treated *Atf6*^*+/+*^ (n = 5) and *Atf6*^−/−^ (n = 6) mice with intratumoral quantification (**g**). (**h**) TEM images and quantification of tumour tissue from DEN/HFD-treated *Atf6*^*+/+*^ or *Atf6*^−/−^ mice, as in Fig. 4e, with cellular structures denoted as: C, collagen; ER, endoplasmic reticulum; L, lipid droplets; M, mitochondria; N, nucleus (scale bar = 1 µm). ER width: n = 21 fields of view (FOV), 2 *Atf6*^*+/+*^ mice or n = 22 FOV, 2 *Atf6*^−/−^ mice; lipid droplets: n = 18 FOV, 2 *Atf6*^*+/+*^ mice or n = 19 FOV, 2 *Atf6*^−/−^ mice. (**i-j**) Quantification (**i**) of immunoblot analysis (**j**, representative) from DEN/HFD-treated *Atf6*^*+/+*^ (n = 7NT, with 8 T) and *Atf6*^−/−^ (n = 8NT) mouse liver lysates. VINCULIN as loading control, ran on FBP1 blot. kDa: kilodalton. (**k-l**) Volcano plot (**k**) of RNA-seq data with GSEA enrichment plots (**l**) from livers of DEN/HFD-treated *Atf6*^−/−^ versus *Atf6*^*+/+*^ mice (n = 3/group). Purple, red, blue, and black dots indicate ER stress, immune-related, metabolic and oncogenic genes, respectively. (**m**) qRT-PCR analysis of indicated mRNAs from livers/tumours of DEN/HFD-treated *Atf6*^*+/+*^ (n = 9NT, 9 T) and *Atf6*^−/−^ mice (n = 8NT, 4 T). Bold labelling depicts significant genes. Scatter dot plot data and line graph data are presented as mean values ± SEM. Data in 7b-c,e,g,h,m were analysed by two-tailed unpaired *t*-test or Mann-Whitney test based on data normality distribution. Data in 7i were analysed by one-way ANOVA. Source data

*Atf6*-deleted (encoding ATF6α) mice (*Atf6*^*−/−*^) and control littermates (*Atf6*^*+/+*^) were injected with DEN and fed a HFD for 32 weeks (Fig. 4a). *Atf6* deletion reduced liver-to-body weight and ALT levels, without affecting body-weight gain (Extended Data Fig. 7a–c). Whereas all *Atf6*^*+/+*^ mice developed steatotic tumours with ER stress, *Atf6* deletion mitigated lipid accumulation, ER stress and HCC incidence with reduced tumour numbers and size (Fig. 4b–e and Extended Data Fig. 7d–k). Liver IHC and intratumoural quantification revealed reduced tumour-cell proliferation, lipid peroxidation, as well as CD8^+^ T cell and PD-1^+^ cell infiltration in *Atf6*^−/−^ versus *Atf6*^*+/+*^ mice (Extended Data Fig. 7f,g), contrary to ATF6α activation in human and mouse HCC displaying increased tumour-cell proliferation and increased CD8^+^ T cell and PD-1^+^ cell infiltration (Figs. 1 and 3 and Extended Data Figs. 1 and 5). *Atf6* deletion inhibited UPR activation, hypoglycosylation of TRAPα and maintained FBP1 levels, therefore impeding pathogenic hepatic glycolysis, inflammation and oncogenic signalling (Fig. 4f and Extended Data Fig. 7i–m).

To investigate a cell-autonomous protective effect of *Atf6* deletion in HCC, hepatocyte-specific *Atf6*-knockout mice (*Atf6*^*ΔHep*^; Supplementary Table 1) were challenged in various preclinical models. *Atf6*^*ΔHep*^ mice fed a choline-deficient HFD (CD-HFD)^36^ for 58 weeks displayed significantly reduced liver-to-body weight, liver cancer incidence and tumour number compared with the control mice (Fig. 4g–j and Supplementary Fig. 6a,b). CD-HFD-fed *Atf6*^*ΔHep*^ mice had significantly higher liver *Fbp1* and reduced glycolysis pathway expression compared with *Atf6*^*fl/fl*^ mice, as determined using RNA-seq, mirroring an increase in hepatic glucose content and restored glycogen (Fig. 4k,l and Supplementary Fig. 6c). CD8^+^ T cells and PD-1^+^ cells were significantly reduced in the livers of *Atf6*^*ΔHep*^ versus *Atf6*^*fl/fl*^ mice (Supplementary Fig. 6d,e). These results were corroborated by Western diet (WD)-fed *Atf6*^*ΔHep*^ mice challenged with or without DEN. DEN/WD-treated *Atf6*^*ΔHep*^ mice displayed reduced tumour numbers compared with *Atf6*^*fl/fl*^ mice (Supplementary Fig. 6f–j). Despite unaltered steatosis, WD-fed *Atf6*^*ΔHep*^ mice presented reduced hepatomegaly and tumour incidence with significantly smaller and fewer HCC nodules (Supplementary Fig. 6k–p).

In *MUP-uPA* mice that develop ER-stress-driven MASH-HCC^37^ due to transiently high urokinase-type plasminogen activator (uPA, encoded by *Plau*) levels driven by the hepatocyte major urinary protein (MUP) promoter and HFD feeding, *Atf6* deletion reduced the liver-to-body weight, liver injury and tumour burden without affecting body-weight gain (Fig. 4m–o and Supplementary Fig. 6q–s). *Atf6* deletion in *MUP-uPA* mice reduced ER chaperones BiP (encoded by *Hspa5*) and *Hsp90b1* expression, but did not affect *Ddit3*, *Atf4* or *Xbp1* mRNA levels (Supplementary Fig. 6t–v), indicating no compensatory activation of the other UPR branches.

## *Atf6* ASOs limit ER stress and reduce HCC

**Extended Data Fig. 8:**
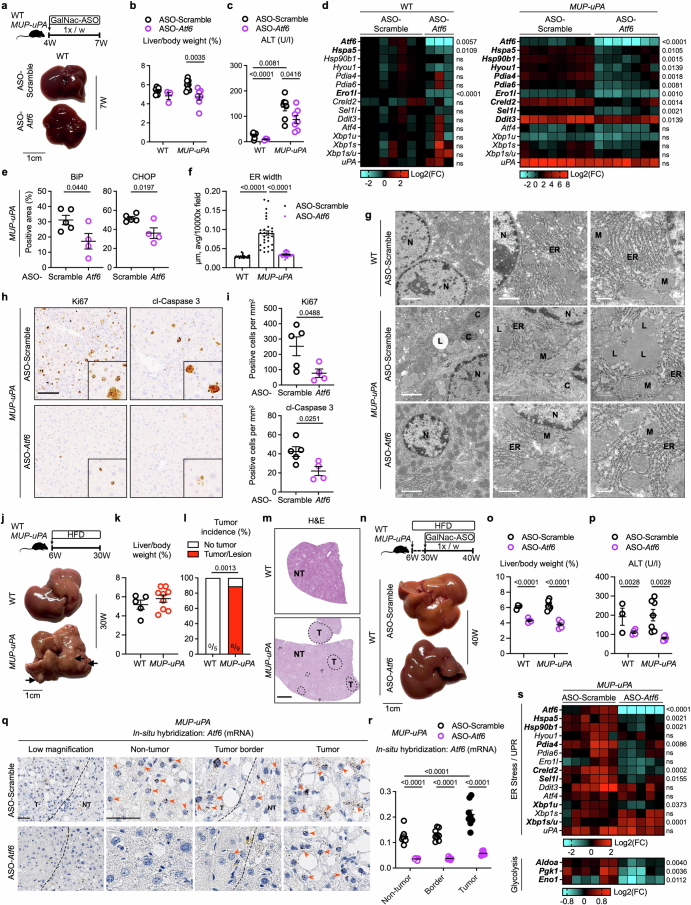
Targeting ATF6α activation by ASOs reduces ER-stress-related liver injury and HCC tumour burden in mice, Related to Fig. 4. (**a**) Scheme of wild-type (WT) or *MUP-uPA* mice receiving GalNac-conjugated ASO-Scramble or ASO-*Atf6* (2.5 mg/kg/week) for 3 weeks (top), with representative macroscopic liver images of WT mice (bottom). (**b-c**) Liver-to-body weight (**b**), and serum ALT levels (**c**) from livers of ASO-Scramble- or ASO-*Atf6*-treated WT mice (n = 8,3) or *MUP-uPA* mice (n = 8,7). (**d**) qRT-PCR analysis of indicated mRNAs from livers of ASO-Scramble- or ASO-*Atf6*-treated WT mice (n = 7,3) or *MUP-uPA* mice (n = 8,7). Bold labelling in genes depicts significant genes. (**e**) Quantification of IHC in Fig. 4r from livers of ASO-Scramble- (n = 5) or ASO-*Atf6-*treated (n = 4) *MUP-uPA* mice. (**f-g**) Quantification of ER width (**f**) and additional TEM images (**g**) from livers of ASO-Scramble-treated WT mice (n = 20 fields of view (FOV), 2 mice) and ASO-Scramble- (n = 30 FOV, 2 mice) or ASO-*Atf6*-treated (n = 33 FOV, 3 mice) *MUP-uPA* mice, as in Fig. 4q, with cellular structures denoted as: C, collagen; ER, endoplasmic reticulum; L, lipid droplets; M, mitochondria; N, nucleus (scale bar left to right = 2 µm, 1 µm, 500 nm). (**h-i**) Representative IHC of Ki67 and cl-Caspase 3 (**h**; scale bar = 100 µm), with quantification (**i**) from livers of ASO-Scramble- or ASO-*Atf6*-treated *MUP-uPA* mice (n = 5,4). (**j**) Scheme of *MUP-uPA* mice or WT mice fed a HFD and sacrificed at 30 weeks of age. Representative liver macroscopy of 30-week-old male mice (bottom). (**k-l**) Liver-to-body weight (**k**) and tumour incidence (**l**) of HFD-fed WT (n = 5) or *MUP-uPA* (n = 9) mice. (**m**) Representative H&E staining (scale bar = 2 mm) of livers from HFD-fed WT or *MUP-uPA* mice. (**n**) Scheme of *MUP-uPA* or WT mice administered GalNac-conjugated ASO-Scramble or ASO-*Atf6* during the last 10 weeks of HFD-feeding (top) with representative macroscopic liver images of WT mice (bottom). (**o-p**) Liver-to-body weight (**o**) and serum ALT levels (**p**) of ASO-Scramble- or ASO-*Atf6*-treated WT mice (n = 3,4) or *MUP-uPA* mice (n = 7,6) under HFD-feeding. (**q-r**) Representative *in-situ* hybridization (ISH) (**q**, scale bar = 50 µm) with quantification of *Atf6* mRNA intensity per field (**r**) in non-tumour (NT) liver, tumour border and tumour (T) of ASO-Scramble (n = 8) or ASO-*Atf6*-treated (n = 6) *MUP-uPA* mice under HFD-feeding. (**s**) qRT-PCR analysis of indicated mRNAs from livers of ASO-Scramble (n = 6) or ASO-*Atf6* (n = 5) treated *MUP-uPA* mice under HFD-feeding. Log_2_(Fold change) normalized to ASO-Scramble-treated WT mice. Scatter dot plot data are presented as mean values ± SEM. Data in 8b,c,o,p,r were analysed by two-way ANOVA. Data in 8d,e,k,s were analysed by two-tailed unpaired *t*-test or Mann-Whitney test based on data normality distribution. Data in 8f were analysed by one-way ANOVA. Data in 8l were analysed by Chi-square test for contingency. Mouse icons were created in BioRender. Heikenwälder, M. (2026) https://BioRender.com/lgjnsy9. Source data

Targeting ATF6α activation was further investigated using non-toxic *N*-acetylgalactosamine (GalNac)-conjugated antisense oligonucleotides (ASOs) that are preferentially taken up by hepatocytes and have high therapeutic potential in HCC^38^. ASOs against *Atf6* (GalNac-ASO-*Atf6*) or a scrambled nucleotide sequence (GalNac-ASO-scramble) were assessed in the *MUP-uPA* preclinical model (Fig. 4p). Early on, *MUP-uPA* mice exhibit liver injury due to strong, transient ER stress^37^ from 4 to 7 weeks of age that was abrogated by 85% knockdown of *Atf6* mRNA with hepatocyte-specific GalNac-ASO-*Atf6*, without affecting *uPA* levels (Fig. 4p–r and Extended Data Fig. 8a–g). GalNac-ASO-*Atf6* treatment in wild-type (WT) mice resulted in 89% *Atf6* mRNA knockdown with no observed adverse phenotype (Extended Data Fig. 8a–d). In *MUP-uPA* mice, reducing ATF6α activation prevented hepatic glycogen depletion, reduced BiP protein, as well as lipid accumulation, cell proliferation and cell death (Fig. 4r and Extended Data Fig. 8e–i). Targeting ATF6α protected against ER stress caused by uPA protein misfolding that activates all three UPR branches in *MUP-uPA* mice^37^.

At 30 weeks old, HFD-fed *MUP-uPA* mice display hepatocyte transformation and tumour progenitor cell development^37^, with nearly 90% of mice presenting macroscopically visible tumours (Extended Data Fig. 8j–m). GalNac-ASO-*Atf6* was administered to 30-week-old HFD-fed and tumour-bearing *MUP-uPA* mice until aged 40 weeks, when *MUP-uPA* mice exhibit steatohepatitic HCC^37^ and WT mice exhibit steatosis (Fig. 4s and Extended Data Fig. 8n). Hepatocyte-specific ATF6α targeting decreased the liver-to-body weight and serum ALT in both steatohepatitic *MUP-uPA* and steatotic WT mice (Extended Data Fig. 8o,p). Evaluated using in situ hybridization, GalNac-ASO-*Atf6* significantly reduced *Atf6* mRNA in hepatocytes and tumour cells of HFD-fed *MUP-uPA* mice by approximately 72% (Extended Data Fig. 8q,r). GalNac-ASO-*Atf6* downregulated *Atf6* without affecting *uPA* mRNA levels, downregulated ER stress, UPR and glycolysis targets, and significantly reduced the tumour burden in HFD-fed *MUP-uPA* mice (Fig. 4t,u and Extended Data Fig. 8s).

In another preclinical cancer model, hydrodynamic tail vein injection (HDTVi) of mutant *NRAS*^*G12V*^ plasmid accelerated tumour burden and depleted glycogen in livers of mice with activated ATF6α (Supplementary Fig. 7a–e). Conversely, targeting *Atf6* by GalNac-ASO-*Atf6* compared with GalNac-ASO-scramble reduced the liver tumour size of *NRAS*^*G12V*^-injected WT mice, prevented glycogen depletion and reduced PD-1^+^ cell abundance (Fig. 4v–x and Supplementary Fig. 7f–m). ATF6α may therefore be a therapeutic target for initiated HCC, leading to reduced tumour progression and reactivation of the natural anti-liver cancer immune response.

## Hepatocyte ATF6α drives immunosuppression

**Fig. 5:**
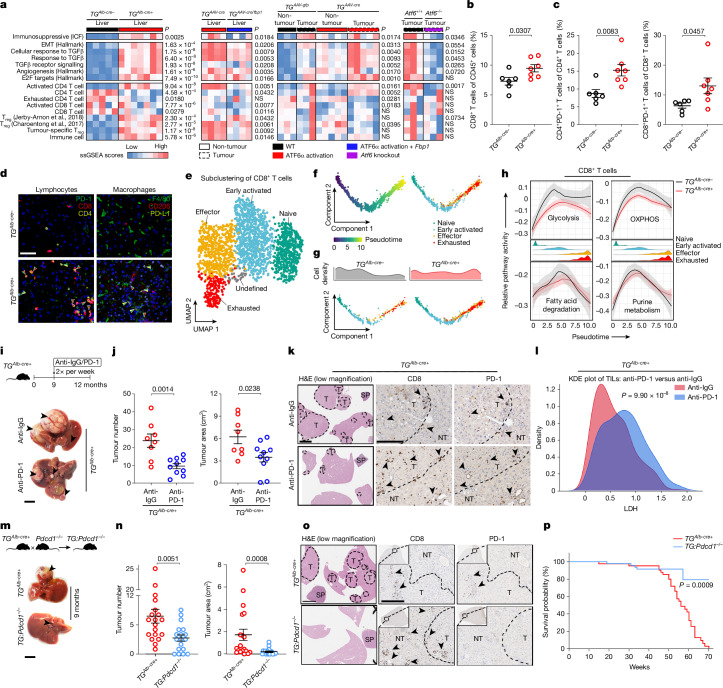
Hepatic ATF6α activation metabolically restricts anti-tumour immunosurveillance. **a**, Analysis of pro-tumorigenic ICF, TGFβ, immune cell or oncogenic signatures in 3-month-old *TG*^*Alb-cre−*^ and *TG*^*Alb-cre+*^ mice (Fig. 2a), 3-month-old *TG*^*AAV-cre*^ and *TG*^*AAV-cre/fbp1*^ mice (Fig. 2k), 30-week-old DEN/HFD-treated *TG*^*AAV-gfp*^ and *TG*^*AAV-cre*^ mice (Fig. 3j) and 38-week-old DEN/HFD-treated *Atf6*^*+/+*^ and *Atf6*^−/−^ mice (Fig. 4a). Additional data from refs. ^51,52^. NS, not significant. **b**,**c**, Fluorescence-activated cell sorting (FACS) analysis of the proportion of CD8^+^ T cells (**b**) and CD4^+^PD-1^+^ and CD8^+^PD-1^+^ T cells (**c**) in the livers of 6-month-old *TG*^*Alb-cre−*^ and *TG*^*Alb-cre+*^ mice. **d**, Representative liver immunofluorescence for PD-1, CD8 and CD4 in lymphocytes (left) and F4/80, CD206 and PD-L1 in macrophages (right) in 6-month-old *TG*^*Alb-cre−*^ and *TG*^*Alb-cre+*^ mice. The arrowheads indicate double-stained cells. Scale bar, 50 µm. Quantification is shown in Extended Data Fig. 9i. **e**,**f**, scRNA-seq UMAP (**e**) and trajectory analysis (**f**; left) of subclustered CD8^+^ T cells from the livers of 6-month-old *TG*^*Alb-cre−*^ and *TG*^*Alb-cre+*^ mice, with pseudotime ordering predicting CD8^+^ T cell development by subtype (**f**; right). **g**,**h**, Pseudotime-ordered CD8^+^ T cells (**g**; bottom) with state-wise density distribution (**g**; top), and pseudotime-based metabolic pathway activity of CD8^+^ T cells (**h**) from livers of 6-month-old *TG*^*Alb-cre−*^ and *TG*^*Alb-cre+*^ mice. **i**, Schematic and liver images of anti-IgG and anti-PD-1-treated *TG*^*Alb-cre+*^ mice. Scale bar, 1 cm. **j**, The tumour number (left) and area (right; cm^2^) of anti-IgG and anti-PD-1-treated *TG*^*Alb-cre+*^ mice. **k**, Representative liver H&E staining (scale bar, 3 mm) and IHC for CD8 or PD-1 (scale bar, 200 µm) in anti-IgG and anti-PD-1-treated *TG*^*Alb-cre+*^ mice. Quantification is shown in Extended Data Fig. 11b. **l**, Kernel density estimation (KDE) plot for LDH expression in TILs from the livers of anti-IgG and PD-1-treated *TG*^*Alb-cre+*^ mice. **m**, The breeding strategy and liver images of 9-month-old *TG:Pdcd1*^−/−^ mice. Scale bar, 1 cm. **n**, The tumour number (left) and area (right; cm^2^) of 9-month-old *TG:Pdcd1*^−/−^ mice. **o**, Representative liver H&E staining (scale bar, 3 mm) and IHC analysis of CD8 or PD-1 (scale bar, 200 µm) in 9-month-old *TG:Pdcd1*^−/−^ mice. Quantification is shown in Extended Data Fig. 12j. **p**, Kaplan–Meier survival curves of *TG*^*Alb-cre+*^ (*n* = 43) and *TG:Pdcd1*^−/−^ mice (*n* = 53). The sample sizes, biological replicates and statistical tests are described in the Methods and Source data. Source data

**Extended Data Fig. 9:**
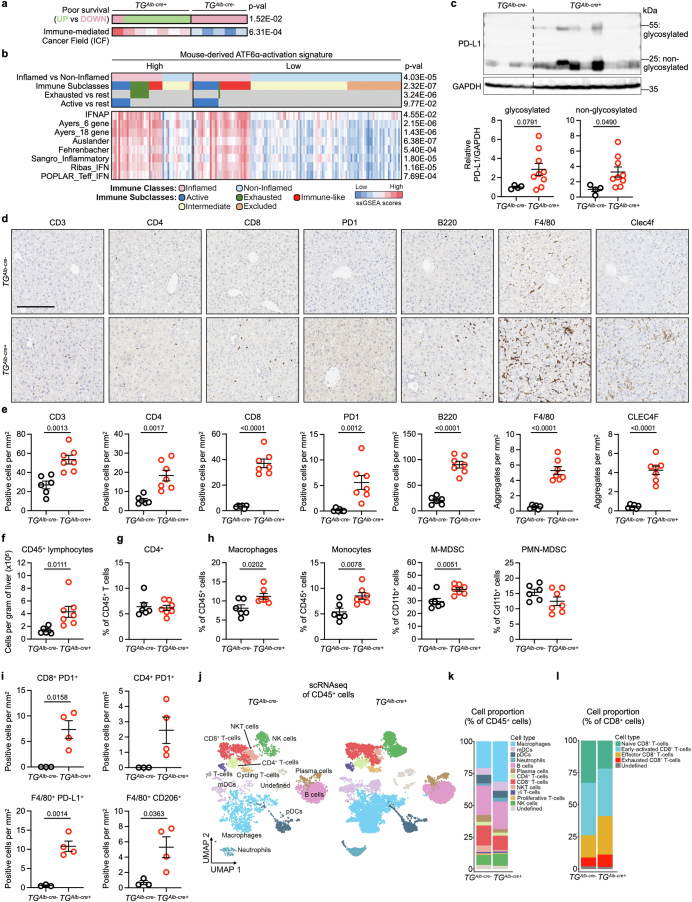
Prolonged ATF6α activation in hepatocytes is associated with an immunosuppressive liver microenvironment in mice, Related to Fig. 5. (**a**) Gene signatures predicting poor survival and Immune-mediated Cancer Field (ICF) signature analysed in livers of 3-month-old *TG*^*Alb-cre−*^ (n = 5) and *TG*^*Alb-cre+*^ (n = 7) mice, as shown in Fig. 5a. (**b**) Heatmap of immune features and signatures related to immune checkpoint blockade (ICB) response in HCC patients (n = 171^19^) divided into two groups by high and low enrichment of the mouse-derived ATF6α-activation signature established from RNA-seq data of DEN/HFD-treated *TG*^*AAV-cre*^ and *TG*^*AAV-gfp*^ mice (see Methods). (**c**) Representative immunoblot analysis and quantification of PD-L1 expression from livers of 6-month-old *TG*^*Alb-cre−*^ (n = 4) and *TG*^*Alb-cre+*^ (n = 9) mice. GAPDH as loading control, ran on PD-L1 blot. kDa: kilodalton. (**d-e**) Representative IHC (**d**; scale bar = 100 µm) with quantification (**e**) from livers of 6-month-old *TG*^*Alb-cre−*^ (n = 6) and *TG*^*Alb-cre+*^ (n = 7) mice. (**f**) Liver CD45^+^ lymphocytes count (per gram of liver) in 6-month-old *TG*^*Alb-cre−*^ (n = 6) and *TG*^*Alb-cre+*^ (n = 7) mice by FACS. (**g-h**) The proportion of CD4^+^ T-cells (**g**) and macrophages, monocytes, monocytic- or polymorphonuclear myeloid-derived suppressor cells (M/PMN-MDSC, **h**) in livers of *TG*^*Alb-cre−*^ (n = 6) and *TG*^*Alb-cre+*^ (n = 7) mice. (**i**) Quantification of immunofluorescence staining in Fig. 5d of livers from *TG*^*Alb-cre−*^ (n = 3) and *TG*^*Alb-cre+*^ (n = 4) mice. (**j-k**) scRNA-seq UMAP representations of immune cell populations from FACS-sorted CD45^+^ cells (**j**) and respective cell-type distribution (**k**) from livers of 6-month-old *TG*^*Alb-cre−*^ and *TG*^*Alb-cre+*^ mice (n = 4/group). (**l**) scRNA-seq cell-type distribution of subclustered CD8^+^ T-cells (Fig. 5e) from livers of 6-month-old *TG*^*Alb-cre−*^ and *TG*^*Alb-cre+*^ mice (n = 4/group). Scatter dot plot data are presented as mean values ± SEM. Data in 9a were analysed using the nonparametric Wilcoxon and Kruskal-Wallis tests. Data in 9b with continuous variables were analysed by two-tailed Student’s *t*-test, and categorical variables were analysed by Fisher’s exact test. Data in 9c,e-i were analysed by two-tailed unpaired *t*-test or Mann-Whitney test based on data normality distribution. Source data

Transcriptional analyses were performed for immune-mediated cancer field (ICF) signatures^39^, defined by 172 genes for deregulated immune response associated with HCC. Livers of 3-month-old *TG*^*Alb-cre+*^ mice already presented significantly higher ICF signature scores, related to immunosuppression^39^, TGFβ-activation and T_reg_-cell-signature enrichment, while restoring FBP1 expression limited many of the above-mentioned signatures (Fig. 5a and Extended Data Fig. 9a). In tumours from 171 patients with HCC^19^, the mouse-derived ATF6α-activation signature was enriched in inflamed tumours, particularly those of the immune-exhausted subclass (23%; Extended Data Fig. 9b). Higher scores for predicted ICB response, which included a novel 11-gene signature in frontline advanced HCC (IFNAP)^25^, were enriched in human ATF6α^hi^ HCCs (Fig. 1r and Extended Data Fig. 9b). Liver tumours of DEN/HFD-treated transgenic mice with activated ATF6α had enriched oncogenic (for example, angiogenesis and E2F targets) and immunosuppressive (for example, ICF and TGFβ activation) signatures, in contrast to *Atf6*-knockout mice (Fig. 5a). ATF6α-activation-driven metabolic dysregulation may therefore precede inflammation, both predisposing to hepatocarcinogenesis.

At the 6-month-old precancerous stage, chronically activated ATF6α increased hepatic PD-L1 levels (Extended Data Fig. 9c), which is known to suppress T cell function^40^. A significant increase in liver-infiltrating immune cells was detected by flow cytometry, immunofluorescence and IHC analysis of *TG*^*Alb-cre+*^ versus *TG*^*Alb-cre−*^ livers (Fig. 5b–d and Extended Data Fig. 9d–h). Cells manifesting immunosuppression, including monocytic myeloid-derived suppressor cells (M-MDSCs), PD-1^+^ T cells, and CD206^+^ and PD-L1^+^ macrophages, were significantly increased in *TG*^*Alb-cre+*^ livers (Fig. 5c,d, Extended Data Fig. 9h,i and Supplementary Fig. 10a–c), consistent with human HCC (Fig. 1o–q). Overall, persistent, hepatocyte-specific ATF6α activation promotes early-onset liver injury with progressive oncogenic signalling in an inflamed, immunosuppressive microenvironment.

## Hepatocyte ATF6α limits T cell function

To elucidate how hepatocyte-specific ATF6α activation impacts neighbouring immune cells, we performed single-cell RNA-seq (scRNA-seq) analysis of sorted CD45^+^ leukocytes, which were more abundant and enriched in innate immune cell populations in *TG*^*Alb-cre+*^ than in *TG*^*Alb-cre−*^ livers (Extended Data Fig. 9j,k and Supplementary Fig. 8a). Subclustering of CD8^+^ T cells revealed increased effector and exhausted CD8^+^ T cells in *TG*^*Alb-cre+*^ livers (Fig. 5e and Extended Data Fig. 9l), consistent with human HCC (Fig. 1p,q) and transcriptomic characterization (Fig. 5a). This indicates an accelerated recruitment or local proliferation of naive CD8^+^ T cells concomitant with exhausted and immunosuppressive features (Fig. 5f,g and Supplementary Fig. 8b). As anti-tumour immunosurveillance relies on glycolysis and OXPHOS as energy sources in cytotoxic T cells^6^, we predicted the glucose-deprived liver microenvironment generated by hepatocyte-specific ATF6α activation hampers surrounding CD8^+^ T cell function. In contrast to other metabolic pathways, glycolysis and OXPHOS pathway activity were reduced in CD8^+^ T cells from *TG*^*Alb-cre+*^ versus *TG*^*Alb-cre−*^ livers (Fig. 5h and Supplementary Fig. 8c). CD4^+^ T cell distribution and metabolic pathway expression were similar between *TG*^*Alb-cre+*^ and *TG*^*Alb-cre−*^ livers (Supplementary Fig. 8d–g). *TG*^*Alb-cre+*^ livers demonstrated substantial repopulation of myeloid cells to compensate for the loss of Kupffer cells, commonly found in hepatitis^41^ (Supplementary Fig. 8h–j). Glucose-related metabolism in myeloid cells remained relatively unchanged between *TG*^*Alb-cre+*^ and *TG*^*Alb-cre−*^ livers (Supplementary Fig. 8k), suggesting that the glucose-deprived microenvironment predominantly affected glucose-sensitive CD8^+^ T cells^42^.

**Extended Data Fig. 10:**
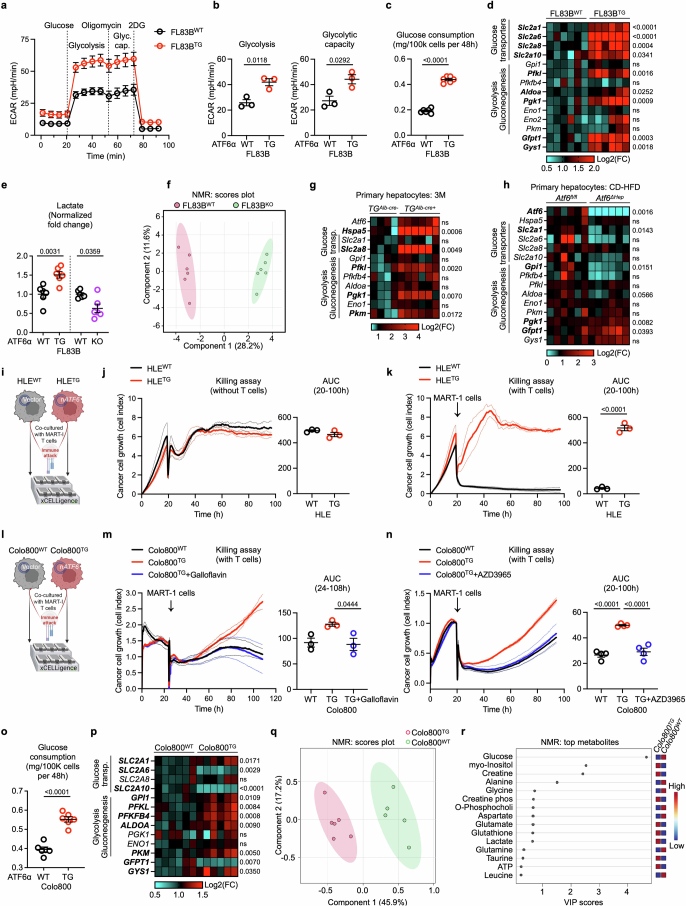
ATF6α activation metabolically renders cancer cells resistant to T-cell-mediated cytotoxicity, Related to Fig. 5. (**a-b**) Metabolic flux analysis (**a**) for extracellular acidification rates (ECAR) of FL83B^WT^ (n = 3) or FL83B^TG^ (n = 3) hepatocytes, with quantification (**b**) for glycolysis and glycolytic capacity. (**c**) Glucose consumption in FL83B^WT^ or FL83B^TG^ hepatocytes cultured for 48 h (n = 6/group). (**d**) qRT-PCR analysis of glucose-related metabolic genes in FL83B^WT^ or FL83B^TG^ cells (n = 6/group). Bold labelling depicts significant genes. (**e**) NMR spectroscopy-based metabolomics analysis of lactate concentration in FL83B cells with ATF6α expression (FL83B^TG^) or deletion (FL83B^KO^), normalized to respective controls (n = 6/group). (**f**) NMR metabolomics PLS − DA with coloured 95% confidence intervals illustrating the clear group separation in metabolic profile between FL83B^WT^ and FL83B^TG^ cells (n = 6/group). (**g**) qRT-PCR analysis of primary hepatocytes harvested from livers of 3-month-old *TG*^*Alb-cre−*^ (n = 4) and *TG*^*Alb-cre+*^ (n = 6) mice. Bold labelling depicts significant genes. (**h**) qRT-PCR analysis of primary hepatocytes harvested from livers of CD-HFD-fed *Atf6*^*fl/fl*^ (n = 6) and *Atf6*^*ΔHep*^ (n = 6) mice. Bold labelling depicts significant genes. (**i**) Scheme of HCC cancer cell line (HLE) stably transfected with control vector (HLE^WT^) or nATF6α-expressing (HLE^TG^) plasmid and co-cultured with or without cytotoxic MART-1 T-cells to assess immune attack by a real-time cell analyzer (xCELLigence). (**j**) HLE^WT^ or HLE^TG^ cancer cell growth over time with quantification on the right panel (n = 3/group). (**k**) Killing assay and quantification for HLE^WT^ or HLE^TG^ cancer cell growth in co-culture conditions with cytotoxic MART-1 T-cells (n = 3/group). (**l**) Scheme of Colo800 tumour cells stably transfected with control (Colo800^WT^) or nATF6α-expressing (Colo800^TG^) plasmid and co-cultured with cytotoxic MART-1-specific T-cells to assess immune attack in a real-time cell analyzer (xCELLigence). (**m**) Killing assay of Colo800^WT^ or Colo800^TG^ tumour cells in co-culture conditions with cytotoxic MART-1-specific T-cells (n = 3/group) in the presence or absence of the lactate dehydrogenase inhibitor galloflavin. (**n**) Killing assay for Colo800^WT^ or Colo800^TG^ tumour cell growth in co-culture conditions with cytotoxic MART-1 T-cells (n = 4/group) in the presence or absence of the lactate efflux inhibitor AZD3965. (**o**) Glucose consumption of Colo800^WT^ and Colo800^TG^ cells cultured for 48 h (n = 6/group). (**p**) qRT-PCR analysis of indicated mRNAs from Colo800^WT^ and Colo800^TG^ cells (n = 6/group). Bold labelling depicts significant genes. (**q**) NMR metabolomics PLS − DA with coloured 95% confidence intervals illustrating the clear group separation in metabolic profile between Colo800^WT^ (n = 5) and Colo800^TG^ (n = 6) cells. (**r**) Top 15 metabolites and their VIP score based on the PLS − DA regression model. Scatter dot plot data are presented as mean values ± SEM. Line graph data are presented as mean values ± SEM (10a) or mean values ± SD (10j-k, m-n). Data in 10b-d,g,h,o,p were analysed by two-tailed unpaired *t*-test or Mann-Whitney test based on data normality distribution. Data in 10e were analysed by one-way ANOVA. Data in 10j,k were calculated for the area under the curve and analysed by two-tailed Student’s *t*-test. Data in 10m,n were calculated for the area under the curve and analysed by one-way ANOVA. In vitro co-culture schematics were created in BioRender. Heikenwälder, M. (2026) https://BioRender.com/lgjnsy9. Source data

We next addressed whether activated ATF6α sufficed to induce a cell non-autonomous immunosuppressive environment. Activated ATF6α expression in mouse FL83B hepatocytes (FL83B^TG^) significantly increased glucose consumption, lactate production, glycolysis and glycolytic capacity compared with FL83B^WT^ hepatocytes (Extended Data Fig. 10a–e). By contrast, *Atf6-*deleted FL83B hepatocytes (FL83B^KO^) displayed significantly reduced lactate production (Extended Data Fig. 10e,f). Primary hepatocytes with activated ATF6α from chow-fed *TG*^*Alb-cre+*^ mice compared with *TG*^*Alb-cre−*^ mice had significantly upregulated glycolysis-related genes, while hepatocytes collected from CD-HFD-fed *Atf6*^*ΔHep*^ compared with *Atf6*^*fl/fl*^ mice showed the opposite (Extended Data Fig. 10g,h).

In an in vitro co-culture system, HLE or Colo800 cell lines with stable nATF6α expression demonstrated resistance to MART-1-specific T-cell-mediated killing (Extended Data Fig. 10i–n), corroborating that ATF6α activation induces immunosuppression. Inhibiting lactate dehydrogenase activity with galloflavin or lactate efflux with AZD3965 restored the killing efficiency of Colo800^TG^ cells by MART-1-specific T cells (Extended Data Fig. 10m,n). Indeed, untreated Colo800^TG^ cells exhibited higher glucose consumption and lactate production compared with Colo800^WT^ cells (Extended Data Fig. 10o–r). Thus, hepatocyte-specific ATF6α activation metabolically restricts T-cell-mediated anti-tumour immunosurveillance, probably through lactate production (among others) in a cell non-autonomous manner.

## ATF6α activation sensitizes HCC to ICB

With prognostic potential for guiding HCC immunotherapy, ATF6α activation promotes immunosuppressive features (MDSCs, CD8^+^PD-1^+^, CD8^+^PD-1^+^TIM3^+^ T cells)^7^ and an ICB-responsive tumour microenvironment characterized by: (1) hub-like immune niches (CD8^+^ T cells clustered with CD11c^+^ DCs and CD4^+^ T cells) and (2) spatial segregation of ICB-responsive CD8^+^TCF1^+^ T cells from immunosuppressive T_reg_ cells^23,24^.

**Extended Data Fig. 11:**
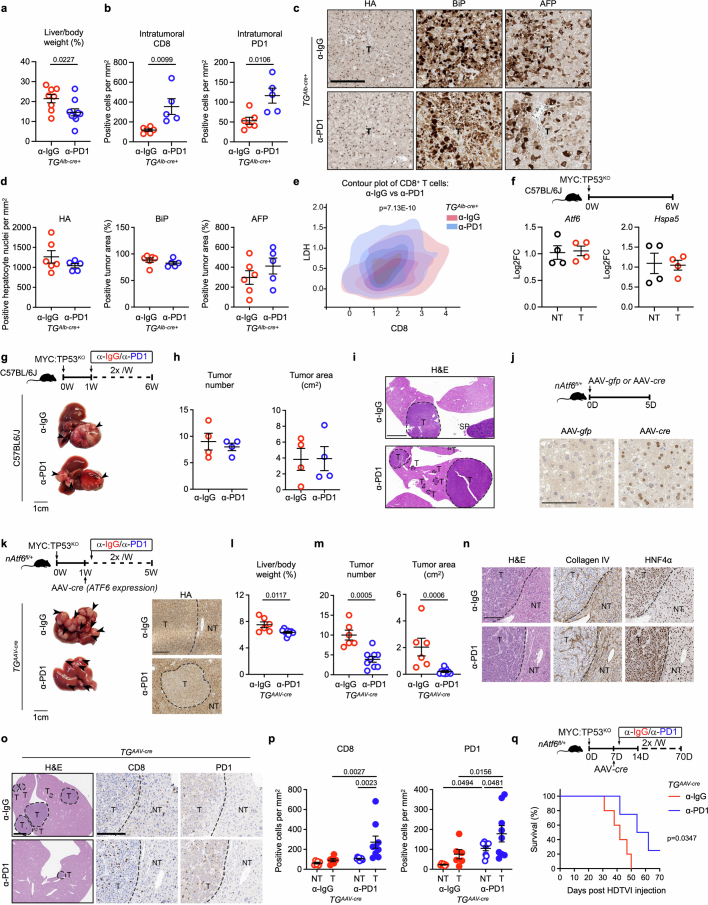
Anti-PD1 immune checkpoint blockade enhances T-cell-mediated anti-tumour immunosurveillance in livers with ATF6α activation, Related to Fig. 5. (**a**) Liver-to-body weight of 9-month-old *TG*^*Alb-cre+*^ mice treated with α-IgG (n = 8) or α-PD1 (n = 10) antibody. (**b**) IHC quantification for CD8^+^ and PD1^+^ cells in Fig. 5k from livers of *TG*^*AAV-cre*^ mice treated with α-IgG (n = 6) or α-PD1 (n = 5) monotherapy. (**c-d**) Representative liver tumour (T) IHC (**c**) and quantification (**d**) of HA, CHOP, BiP, and AFP (scale bar = 100 µm) from *TG*^*Alb-cre+*^ mice treated with α-IgG (n = 6) or α-PD1 (n = 5) monotherapy. (**e**) Contour plot for LDH expression in CD8^+^ TILs in α-IgG/PD1-treated *TG*^*Alb-cre+*^ mice by MIBI (n = 3/group). (**f**) Experimental scheme (top), and qRT-PCR analysis of *Atf6* or *Hspa5* mRNA expression in non-tumour liver (NT) or tumour (T) samples from C57BL6/J (WT) mice after HDTVi of MYC:TP53^KO^ plasmid DNA (n = 4/group). (**g**) Scheme depicts C57BL6/J mice treated with α-IgG or α-PD1 monotherapy after HDTVi of MYC:TP53^KO^ plasmid DNA (top) with representative, macroscopic liver images at sacrifice (bottom). (**h**) Tumour number (left) and area (right, cm^2^) per liver of α-IgG/PD1-treated C57BL6/J (WT) mice (n = 4/group). (**i**) Representative H&E staining (low magnification) of livers from α-IgG/PD1-treated C57BL6/J (WT) mice. Scale bar = 3 mm. (**j**) Scheme of *nATF6*^*fl/+*^ mice administered AAV8*-gfp* or AAV8*-cre* and sacrificed 5 days after injection, with representative liver HA IHC (bottom, scale bar = 100μm). (**k**) Scheme (top) with representative, macroscopic liver images (bottom left) and liver IHC for HA (bottom right) from *nATF6*^*fl/+*^ mice administered AAV8-*cre* (*TG*^*AAV-cre*^) and treated with α-IgG/PD1 monotherapy after HDTVi of MYC:TP53^KO^ plasmid DNA, scale bar = 100μm. (**l-m**) Liver-to-body weight (**l**), tumour number and area (**m**, cm^2^) of *TG*^*AAV-cre*^ mice treated with α-IgG (n = 6) or α-PD1 (n = 9) monotherapy after HDTVi of MYC:TP53^KO^ plasmid DNA. (**n**) Representative H&E staining and IHC for Collagen IV and HNF4α from livers of *TG*^*AAV-cre*^ mice treated with α-IgG/PD1 after HDTVi of MYC:TP53^KO^ plasmid DNA, scale bar = 200μm for IHC. (**o-p**) Representative H&E staining (low magnification) and IHC for CD8 and PD1 (**o**) with quantification (**p**) in livers of *TG*^*AAV-cre*^ mice treated with α-IgG (n = 6) or α-PD1 (n = 9) monotherapy after HDTVi of MYC:TP53^KO^ plasmid DNA. (**q**) Scheme (top) and Kaplan-Meier survival curves (bottom) of *TG*^*AAV-cre*^ mice treated with α-IgG (n = 5) or α-PD1 (n = 4) monotherapy after HDTVi of MYC:TP53^KO^ plasmid DNA. Scatter dot plot data are presented as mean values ± SEM. Data in 11a,b,d,f,h,l,m were analysed by two-tailed unpaired *t*-test or Mann-Whitney test based on data normality distribution. Data in 11e were analysed by Mann-Whitney test. Data in 11p were analysed by two-way ANOVA. Data in 11q were analysed by log-rank (Mantel-Cox) test. NT: non-tumour, T: tumour, SP: spleen. Mouse icons were created in BioRender. Heikenwälder, M. (2026) https://BioRender.com/lgjnsy9. Source data

Anti-PD-1 neutralizing antibody was therapeutically administered to 9-month-old *TG*^*Alb-cre+*^ mice, all presenting spontaneous liver cancer (Fig. 5i). Anti-PD-1 treatment in *TG*^*Alb-cre+*^ mice reduced tumour number and size (Fig. 5j), as well as liver-to-body weight (Extended Data Fig. 11a). IHC analysis revealed increased CD8^+^ T cell accumulation in both non-tumour liver and tumours of anti-PD-1-treated compared with anti-IgG-treated *TG*^*Alb-cre+*^ mice, with no change in ER stress markers or HCC marker AFP (Fig. 5k and Extended Data Fig. 11b–d). Multiplexed ion beam imaging (MIBI) analysis for multiparametric analysis of distinct proteins at the single-cell level^43^ in situ revealed that anti-PD-1 treatment significantly increased LDH expression, one of the crucial enzymes in aerobic glycolysis^44^, in CD8^+^ tumour-infiltrating lymphocytes (TILs) (Fig. 5l and Extended Data Fig. 11e).

In an oncogene-induced HCC model, HDTVi of *MYC:TP53*^*KO*^ caused tumour formation within 6 weeks in C57BL/6J mice, without significantly altering *Atf6* or *Hspa5* mRNA expression in tumour versus non-tumour tissue (Extended Data Fig. 11f). Anti-PD-1 monotherapy did not significantly reduce tumour burden unless *MYC:TP53*^*KO*^ mice were subjected to AAV8-mediated hepatocyte-specific ATF6α activation, which was observed 5 days after injection by HA-positive IHC (Extended Data Fig. 11g–m). Anti-PD-1 treatment of *MYC:TP53*^*KO*^*TG*^*AAV-cre*^ mice reduced the liver-to-body weight, tumour number and area (Extended Data Fig. 11l,m). CD8^+^ T cells and PD-1^+^ cells were increased after anti-PD-1 monotherapy in tumours that also displayed collagen IV loss but increased HNF4α positivity, typical of HCC (Extended Data Fig. 11n–p). Ultimately, immunotherapy prolonged the survival of *MYC:TP53*^*KO*^ mice with activated hepatocyte ATF6α (Extended Data Fig. 11q). Similar results were obtained from HDTVi of mutant *KRAS*^*G12D*^*:TP53*^*KO*^ in *TG*^*AAV-cre*^ mice (Supplementary Fig. 9a–k), in which a metabolic flux assay and scRNA-seq analysis revealed an increased glycolytic capacity in CD8^+^ TILs isolated from anti-PD-1- versus anti-IgG-treated liver tumours (Supplementary Fig. 9l–r).

**Extended Data Fig. 12:**
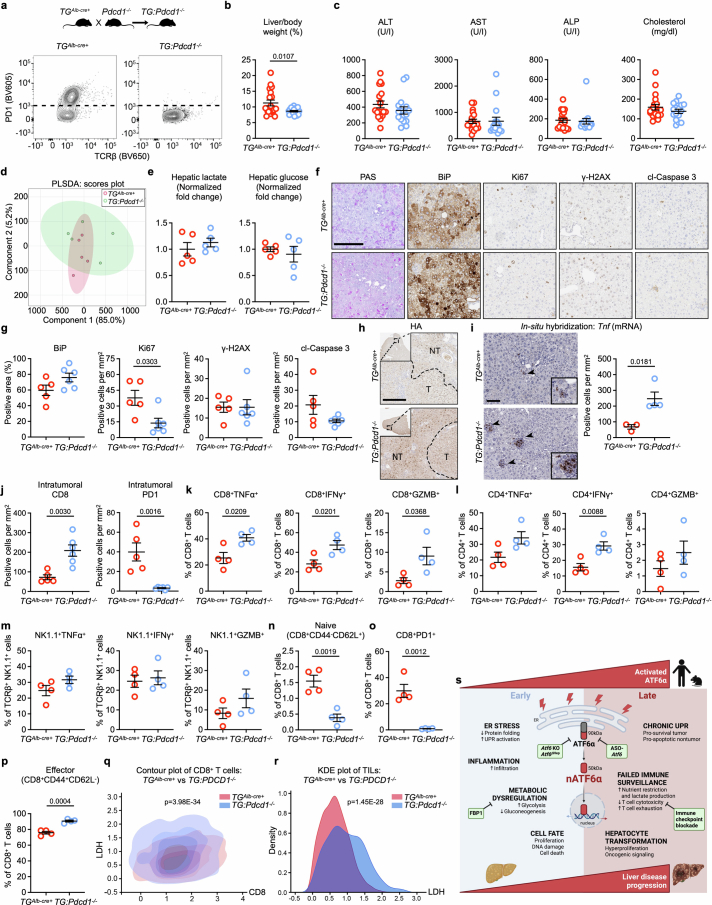
*Pdcd1* deletion enhances anti-tumour immunosurveillance in livers of *TG*^*Alb-cre+*^ mice, Related to Fig. 5. (**a**) Breeding strategy of *TG:Pdcd1*^−/−^ mice (top) and representative FACS scatter plot showing PD1-knockout efficiency (bottom). (**b**) Liver-to-body weight of 9-month-old *TG*^*Alb-cre+*^ (n = 19; 9 males, 10 females) and *TG:Pdcd1*^−/−^ mice (n = 18; 8 males, 10 females). (**c**) Serological parameters (**c**, ALT, AST, ALP and cholesterol) of 9-month-old *TG*^*Alb-cre+*^ (n = 19; 9 males, 10 females) and *TG:Pdcd1*^−/−^ mice (n = 16; 7 males, 9 females). (**d**) NMR metabolomics PLS − DA with coloured 95% confidence intervals, showing no distinct separation in metabolic profile between livers of 9-month-old *TG*^*Alb-cre+*^ and *TG:Pdcd1*^−/−^ mice (n = 5/group). (**e**) NMR-based metabolic analysis of lactate or glucose levels in livers of 9-month-old *TG*^*Alb-cre+*^ versus *TG:Pdcd1*^−/−^ mice (n = 5/group). (**f-g**) Representative PAS staining and IHC for BiP, CHOP, Ki67, γ-H2AX, and cl-Caspase 3 (**f**; scale bar = 100 µm), with quantification (**g**) in non-tumour livers from *TG*^*Alb-cre+*^ (n = 5) and *TG:Pdcd1*^−/−^ (n = 6) mice. (**h**) Representative IHC for HA (scale bar = 200 µm) in tumour (T) and non-tumour (NT) liver of *TG*^*Alb-cre+*^ and *TG:Pdcd1*^−/−^ mice. (**i**) Representative *in-situ* hybridization of *Tnf* mRNA (left; scale bar = 100 µm) and related quantification (right) in livers from *TG*^*Alb-cre+*^ (n = 3) and *TG:Pdcd1*^−/−^ (n = 4) mice. Arrowheads indicate immune cell clusters. (**j**) IHC quantification for intratumoral CD8^+^ and PD1^+^ cells in Fig. 5o from livers of *TG*^*Alb-cre+*^ (n = 5) and *TG:Pdcd1*^−/−^ (n = 6) mice. (**k-m**) Liver immune cells were isolated and stimulated (with PMA and ionomycin) for cytokine production, cytokine-secreting CD8^+^ T-cells (**k**), CD4^+^ T-cells (**l**) and NKT cells (**m**) were measured by flow cytometry in 9-month-old *TG*^*Alb-cre+*^ (n = 4) and *TG:Pdcd1*^−/−^ mice (n = 4). (**n-p**) Liver immune cells isolated from 9-month-old *TG*^*Alb-cre+*^ (n = 4) and *TG:Pdcd1*^−/−^ (n = 4) mice with naïve (**n**), CD8^+^PD1^+^ (**o**), and effector (**p**) CD8^+^ T-cell populations measured by FACS. (**q-r**) Contour plot (**q**) and KDE plot (**r**) for LDH expression in CD8^+^ TILs from livers of 9-month-old *TG*^*Alb-cre+*^ and *TG:Pdcd1*^−/−^ mice by MIBI (n = 3/group). (**s**) Schematic depiction of prolonged ATF6α activation driving early liver injury and DNA damage through cellular stress and metabolic reprogramming that dampens immunosurveillance efficacy, perpetuating oncogenic signalling for progression to HCC in mice and humans. Scatter dot plot data are presented as mean values ± SEM. Data in 12b,c,e,g,i,j-p were analysed by two-tailed unpaired *t*-test or Mann-Whitney test based on data normality distribution. Data in 12q were analysed by Mann-Whitney test. The distribution of LDH expression in 12r was visualized using overlaid KDE plots. Differences in cumulative distributions were evaluated using the Kolmogorov-Smirnov test. Mouse icons and schematic depiction were created in BioRender. Heikenwälder, M. (2026) https://BioRender.com/lgjnsy9. Source data

Finally, *TG*^*Alb-cre+*^ and *Pdcd1*^*−/−*^ mice were crossed to genetically knockout PD-1 (*TG:Pdcd1*^*−/−*^) (Fig. 5m and Extended Data Fig. 12a). PD-1 deletion resulted in elevated T cell proliferation, dampened T cell apoptosis and potentially reduced differentiation into T_reg_ cells^45^. Compared with *TG*^*Alb-cre+*^ mice, 9-month-old *TG:Pdcd1*^−/−^ mice presented significantly fewer and smaller tumours (Fig. 5n), reduced liver-to-body weight, but similar liver damage and hepatic metabolic readouts (Extended Data Fig. 12b–f). The levels of activated ATF6α (HA) and associated BiP, as well as DNA-damage-associated marker γ-H2AX, were unchanged, while Ki-67 levels were reduced and the number of TNF-producing foci was increased in the livers of *TG:Pdcd1*^−/−^ versus *TG*^*Alb-cre+*^ mice (Extended Data Fig. 12f–i).

The number of tumour-infiltrating CD8^+^ T cells in *TG:Pdcd1*^−/−^ livers increased, suggesting improved anti-tumour immunosurveillance due to *Pdcd1* deletion (Fig. 5o and Extended Data Fig. 12j). The proportion of cytokine-secreting T cells (CD8^+^ and CD4^+^ T cells, natural killer T cells) was higher in *TG:Pdcd1*^−/−^ versus *TG*^*Alb-cre+*^ livers (Extended Data Fig. 12k–m and Supplementary Fig. 10d). A greater proportion of effector T cells (CD8^+^CD44^+^CD62L^−^) and a reduced proportion of naive (CD8^+^CD44^−^CD62L^+^) as well as CD8^+^PD-1^+^ T cells, was identified in *TG:Pdcd1*^−/−^ compared with *TG*^*Alb-cre+*^ livers (Extended Data Fig. 12n–p and Supplementary Fig. 10e). MIBI revealed that CD8^+^ TILs derived from hepatic tumours of *TG:Pdcd1*^−/−^ mice displayed increased LDH expression, revealing improved CD8^+^ T cell glycolysis (Extended Data Fig. 12q,r). Ultimately, PD-1 deletion significantly improved the survival rates of *TG*^*Alb-cre+*^ mice (Fig. 5p). These data suggest that activated ATF6α sensitizes less-responsive liver cancer to immunotherapy by transforming tumours to hot or altered-immunosuppressed states^46^.

## Discussion

Here we introduce chronically activated ATF6α as a hepatic tumour driver with cell-autonomous and cell-non-autonomous functionalities, driving hepatocyte transformation and glucose-metabolism-dependent microenvironmental immunosuppression, respectively. Our data reveal the importance of post-translational ATF6α processing to derive an activated nuclear form that perpetuates ER stress, causing liver injury accompanied by inflammation, immunosuppression, metabolic dysregulation and cell fate alterations (Extended Data Fig. 12s). We acknowledge the distinction between human data focused on advanced HCC to identify molecular signatures, including ATF6α linked to disease progression and therapy response, and preclinical mouse studies examining early hepatocarcinogenesis to identify mechanisms of HCC initiation rather than reversing established disease. Preclinical mouse models (such as HFD-fed *MUP-uPA*) partly recapitulate human advanced HCC and their potential to respond to therapy, which remains a limitation of this study. Nevertheless, our findings reveal causal pathways that may inform preventive strategies and have clinical use in guiding patient stratification to optimize existing treatment approaches.

Activated ATF6α was detected in chronic hepatitis and persisted in HCC in which it further correlated with poor prognosis, involving chronic UPR, hepatocyte transformation, failed immune surveillance and immunosuppression. The notable disparity in prolonged ATF6α-mediated liver UPR observed in this study, as compared to its well-established role in short-term studies or supraphysiological acute responses^3,16^, represents a paradigm shift and underscores the distinct function of activated ATF6α in acute versus chronic diseases, as shown in the colon^15^. Either absence of or excessive UPR signalling may promote pathogenesis and impact therapeutic strategies^47^. Although activated ATF6α is a key driver, contributing roles of IRE1α and PERK UPR pathways in MASH-HCC^48^ and liver cancer cannot be excluded.

It is plausible that ATF6α-mediated UPR undergoes a transition from its adaptive function in acute diseases to a state of resistant UPR in chronic diseases with a persistent insult (for example, high caloric diet, chronic virus infection). Constitutive ATF6α activation appears to be necessary and sufficient to drive tumour development and progression, due to cell-autonomous and non-autonomous functions. We provide additional substantiation for this concept by demonstrating that ATF6α-activated hepatocytes exhibit elevated glycolysis, a reduction in crucial nutrients and an augmented release of lactate into the microenvironment that restricts T cell function. ATF6α target genes comprise chaperones from the glucose-regulated protein family, suggesting a pivotal role for ATF6α-mediated UPR in monitoring glucose fluctuations and maintaining glucose and glycosylation precursor levels within the liver. Reduced ATF6α *N*-glycosylation feeds forward toward ATF6α activation^49^. ATF6α activation depletes glycogen/glucose, further impairing glycoprotein folding to induce UPR activation and FBP1 repression; a mechanism rescued by restored FBP1 expression. Note that mice have limited liver glycogen reserves and may depend more on gluconeogenesis than humans, which maintain glucose levels by glycogenolysis^50^. Nevertheless, FBP1 repression by ATF6α is central to hepatocyte metabolism, whereby ATF6α activation and low FBP1 levels lead to HCC with aggressive phenotypes characterized by glycolysis, proliferation and immunosuppression. Consequently, this environment fosters a unique tumour immune microenvironment of T cell exhaustion, weakening natural immunosurveillance, rendering it highly responsive to ICB.

Genetic inhibition or targeting of PD-1 in mice with hepatic ATF6α activation reduced tumour burden. Accordingly, higher expression of ATF6α target genes in human liver tumours was found in complete responders to ICB monotherapy and the induction of ATF6α activation in preclinical liver cancer models significantly improved response to anti-PD-1 therapy. Therapeutically limiting hepatocyte ATF6α activation reduced cell-autonomous oncogenesis and increased naturally existing immunosurveillance. Together, ATF6α activation in HCC may serve both as (1) a promising candidate for targeted suppression in liver cancer; and (2) a potential stratification marker in human HCC, indicating increased likelihood of response to ICB therapy.

## Methods

Key reagents and resource identifiers are provided in Supplementary Table 3.

### Human samples

Human HCC TMAs used in this study were obtained with informed patient consent from K.B. as described previously^53^. In brief, TMAs with formalin-fixed paraffin-embedded (FFPE) tissues (*n* = 731) contained tumour-free/cirrhotic livers (*n* = 241), premalignant dysplastic nodules (*n* = 14) and HCCs (*n* = 473; with G1 (87), G2 (311), G3 (75))^20^. Tissue cores had a diameter of 1 mm and slides had a thickness of 1–2 µm. We complied with all relevant ethical regulations. The study was approved by the institutional ethics committee of the Medical Faculty of Heidelberg University (S-206/2005). Liver sections and snap-frozen tissue samples from healthy donors and patients with hepatitis were obtained from M.R. and N.R. with the approved institutional review board (IRB) protocol (2012-293N-MA) from the University Hospital Mannheim; from S. Roth with the approved ethical protocol S-629/2013; from A.W. with the approved application number KEK-ZH-Nr 2013-0382 by the local ethics committee (Kantonale Ethikkommission Zurich) in University Hospital Zurich. Human liver sections involved in spatial biology and IMC analysis were obtained from M. Hofmann, following the Declaration of Helsinki (1975), federal guidelines and local ethics committee regulations (Albert-Ludwigs-University, Freiburg, Germany, 20-1066). Detailed information is provided in the ‘IMC analysis of human samples’ section and Supplementary Table 2.

### Mice, diets, and treatments

The nomenclature and a description of ATF6α mouse models are provided in Supplementary Table 1. The *nATF6*^*fl/fl*^ (R26-LSL-nATF6-HA) mouse line was obtained by D. Haller^15^. *Alb-cre* mice and *Atf6*^*fl/fl*^ mice were obtained from The Jackson Laboratory. *Pdcd1*^*−/−*^ mice were provided by G. Tiegs and K. Neumann^54^. *Atf6*^−/−^ mice were described previously by R.J.K.^3^. *MUP-uPA* mice were described previously^37^. The *nATF6*^*fl/fl*^ mice were crossed with *Alb-cre* mice or intravenously injected with AAV8-cre (Vector Biolabs, VB1724 or VB1743 GFP control, 1E11VG/mouse) to generate hepatocyte-specific nATF6-HA-overexpressing heterozygous mice (*TG*^*Alb-cre+*^ or *TG*^*AAV-cre*^). Hepatocyte-specific nATF6-HA-overexpressing heterozygous mice (*TG*^*Alb-cre+*^) were bred with *Pdcd1*^−/−^ mice to generate *TG:Pdcd1*^−/−^ mice. Heterozygous R26-LSL-nATF6-HA mice (*nATF6*^*fl/+*^) were intravenously co-injected with AAV8-cre and AAV8-FBP1 or AAV8-FBP1^E98A^ (plasmids were provided by M.K. and L.G.^31^; 1E11VG/mouse) to generate hepatocyte-specific FBP1-overexpressing mice (*TG*^*AAV-cre/fbp1*^ or *TG*^*AAV-cre/fbp1E98A*^). *Atf6*^*−/−*^ and littermate *Atf6*^*+/+*^ mice were bred with *MUP-uPA* mice to generate *Atf6*^*−/−*^*:MUP-uPA* and *Atf6*^*+/+*^*:MUP-uPA* mice. The *Atf6*^*fl/fl*^ mice were crossed with *Alb-cre* mice to generate hepatocyte-specific *Atf6*-knockout mice (*Atf6*^*ΔHep*^). All of the mouse lines were either on a pure C57BL/6J genetic background or crossed into it for at least ten generations.

Mice were housed under specific-pathogen-free (SPF) conditions at the German Cancer Research Center (DKFZ) or Sanford Burnham Prebys (SBP) at constant temperature of 20–24 °C and 45–65% humidity under a 12 h–12 h light–dark cycle. All control mice were age, gender and genetic-background matched. Where applicable, littermate controls were used to minimize the variation between mouse strains.

For mice receiving injections, the following protocols were used where applicable. *TG*^*Alb-cre+*^ mice (aged 9 months) were treated with anti-PD-1 antibody (Bioxcell, BE0146) or isotype control (Bioxcell, BE0089) at an initial dose of 500 μg i.p. followed by doses of 200 μg i.p. bi-weekly for 12 weeks, as previously described^55^. Mice (aged 2 weeks) were i.p. injected once with DEN (Sigma-Aldrich, N0756, 25 mg per kg). GalNAc conjugation to ASOs against *Atf6* (GalNac-ASO-*Atf6;* Gen 2.5 ASO (16-mer 3-10-3): GAATTTTTCAGCAAGG conjugated to GalNAc on the 5′ end; Ionis Pharmaceuticals) or a scrambled nucleotide sequence (GalNac-ASO-scramble; Gen 2.5 ASO (16-mer 3-10-3): CGCCGATAAGGTACAC conjugated to GalNAc on the 5′ end; Ionis Pharmaceuticals) were subcutaneously injected at 2.5 mg per kg once weekly at 4, 9 or 30 weeks of age (see the schematics in the figures). Oncogene *NRAS*^*G12V*^ plasmid DNA (Addgene, 20205) was administered at 20 µg transposon (*NRAS*^*G12V*^) combined with 10 µg transposase (Sleeping Beauty (SB) 100, Addgene, 34879) in 2.5 ml by HDTVi per mouse at 8 weeks of age (see the schematics in the figures). The oncogene *KRAS*^*G12D*^ (5 µg per mouse, from D.T.), MYC (10 µg per mouse, from D.T.) and sg-P53 (10 µg per mouse in combination with *KRAS*^*G12D*^, 20 µg per mouse in combination with *MYC*, Addgene, 59910) plasmid DNA were delivered together with SB transposase (transposon:transposase, 5:1; from D.T.) in 2 ml saline solution through HDTVi to the mouse liver. For HDTVi experiments, mice aged 8–12 weeks were used (see the schematics in the figures).

Dietary models started after 6 weeks of age (see the schematics in the figures) and included HFD (60% HFD; BioServ F3282 or Research Diets D12492i), CD-HFD (Research Diets D05010402) and WD (Research Diets D1602230i). Cholaemic mice were excluded from dietary experiments^56^. The i.p. glucose tolerance test and insulin tolerance test were performed as previously described^57^. The pyruvate tolerance test was performed in 16-h-fasted mice by measuring the blood glucose levels after a 2 g per kg pyruvate i.p. injection. Many treatment regimens, with experimental schemes with timelines shown in the figures, extended data figures and supplementary figures, used previously published reagents and standard experimental techniques^55^.

Housing and breeding of mice without interventions were performed in accordance with the approved protocols (A-23/17, EP-Z146102, G6/22 and G279/16) in the German Cancer Research Center (DKFZ). Mouse experiments were performed in accordance with German law and the governmental bodies, with approval from the Regierungspräsidium Karlsruhe (DKFZ 332, G6/22, G11/16, G129/16, G279/16, G7/17, G80/17, G70/18, G178/19, G141/19, G132-23 and G97/24) or National Institute of Health (NIH) guidelines of the United States, with approval from the SBP Institutional Animal Care and Use Committee (IACUC, AUF 23-027 (previously 20-030), AUF 23-045 (previously 20-056)). Tumour models used in this study were orthotopic hepatic tumour models; thus, direct calliper-based measurement of tumour size in living mice was not feasible. Animal monitoring and experimental procedures strictly adhered to the termination criteria outlined in the above-mentioned protocols (DKFZ 332, G6/22, G11/16, G129/16, G279/16, G7/17, G80/17, G70/18, G178/19, G141/19, G132-23 and G97/24 in DKFZ; IACUC, AUF 23-027 and AUF 23-045 in SBP). Each mouse was examined daily by trained animal care staff or research personnel. Animals exhibiting signs of distress, morbidity, clinical signs of pain or distress (including but not limited to cachexia, cyanosis, dyspnoea, ascites, or lack of mobility, food and water intake), or any abnormality meeting the predefined termination criteria were promptly euthanized, after which the biological materials were collected. These limits were not exceeded in any of the experiments. Mice that remained clinically normal and did not reach the termination criteria were maintained until the designated experimental endpoint, at which time they were euthanized, and the liver tumours were excised and measured.

### Measurements of serum parameters

Blood was drawn by cardiac puncture after dissection, and centrifugation was used to isolate serum using serum isolation gel tubes (Sarstedt, Z/1.1). The serology parameters were measured with commercially available FUJIFILM DRI-CHEM slides for ALT, AST, TCHO, TBIL, ALB and ALP on FUJIFILM DRI-CHEM NX500i or with Vetscan Mammalian Liver Profile rotors (Abaxis, 500-0040-12) with Vetscan VS2 Chemistry Analyzer. Fasting insulin levels were measured with 10 µl of serum from 16 h fasted mice by ELISA, according to the manufacturer’s guidelines (Mercodia, 10-1247-01) Serum lipids were measured using Infinity Reagents (Thermo Fisher Scientific, TR22421 triglycerides, TR13421 cholesterol).

### Cell lines and culture conditions

Cancer cell lines and viral studies were approved by the Institutional Biosafety Committees. The FL83B cells were purchased from ATCC. Colo800 and MART-I T cells were obtained from R.C.^58^. The HLE cells originated from Japanese Collection of Research Bioresources Cell Bank (JCRB)^59^. The generation of nATF6-overexpression and *Atf6* knock-out (KO) cell lines was done in collaboration with J.K.

To generate nATF6-overexpression cells, the coding sequence encoding the activated form of mouse *Atf6* (*nAtf6*, amino acids 1–373) or human *ATF6* (nATF6, amino acids 1–386) and HA tag was cloned between the XhoI and EcoRI restriction sites of the retroviral plasmid MSCV-linker-IRES-GFP, resulting in the MSCV-nAtf6-IRES-GFP or MSCV-nATF6-IRES-GFP vector, respectively. nATF6 overexpressing (FL83B^TG^, HLE^TG^ and Colo800^TG^) and control (FL83B^WT^, HLE^WT^ and Colo800^WT^) cells were prepared by transduction of cells with retroviral particles containing MSCV-nAtf6-IRES-GFP or MSCV-nATF6-IRES-GFP and MSCV-linker-IRES-GFP construct, respectively. Viral particles were produced in Phoenix GP cells (ATCC CRL-3215) after transfection with either MSCV-nAtf6-IRES-GFP, or MSCV-nATF6-IRES-GFP or MSCV-linker-IRES-GFP vector together with VSV-G (Clontech) vector. Cells were expanded and sorted for GFP using the FACS Aria II (BD) system.

The FL83B *Atf6* KO cells were prepared by transfection (Lipofectamine 3000 Transfection Reagent, Thermo Fisher Scientific) of FL83B cells with vectors derived from pSpCas9(BB)−2A-Puro (PX459) V2.0^60^ and following selection with puromycin (10 μg ml^−1^) for 3 days. The sequences for single guide RNAs (sgRNAs) and primers for verification of indel formation were designed using the CRISPOR.org webtool^61^. Control cells for FL83B *Atf6*-KO cells were transfected with PX459 V2.0 without any sgRNA cloned in. Indel formation was verified by TIDE assay^62^.

Cells were cultured in F12K Nut mix (FL83B cells, Invitrogen) or RPMI 1640 GlutaMax (Colo800 cells, HLE cells and MART-I T cell, Invitrogen) containing 10% FBS (Invitrogen) and 1% penicillin–streptomycin (GIBCO).

### In vitro T cell killing assays

Colo800^WT^ and Colo800^TG^ cells were cultured overnight in 96-well ePlates (OMNI Life Science), followed by co-culture with or without MART-I T cells in a ratio 1:5 for 3 days. The tumour cell growth rate was measured using the Agilent xCELLigence platform. For the rescue experiment, galloflavin (AOB1024-10, 200 μΜ) or AZD3965 (S7339, 1.6 nM) were used. The same protocol was applied to HLE^WT^ and HLE^TG^ cells but MelanA peptide was included at 25 ng ml^−1^ in the culture medium to ensure proper antigen presentation.

### Metabolic flux analysis using the Seahorse bioanalyser

For extracellular acidification rate determination, the Agilent Seahorse XF Glycolysis Stress Test kit (Agilent, 103020-100) on the Seahorse Agilent XF96 platform was used according to the manufacturer’s instructions. On Cell-Tak-coated (Corning, 354240) plates, 20k FL83B^WT^/FL83B^TG^ cells or 100,000 MACS-purified primary TILs were seeded and crystal violet staining was performed after the assay for cell number normalization. The results were calculated with Agilent Wave Software v.2.6. At least three biological replicates, averaging up to eight technical replicates each, were used per experiment in quantifications.

### TEM analysis

TEM was performed in collaboration with M.P. Liver tissues were fixed with 2% paraformaldehyde and 2.5% of glutaraldehyde in 0.15 M sodium cacodylate buffer (SC buffer pH 7.4) for 48 h at 4 °C. The samples were placed in 1% osmium tetroxide in 0.15 M sodium cacodylate for 1–2 h on ice. The samples were washed five times for 10min in 0.15 M SC buffer followed by rinsing in double-distilled H_2_O on ice and incubated in 2% of uranyl acetate for 1–2 h at 4 °C. The samples were dehydrated in ethanol: 50%, 70%, 90%, twice at 100% for 10 min each on ice followed by dry acetone for 15 min at room temperature. Samples were incubated in 50:50 ETOH: Durcupan for at least 1 h at room temperature, followed by 100% Durcupan overnight. The next day, the samples were placed in fresh 100% Durcupan for half a day at room temperature. Tissues were embedded in Durcupan at 60 °C in an oven for 36–48 h. Ultrathin sections (60 nm) were cut on Leica microtome with Diamond knife followed by post-staining with both uranyl acetate and lead. Images were captured on JEOL 1400 plus TEM at 80KV with Gatan 4kx4k camera.

### Immune cell isolation and FACS

The isolation and staining of lymphocytes for flow cytometry followed a protocol described previously^55^. The mice were euthanized and the livers perfused with 0.9% NaCl buffer. Livers/tumours were collected, minced, digested with collagenase and DNase, and subsequently passed through a 100-μm filter. Hepatic lymphocytes were then purified by a two-step Percoll gradient. The spleens were passed through 100-μm mesh and washed to isolate splenic lymphocytes. The samples were treated with red blood cell lysis buffer for 5 min at room temperature, followed by a wash step. Magnetic-activated cell sorting (MACS)-based positive/negative selection (Miltenyi Biotec 130-090-101, Dead Cell Removal Kit; Miltenyi Biotec 130-117-044, CD8a (Ly-2) MicroBeads) was used to purify live TILs according to the manufacturer’s instructions.

For lymphocyte stimulation, cells were cultured in RPMI 1640 supplemented with 2% (v/v) FBS. Cell activation cocktail with brefeldin A (BioLegend, 423304) and monensin solution (BioLegend, 420701) were diluted in the medium at 1:500 and 1:1,000, respectively. Antibody staining was done in the presence of Fc receptor blockade in FACS buffer. For live/dead cell discrimination, the ZombieDyeNIR was used according to the manufacturer’s guidelines. After washing with FACS buffer and centrifugation (400*g*, 5 min, 4 °C), the cells were stained for 40 min at 4 °C with 25 μl of titrated antibody master mix and washed. Where applicable, the samples were sorted by FACS. eBioscience intracellular fixation buffer (00-8222-49) was used to fix samples for flow cytometry according to the manufacturer’s guidelines. For samples requiring intracellular staining, eBioscience Perm buffer (00-8333-56) was used. The BD FACS Fortessa system was used to analyse the stained cells, and FlowJo was used to analyse data. In collaboration with the DKFZ FACS core facility, a FACS Aria II machine and a FACS Aria FUSION machine were used for sorting.

### Histological staining and in situ hybridization

The histology, IHC and scanning were performed as described previously^57^. Mice were euthanized and tissues were cryo-preserved or collected and fixed in 4% paraformaldehyde for 24 h. Paraformaldehyde-fixed tissues were paraffin-embedded, cut and stained in collaboration with the technical team from the Department of Chronic Inflammation and Cancer, DKFZ, Heidelberg or SBP Histology Core, San Diego.

For histological staining, FFPE tissues were cut to prepare 2-μm sections. These sections were stained with H&E or IHC with the antibodies listed in Supplementary Information on the Bond-MAX machine (Leica). The ATF6α IHC score in human liver was evaluated by a certificated physician^53^ based on intensity: 1, low/not detected; 2, moderate; 3, high; cytoplasm: 0, negative; 1, ≤33%, 2, 34–66%, 3, ≥67%; nuclear: 0, negative, 1, 1%; 2, 1–5%; 3, 6–20%; 4, ≥21%. For lipid droplet staining, 5-μm sections from cryo-preserved tissues were stained with Sudan Red (0.25% Sudan IV in ethanolic solution) or Oil Red O (0.5%, PolyScientific, K043). In situ hybridization was performed according to the manufacturer’s instructions, the probe and reagents were purchased from Advanced Cell Diagnostics (ACD). 5-µm sections from mouse FFPE tissue were used. All stained slides were scanned with the Aperio AT2 DX System (Leica) and analysed by macro-based analysis by ImageJ (1.54g) or QuPath (v.0.5.1).

### Immunofluorescence

Immunofluorescence microscopy was performed in collaboration with D. Heide and J. Hetzer. In brief, mouse liver tissue was embedded in optimal cutting temperature (OCT) compound. Then, 25-μm liver sections were permeabilized and blocked with 0.3% Triton X-100 (Sigma-Aldrich) and 10% FBS in PBS. CD3 (Invitrogen, MA1-90582), CD8 (BD, 553027) and PD-1 (R&D, AF1021) primary antibodies were used to stain the samples. Stained slides were covered with fluorescence mounting medium (DAKO) and scanned with the NanoZoomer S60 Digital slide scanner (Hamamatsu Photonics).

### NMR spectroscopy-based metabolomics

Nuclear magnetic resonance (NMR) spectroscopy-based metabolomics analysis was performed in collaboration with L.Z., D.B. and C.T. at the Werner Siemens Imaging Center (WSIC), Eberhard Karls University of Tübingen. In brief, liver pieces were cryogenically pulverized (Covaris cryoPREP CP02) and the powder was transferred to 2 ml adaptive focused acoustics glass tubes, where it was suspended in 300 μl ultrapure methanol, 1000 μl *tert*-butylmethyl ether and subjected to ultra-sonication-based 2-phase metabolite extraction procedures (Covaris E220 Evolution) using two consecutive sonication programs with vertical sample movement for maximum extraction yield. After ultrasonication, 250 μl of molecular-biology-purity-grade water was added. The samples were centrifuged for phase separation at 12,000*g* for 10 min. The layers were then manually separated, aqueous phase was transferred to 1.5 ml tubes and evaporated overnight in a vacuum concentrator (Speedvac SPD300, Thermo Fisher Scientific).

Similarly, cell culture pellets collected in 1.2 ml ultrapure methanol were transferred to 2 ml adaptive focused acoustics glass tubes and subjected to a one-phase extraction procedure. Then, 110 μl of chloroform and ultrapure water were added, and the mixtures were subjected to ultrasonication extraction. The mixtures were centrifuged to remove any potential solid particles, transferred to a clear glass vial and evaporated to dryness in the vacuum concentrator.

Dried metabolite pellets both from cell culture and liver tissue extracts were resuspended in deuterated phosphate buffer (pH 7.4, 1 M K_2_HPO_4_, NaN_3_ containing 1 mM internal reference standard 3-(trimethylsilyl) propionic-2,2,3,3-d_4_ acid sodium salt (TSP)) for quantification. The mixtures were again centrifuged at 30,000*g* for 30 min to separate undissolved substances. Clear supernatant was filled into 1.7 mm Bruker SampleJet-compatible NMR spectroscopy tubes. We acquired NMR spectra on a 600 MHz (proton frequency) spectrometer (Avance III HD, Bruker BioSpin) with a 1.7 mm room temperature microprobe at 298 K. A short proton ZG (zero go) experiment was recorded followed by a 7 min 1D NOESY (nuclear Overhauser effect) to assess offset the frequency and optimize water suppression. Carr–Purcell–Meiboom–Gill experiments were used for each polar extract sample to assign and quantify metabolites by suppressing residual background signals from remaining water and macromolecules (512 scans, 1 h for liver samples; 1,024 scans, 2 h for cell culture).

Spectra were preprocessed with Bruker TopSpin v.3.6.1, and annotated and quantified using ChenomX NMR suite v.8.5. The statistics were performed by MetaboAnalyst 5.0 online platform (www.metaboanalyst.ca). In brief, we normalized the dataset by reference sample using probabilistic quotient normalization and performed a parametric analysis of variance (ANOVA) with an adjusted *P*-value (FDR) cut-off of 0.05 with Fisher’s LSD post hoc analysis. For the heat maps, we used the Euclidean distance measure with Ward clustering algorithm.

### ^13^C-lactate labelling and GC–MS

*TG*^*AAV-gfp*^*, TG*^*AAV-cre*^ and *TG*^*AAV-cre/fbp1*^ mice at 13 weeks of age were fasted for 16 h overnight. The mice were injected intravenously with 0.25 mg per g sodium L-lactate (^13^C_3_) three times at 15 min intervals before euthanasia. Liver was collected and immediately snap-frozen in liquid nitrogen for metabolomic analysis.

The sample extraction methods were as follows: frozen liver samples (25–50 mg) were transferred to 2 ml tubes containing 2.8 mm ceramic beads (Omni International) and 0.45 ml ice-cold 50% methanol/20 µM L-norvaline was added. The tubes were shaken (setting 5.5) for 30 s on the Bead Ruptor 12 (Omni International) system, quickly placed onto ice and frozen at −80 °C overnight. Thawed samples were centrifuged at 15,000*g* for 10 min at 4 °C. The supernatant was then transferred to a new tube, mixed with 0.225 ml chloroform and centrifuged at 10,000*g* for 10 min at 4 °C. This produced a two-phase separation. Portions (100 µl) of the top phase were dried (Speedvac) for analyses of polar metabolites.

Metabolite derivatization and GC–MS run conditions: polar metabolites except for sugar phosphates were derivatized using isobutylhydroxylamine and MTBSTFA and analysed by GC–MS for ^13^C labelling and metabolite quantities as described previously^63^.

The samples were transferred to autosampler vials with inserts and analysed using the Rxi-5ms column (15 m × 0.25 mm inner diameter × 0.25 μm, Restek) installed in a Shimadzu QP-2010 Plus gas chromatograph–mass spectrometer (GC–MS). The GC–MS was programmed with an injection temperature of 250 °C, 1 µl injection volume and split ratio 1/10. The GC oven temperature was initially 130 °C for 4 min, rising to 230 °C at 6 °C min^−1^, and to 280 °C at 60 °C min^−1^ with a final hold at this temperature for 2 min. The GC flow rate, with helium as the carrier gas, was 50 cm s^−1^. The GC–MS interface temperature was 300 °C and (electron impact) ion source temperature was 200 °C, with 70 eV ionization voltage. The main glucose peak eluted at 13.7 min, and fragments of *m*/*z* 319 (contains 4 glucose carbons; overall formula C_13_H_31_O_3_Si_3_) and *m*/*z* 205 (contains 2 glucose carbons; overall formula C_8_H_21_O_2_Si_2_) were used to analyse ^13^C-glucose labelling as described previously^63^.

Samples for sugar-phosphate analysis were derivatized first with 30 µl ethylhydroxylamine (Sigma-Aldrich) 20 mg ml^−1^ in pyridine for 20 min at 80 °C, and secondarily with 30 µl BSTFA (Thermo Fisher Scientific) for 60 min at 80 °C. The samples were transferred to autosampler vials with inserts and analysed using an Rxi-5ms column (15 m × 0.25 mm inner diameter × 0.25 μm, Restek) installed in a Shimadzu QP-2010 Plus GC–MS system. The GC–MS was programmed with an injection temperature of 250 °C, 1.6 µl injection volume and split ratio 1/10. The GC oven temperature was initially 85 °C for 4 min, rising to 115 °C at 8 °C min^−1^, to 210 °C at 20 °C min^−1^ and to 280 °C at 6 °C min^−1^ with a final hold at this temperature for 2 min. The GC flow rate, with helium as the carrier gas, was 50 cm s^−1^. The GC–MS interface temperature was 300 °C and (electron impact) the ion source temperature was 200 °C, with 70 eV ionization voltage. Norvaline (internal standard) eluted at 6.7 min, glucose-1-phosphate at 15.7 min, the main fructose-6-phosphate peak at 16.1 min, and the main glucose-6-phosphate peak at 16.3 min. Fructose-6-phosphate was analysed for ^13^C-labelling using the fragment of *m*/*z* 459 containing three fructose carbons; the overall formula C_15_H_40_O_6_Si_4_P. Glucose-6-phosphate was analysed using the *m*/*z* 357 fragment (C_11_H_30_O_5_Si_3_P) containing two glucose carbons and the *m*/*z* 471 fragment (C_16_H_40_O_6_Si_4_P) containing four glucose carbons. Glucose-1-phosphate and glucose-6-phosphate were both quantified using *m*/*z* 315 and *m*/*z* 387 fragments; *m*/*z* 315 and 459 were used for quantifying fructose-6-phosphate, and the amounts were corrected for recovery of norvaline (*m*/*z* 144).

### Metabolomic analysis of liver tissue by LC–MS/MS

Metabolomic analysis of liver tissue by LC–MS/MS was performed in collaboration with L.M., N.M., S. Meckelmann and A.T. at University Hospital Essen and German Cancer Consortium, Essen. For metabolomic profiling using LC–MS/MS, 30–50 mg liver tissue was homogenized in ice-cold methanol using an electronic tissue disruptor (Qiagen). Metabolites were extracted following a two-step liquid method adapted from a previous study^64^ with the addition of internal standards (^13^C_6_-L-Arginine, ^13^C_5_-L-Valine, ^13^C_2_-citric acid, ^2^H_4_-succinic acid and ^13^C_6_-Fructose-6-phosphate). After homogenization, sonication and centrifugation, the supernatant was collected, dried and reconstituted.

Chromatography separation was performed on the Agilent 1290 Infinity II Bio LC system using the AdvanceBio MS Spent Media column (150 mm × 2.1 mm, 2.7 μm). A gradient elution was applied at 450 μl min^−1^ with solvent A (10 mM ammonium acetate in water, pH 9) and solvent B (acetonitrile/water, 95:5, with 10 mM ammonium acetate). The gradient progressed from 100% B at 0 min, to 50% B at 6.5 min, reverting to 100% B at 7.01 min, with 1.1 min equilibration between runs. The column temperature was maintained at 70 °C, and 1 μl of the sample was injected.

MS was conducted using a Thermo Orbitrap Q Exactive Plus, equipped with a HESI II ion source operating in both positive and negative modes. Full scans (70–1,050 *m*/*z*) were acquired at 35,000 resolution, while MS2 spectra were obtained at 17,500 resolution. Data analysis was performed using MS-Dial 4.9.2, enabling compound identification based on accurate mass and MS2 spectra, supported by an in-house retention time library.

### PET/CT and MRI

The positron emission tomography–computed tomography (PET-CT) and magnetic resonance imaging (MRI) were done in collaboration with J.M. from the core facility of DKFZ. The PET/CT examinations were carried out on a special small animal scanner (Inveon PET/SPECT/CT, Siemens). The mice were injected with the F18 radioactively labelled tracer FDG through a tail catheter. The maximum injection quantity was 100 μl and total activity applied between 3–8 MBq. Shortly before the examination, the animals were fasted for 4 h to ensure targeted absorption of the tracer into the tumours. The mice were anaesthetized (inhalation anaesthesia with sevoflurane (3–3.5% by volume) and air (0.5 l min^−1^)) before 0.1 ml of contrast medium (Prohance (Gadoteridol, Bracco), 0.5 mmol kg^−1^ bodyweight, Bayer Schering Pharma) was i.p. injected. MRI examinations were carried out on a preclinical 1 T small-animal tomograph (ICON, Bruker).

### Protein extraction, western blotting and proteomics

The protocols for protein isolation and western blot analysis were described previously^57^. In brief, livers were homogenized (Fisherbrand Bead Mill 24, 15340163) and homogenate or cell lysis was prepared in RIPA buffer (Cell Signaling Technology 9806S) or T-PER buffer (Thermo Fisher Scientific, 78510), with protease and phosphatase inhibitor cocktail (Thermo Fisher Scientific, 78440). The lysates were centrifuged at 10,000*g* for 10 min at 4 °C. The protein concentration was quantified by Pierce BCA protein assay (Thermo Fisher Scientific, 23225). A total of 30–50 µg of protein was denatured at 95 °C for 5 min in Laemmli buffer containing 5% β-mercaptoethanol and loaded in SDS gel for electrophoresis. Protein was then deposited onto PVDF membranes (Immobilon-P, Merck Millipore) or nitrocellulose membranes (Bio-Rad, 1704159) by semi-dry electroblotting (Trans-Blot Turbo Transfer, Bio-Rad, 1704150). The membranes were further incubated in 5% BSA or 5% skimmed milk solution for 1 h to overnight before primary antibody incubation. The primary antibodies listed in the Supplementary Information were incubated overnight on a shaker at 4 °C. After three 10 min washes with PBST or TBST, secondary antibodies were incubated with antibody solution for 1–2 h. Detection was accomplished using Clarity Western ECL Substrate (Bio-Rad) in conjunction with the ChemiDoc Touch imaging equipment (Bio-Rad). For IRDye (Licor) secondary antibody incubation protected from light, fluorescence was detected by Odyssey imaging system (Licor) combined with the acquisition software Image-Studio (Licor). Quantification of bands of interest in the linear range of exposure was performed by densitometry using ImageJ. Uncropped scans of blots are provided in the Supplementary Information. For any quantitative comparisons between samples or proteins on different gels/blots, the samples were derived from the same experiment and the gels/blots were processed in parallel. Where applicable, consistent loading of proteins was further validated by Ponceau S staining solution (Thermo Fisher Scientific).

For MS, an equivalent amount of protein from mouse liver tissue was submitted to the proteomics core facility of the DKFZ and the protocols were described previously^55^. The raw intensity proteomics data were analysed using Maxquant^65^ (v.2.4.3) with the default settings. Specifically, the UniProt *Mus musculus* reference proteome (UP000000589) was used as a reference for protein identification and quantification. The additional parameters were used run MaxQuant as follows: trypsin was used as enzyme digestion allowing two missed cleavages, caramidomethyl was used for the fixed modification while the variable modifications were set to acetyl and oxidation, and the mass tolerances for the first and the main search were set to 20 and 4.5, respectively. Contamination proteins were removed from the identified proteins using the common contamination database. The normalized spectral protein intensity (LFQ) was used to calculate the protein abundance. Additional differential protein abundance was analysed using Persues R package using Welch’s *t*-test and FDR-corrected *P* values. The proteomics data described in this article are available at the ProteomeXchange Consortium (Supplementary Information).

### RNA extraction, RT–qPCR and RNA-seq

According to the manufacturer’s protocol, total RNA isolation from snap-frozen liver tissue or cultured cells was performed using the RNeasy Mini Kit (Qiagen, 74106). The on-column DNA digestion was carried out using an RNase-free DNase kit (Qiagen) or RNA samples were treated with TURBO DNase (Thermo Fisher Scientific, AM1907) according to the manufacturer’s protocol to completely remove genomic DNA. RNA concentration and quality were determined by Nanodrop (Thermo Fisher Scientific) for quantitative PCR with reverse transcription (RT–qPCR) and by Qubit for RNA-seq. Then, 1 µg of RNA was reverse-transcribed using High-Capacity cDNA Reverse Transcription Kit with RNase Inhibitor (Thermo Fisher Scientific, 4374967) in a final volume of 20 μl according to the manufacturer’s instructions before RT–qPCR. In a 384-well plate, RT–qPCR was performed in duplicate using Fast Start SYBR Green Master Rox (Roche) or triplicate with iTaq Universal SYBR Green Supermix (Bio-Rad, 1725124). Eurofins, Millipore-Sigma or Integrated DNA Technologies (IDT) supplied custom-made primers using a 7900 HT RT-qPCR equipment (Applied Biosystems, Life Technologies) or CFX384 Real-time PCR system (Bio-Rad). RNA-seq was performed in collaboration with Genomics & Proteomics Core Facility in DKFZ or Genomics Core Facility after ScreenTape (Agilent) RNA quality control validation in SBP. The methodology is described in brief below and the accession numbers are listed in the Supplementary Information.

At the SBP Genomics Core, the RNA-seq assay was performed using the Illumina NextSeq 500 platform. In brief, poly(A) RNA was isolated using the NEBNext poly(A) mRNA magnetic isolation module and barcoded libraries were made using the NEBNext Ultra II Directional RNA Library Prep Kit for Illumina (NEB). Libraries were pooled and single-end sequenced (1 × 75) on the Illumina NextSeq 500 using the High output V2 kit (Illumina). Raw reads were preprocessed by trimming Illumina Truseq adapters, poly(A) and poly(T) sequences using cutadapt (v.2.3)^66^ with the parameters ‘cutadapt -j 4 -m 20 --interleaved -a AGATCGGAAGAGCACACGTCTGAACTCCAGTCAC -A AGATCGGAAGAGCGTCGTGTAGGG AAAGAGTGT Fastq1 Fastq2 | cutadapt --interleaved -j 4 -m 20 -a “A{100}” -A “A{100}” - | cutadapt -j 4 -m 20 -a “T{100}” -A “T{100}” -’. Trimmed reads were subsequently aligned to the mouse genome version mm10 using STAR aligner (v.2.7.0d_0221)^67^ with parameters according to ENCODE long RNA-seq pipeline (https://github.com/ENCODE-DCC/long-rna-seq-pipeline). Gene expression levels were quantified using RSEM (v.1.3.1)^68^. Ensembl v84 gene annotations were used for the alignment and quantification steps. RNA-seq sequence, alignment and quantification qualities were assessed using FastQC (v.0.11.5) and MultiQC (v.1.8)^69^. Low-expressed genes were filtered out by retaining genes with estimated counts (from RSEM) ≥ number of samples times five. Filtered estimated read counts from RSEM were used for differential expression comparisons using the Wald test implemented in the R Bioconductor package DESeq2 v.1.22.2 based on generalized linear model and negative binomial distribution^70^. Genes with Benjamini–Hochberg corrected *P* < 0.05 and fold change ≥2.0 or ≤2.0 were selected as differentially expressed genes. Pathway analyses of differential expression comparisons were performed using IPA (Qiagen).

At the Genomics & Proteomics Core Facility in DKFZ, primary analysis of bulk RNA-seq data were performed using the nextflow pipeline nf-core/rnaseq (v.3.8) for the primary analysis of bulk RNA-seq data. Specifically, FastQC (v.0.11.9; https://www.bioinformatics.babraham.ac.uk/projects/fastqc) was used for quality control of the FastQ data followed by adapter trimming using Trim Galore (v.0.6.5) (https://github.com/FelixKrueger/TrimGalore). Reads were aligned to the mouse reference genome GRcm38.86 using STAR alinger^67^ (v.2.7.10) and the subsequent read mapped to the genes were counted using featureCount module implemented in the subread R package^71^ (v.1.6.4). Further quality control was performed using dupRader^72^ and RSeQC^73^ (v.2.6.4). Downstream analysis of the read counts matrix, including normalization (rlog and vst) and differential gene expression analysis was performed using DEseq2 R package^70^ (v.1.40.2). A log-transformed fold change of 1 and FDR-corrected *P* value of 0.05 was used as a cut-off to identify differentially expressed genes. Sample distances were calculated using dist R (v.3.8) function using the entire gene expression. Pathway analysis of differentially expressed genes was carried out using the gProfiler2 R^74^ (v.0.2.0) package.

### Gene signatures and database analyses

Two gene set signatures were used in this Article to represent ATF6α activation:

(1) The human ATF6α-activation signature was derived from the MSigDB (www.msigdb.org)^17^ human gene set: ATF6_TARGET_GENES, including 1,081 ATF6α transcription factor targets^75^.

To assess the human ATF6α-activation signature in HCC tissue and non-tumour liver samples from patients with HCC shown in Fig. 1a, mRNA expression datasets were obtained from online repositories including the Gene Expression Omnibus (GEO), The Cancer Genome Atlas (TCGA) and the International Cancer Genome Consortium data portal. For datasets produced using Affymetrix whole-genome microarrays, raw .CEL files were processed using the SCAN (single-channel array normalization) method^76^ and the SCAN.UPC R/Bioconductor package, and samples with median GNUSE quality scores^77^ above 1.25 (computed with the frma Bioconductor package) were flagged for exclusion. For all of the other datasets, processed data provided by the original authors were used. Cases of fibrolamellar carcinoma were removed^78^. For all datasets, probe or gene IDs were mapped to Entrez gene identifiers. For microarray platforms containing multiple alternative probes or probesets per gene, the probe showing the great variance in expression among HCC samples was selected. The human ATF6α-activation signature was used to quantify relative gene set enrichment scores across HCC and non-tumour samples in each dataset using the GSVA (gene set variation analysis) Bioconductor package^79^. Meta-analyses across datasets were computed with the metafor R package^80^ using a random-effects model and the DerSimonian–Laird estimator. The same datasets were used to assess mRNA expression of *ATF6* and *FBP1* in HCC tissue and non-tumour liver samples from patients with HCC shown in Supplementary Fig. 1e and Extended Data Fig. 6b. To evaluate the correlation between the expression levels of genes and gene signatures in human HCC shown in Extended Data Fig. 6a, 15 datasets with extensive whole-genome RNA expression data were chosen (TCGA-LIHC^81^: GSE65485, GSE50579, GSE45436, GSE62232, GSE9843; iCOD^82^: GSE63898, GSE64041, GSE76297, GSE16757), and gene expression values were batch-adjusted using the ComBat method^83^ as implemented by the sva R package. The Pearson correlation coefficient was computed, as well as the odds ratio and *P* value based on a cut-off of +1 s.d. from the mean using Fisher’s exact test.

To analyse the role of ATF6α in determining response to anti-PD-1 monotherapy in patients with HCC, we used a cohort of 83 patients^25^. In Fig. 1r, mRNA expression levels of ATF6α-related genes were plotted for ATF6α^hi^ and ATF6α^low^ samples with enrichment scores for the human ATF6α-activation signature generated by ssGSEA (see below).

Signature profiles shown in Extended Data Fig. 1a–c were assessed in two cohorts of patients with HCC (*n* = 171 (ref. ^19^) and *n* = 228^18,84^), with samples distributed by high to low enrichment of the human ATF6α-activation signature. For Extended Data Fig. 1a,b, the following signatures were used: proliferation subclass^85^; S2 subclass^86^; EPCAM^87^; CK19_1 (ref. ^18^); CK19_2 (ref. ^88^); Notch^18^; Vascular Invasion^89^; MET^90^; mTOR signalling^91^; IGF signalling^92^; TGFβ late^93^.

For Kaplan–Meier plots, the median of the human-derived ATF6α-activation signature described above (Fig. 1b) or MSigDB REACTOME_UNFOLDED_PROTEIN_RESPONSE_UPR (Fig. 1c) was used to divide TCGA-LIHC samples by median split into high and low groups and produce Kaplan–Meier plots, which visualize survival probability over time^94^. The same ssGSEA method applied to Supplementary Fig. 1g where TCGA BLCA, COAD-READ, BRCA, GBM, LUAD and SKCM samples were divided by the median split of human ATF6α-activation signature and visualized for survival probability over time. Finally, TCGA-LIHC samples were divided by the median split into high and low groups according to the expression of UPR-related gene mRNA or MSigDB Human Gene Sets (ATF4_Q2; CHOP_01; XBP1_01). In Fig. 1b,c and Supplementary Fig. 1g, patients who had events recorded after 60 months (180 days) were treated as alive at the 60-month timepoint. Statistical significance for differences between the high and low enrichment groups was assessed using the Mantel–Cox log-rank test. The Kaplan–Meier survival analysis was performed in the Python programming language using the lifelines package^95^ and visualized using matplotlib (10.5281/zenodo.592536). The TCGA expression data were from TCGA PANCAN data freeze 1.1 (19 August 2015).

(2) The mouse ATF6α-activation signature and ssGSEA enrichment scores generation were generated using mouse-derived differentially expressed genes in liver tissue of DEN/HFD-treated *TG*^*AAV-cre*^ versus *TG*^*AAV-gfp*^ mice using RNA-seq. Statistically significant genes (Abs(log_2_[FC]) ≥ 1 BHP < 0.05) included 888 upregulated and 266 downregulated genes to define the up and down gene sets representing mouse-derived ATF6α-activation in liver. ssGSEA was used to provide ATF6α-activation ssGSEA_UP and ssGSEA_DN enrichment scores for each of the TCGA-LIHC samples. Each ssGSEA enrichment score represents the degree to which the genes in a particular gene set are coordinately upregulated or downregulated within a sample. In this manner, ssGSEA projects a single sample’s gene expression profile from the space of single genes onto the space of gene sets. The UP and DN enrichment scores are then combined into one signature by subtracting the DN score from the UP score: ssGSEA_combined = ssGSEA_UP − ssGSEA_DN. For more details on ssGSEA, see the original references^27,96^ and the documentation of the single-sample GSEA module in GenePattern (www.genepattern.org/modules/docs/ssGSEAProjection/4/). To quantify the degree of association of single gene mRNA and gene set ssGSEA profiles, we used the IC^97^ a mutual information measure of correlation similar to the Pearson correlation coefficient, but better for detecting non-linear associations. An empirical permutation test was used to assess statistical significance and compute *P* values and false-discovery rates (FDR). The ssGSEA_combined scores represent the activation of the mouse-derived ATF6α-activation signature and are shown at the top of Fig. 3q, correlated with human gene sets from the MSigDB (www.msigdb.org)^17^:

ER stress: ATF6_TARGET_GENES (same as the ATF6α-activation signature used above); REACTOME_UNFOLDED_PROTEIN_RESPONSE_UPR; DDIT3 (mRNA); REACTOME_ASPARAGINE_N_LINKED_GLYCOSYLATION.

Metabolism: FBP1 (mRNA); Q1_HYPOXIA_TARGETS_OF_HIF1A_AND_FOXA2; HALLMARK_OXIDATIVE_PHOSPHORYLATION.

Oncogenesis: NRF2_01; ROS_AND_RNS_PRODUCTION_IN_PHAGOCYTES; HALLMARK_PI3K_AKT_MTOR_SIGNALING; REACTOME_SIGNALING_BY_WNT; REACTOME_SIGNALING_BY_TGFB_FAMILY_MEMBERS; LEE_LIVER_CANCER_MYC_E2F_UP.

Immunosuppression: CTLA4; PD-1; GSE26495_PD1HIGH_VS_PD1LOW_CD8_TCELL_UP; GSE9650_EFFECTOR_VS_EXHAUSTED_CD8_TCELL_UP; GSE9650_EFFECTOR_VS_EXHAUSTED_CD8_TCELL_DN.

For the heat map of immune features and signatures related to ICB response in HCC (Extended Data Fig. 9b), human patient tumour samples (*n* = 171)^19^ were divided into two groups by high and low enrichment of the mouse-derived ATF6α-activation signature. Human patient tumour samples were defined as high or low using the nearest template prediction (NTP) module from Gene Pattern^98^ and the mouse-ATF6α-activation signature described above. Positivity for the immune, IFNAP and poor prognosis signatures (Figs. 1r and 5a and Extended Data Fig. 9b) was similarly defined by NTP module from Gene Pattern^98^ as previous described^25^, and a significant prediction was defined using an FDR < 0.05.

### scRNA-seq

Single-cell 3′ RNA (gene expression) libraries were generated according to the ‘Chromium Single Cell 3′ Reagents Kits User Guide (v3.1 Chemistry)’ (CG000204, 10x Genomics). For each sample, CD45^+^ cells from a complete liver were loaded into individual wells of the Chromium Chip G (Chromium Next GEM Chip G Single Kit, 1000127). GEM generation, reverse transcription, cDNA amplification and library preparation were performed using the Chromium Next GEM Single Cell 3′ GEM, Library and Gel Bead Kit v3.1 (1000121) according to the standard protocol. Size selection was performed using AMPure XP beads (A63881, Beckman Coulter). cDNA concentration was determined using the D5000 reagent kit (5067-5589, Agilent Technologies) and the library PCR cycle number was adjusted accordingly. The libraries were uniquely indexed using single-index primers from separate wells of the Single Index Kit T Set A plate (3000431). Quality control and molarity calculations of the final libraries were performed with the Qubit 3.0 Fluorometer (Q33216, Invitrogen) and the 4200 Tapestation system (Agilent Technologies). Libraries were pooled in a single 10 nM reaction for sequencing at the NGS Core Facility at the DKFZ according to Chromium Single Cell 3′ Reagents Kits User Guide v3.1 Chemistry (CG000204, 10x Genomics). Library pools were sequenced on two lanes of the NovaSeq 6000 system (v1.5 Reagent Kit). Sequencing was performed in a paired-end manner with a sequencing depth of at least 20,000 reads per cell and the read 1 sequenced for 28 cycles, i7 index for 8 cycles and read 2 for 91 cycles.

The 10x Genomics scRNA-seq data were processed using the qbic-pipelines/cellranger pipeline (v.1.01), which functions as a wrapper for the 10x Genomics cellranger-count pipeline (v.5.0.1) based on the nf-core framework^99^. The quality of the FastQ files was first assessed using FastQC (v.0.11.8) and aggregated for visualization using MultiQC^69^ (v.1.7; http://multiqc.info/). Raw reads were filtered and aligned to the reference mouse genome (UCSC mm10) to generate feature-barcode matrices for each sample. The UMI count matrix underwent preprocessing utilizing the Scanpy package (v.1.9.2) and the Seurat R package (v.2.4.3). After filtering out low-quality cells, defined as those expressing fewer than 200 genes or exhibiting a mitochondrial genome transcript ratio exceeding 0.2, a total of 19,690 cells was retained for subsequent analysis. Library size normalization was performed using Scanpy on the filtered matrix to obtain the normalized count. PCA was applied to the normalized expression matrix, focusing on highly variable genes. Cell clustering was performed using the graph-based Leiden clustering approach in Scanpy, and the results were visualized in two dimensions using UMAP. To interpret the developmental trajectory of CD8 T cells, we used the monocle package (v.2.24.0) using the DDRTree method. The GSVA package (v.1.48.3) was used to compute the pathway enrichment scores for individual cells. The gene sets used for this analysis included: HALLMARK-OXIDATIVE-PHOSPHORYLATION, WP-FATTY-ACID-BETAOXIDATION, WP-PURINE-METABOLISM, WP-AEROBIC-GLYCOLYSIS, KEGG-ARGININE-AND-PROLINE-METABOLISM, GOBP-ARGININE-CATABOLIC-PROCESS, KEGG-CITRATE-CYCLE-TCA-CYCLE, WP-FATTY-ACID-BIOSYNTHESIS, GOBP-FATTY-ACID-ELONGATION, WP-PENTOSE-PHOSPHATE-METABOLISM, KEGG-TRYPTOPHAN-METABOLISM, KEGG-PYRUVATE-METABOLISM, KEGG-N-GLYCAN-BIOSYNTHESIS, KEGG-GLYCOLYSIS-GLUCONEOGENESIS, GOBP-FATTY-ACID-BETA-OXIDATION, GOBP-FATTY-ACID-BIOSYNTHETIC-PROCESS, GOBP-ARGININE-METABOLIC_PROCESS.

### IMC analysis of human samples

The initial cohort comprised FFPE liver tissue of non-tumour (*n* = 4), tumour margin (*n* = 16) and tumour regions (*n* = 4) from patients with HCC (aetiology metabolic dysfunction- and alcohol-associated liver disease (MetALD), *n* = 2; MASH, *n* = 9; chronic hepatitis B virus infection (cHBV), *n* = 2; chronic hepatitis C virus infection (cHCV), *n* = 6; unknown, *n* = 3) and liver cirrhosis (*n* = 2) (prepared as a tissue microarray (TMA; diameter, 2 mm; thickness, 2 μm). The validation cohort consisted of FFPE slides obtained from non-tumour, margin and tumour regions from patients with HCC (non-tumour, *n* = 10; margin, *n* = 7; tumour, *n* = 10; aetiology: MetALD, *n* = 1; MASH, *n* = 1; cHBV, *n* = 1; cHCV, *n* = 4; unknown, *n* = 3; thickness, 2 μm). Written informed consent was obtained in all cases and the study was conducted according to the Declaration of Helsinki (1975), federal guidelines and local ethics committee regulations (Albert-Ludwigs-University, Freiburg, Germany, 20-1066).

Antibodies were labelled with the chosen metals according to the protocol of the Maxpar antibody labelling kit. Antibody staining was performed as described previously^100^. In brief, the slide was baked at 60 °C for 2 h and deparaffinization was performed in two ROTI Histol baths for 5 min followed by rehydration for 5 min each in a graded ethanol series (ethanol:deionized water 100:0, 100:0, 95:5, 80:20). The slide was washed in TBS pH 7.6. Heat-induced epitope retrieval was performed in a pressure cooker at 95 °C for 30 min in DACO EnVisionFlex target retrieval solution. After cooling the slide to room temperature in retrieval solution, it was washed in TBS for 10 min and blocked 45 min at room temperature with SuperBlock blocking buffer. Staining was performed with the antibody mix diluted in TBS containing 5% BSA and incubated overnight at 4 °C in a hydration chamber. The slide was next washed twice in TBS containing 0.2% Tween-20 for 5 min, Ir-intercalator for nuclear staining was added for 30 min at room temperature and the slide was washed three times with TBS, rinsed with ultrapure water, left to dry and was stored at room temperature until acquisition. For acquisition, a Helios time-of-flight mass cytometer (CyTOF) coupled to a Hyperion Imaging System (Fluidigm) was used. Tissue sections were laser-ablated spot-by-spot at 200 Hz for a total area of 1.5 μm × 0.75 μm. ROIs were determined using an ATF6α IHC and H&E staining of sequential tissue sections.

Correction for signal spillover of the metal isotopes was compensated using the Catalyst package in R; the script is available at GitHub (https://github.com/NiklasVesper/ImagingCytometryTools).

To analyse subcellular localization of antigens in the IMC data, a segmentation and analysis pipeline was developed. First, single-cell data for each individual cell was generated by image segmentation followed by detailed analysis of the segmented dataset. Cellular segmentation was performed with CellProfiler using the Cellpose 2.0 TissueNet segmentation model. The image overlay required for cellular segmentation was performed with the following proteins: CD4, CD8, CD15, CD20, CD68, SMA, E-cadherin and beta-catenin. For nuclear segmentation, Cellpose 2.0 CP segmentation model was used. Cytoplasmic segmentation was derived by subtracting the nuclear segmentation from the cellular (Supplementary Fig. 2a,b). The CellProfiler Cellpose 2.0 plugin was installed as described at GitHub (https://github.com/CellProfiler/CellProfiler-plugins). As a final step, all necessary data for further analysis from the segmented dataset was exported as a .csv file. The full CellProfiler pipeline for image segmentation with detailed settings and structure can be found on request at GitHub (https://github.com/NiklasVesper/ImagingCytometryTools).

For data analysis, a newly designed algorithm maps subcellular compartments (nuclei and cytoplasm) to corresponding cells by extracting the area of a cell and checking if there is a corresponding nucleus and cytoplasm within this area by matching the most central *xy*-coordinates of nuclei or cytoplasm, respectively (Supplementary Fig. 2c). Marker expression on the whole-cell or subcellular compartments was determined by gating on positive and negative cells/subcellular compartments in a histogram and image-based verification. Next, tumours and corresponding tissue were classified into ATF6α^hi^ and ATF6α^low^ tissue (Supplementary Fig. 2d,e). Further analysis was performed by OMIQ (https://www.omiq.ai/). Neighbourhood analysis was performed by setting an area of interest (radius = 1.5 × average cell diameter) around the cells and checking for the phenotype of the neighbouring cells. Next, the frequency of the lineage of the neighbouring cells were compared. All scripts for this analysis are available on request (https://github.com/NiklasVesper/ImagingCytometryTools).

### GeoMx DSP analysis of human samples

Manual slide preparation for GeoMx-NGS RNA was conducted according to the manufacturer’s guidelines (Bruker Spatial Biology). In brief, human HCC tissue FFPE microarrays (see the ‘Human samples’ section) were baked at 60 °C for 1 h before deparaffinization in CitriSolv and rehydration. The samples were incubated in target retrieval Tris EDTA solution at 99 °C for 15 min, RNA targets were exposed by permeabilization with proteinase K solution at 37 °C for 15 min and the samples were post-fixed in 10% formalin. In situ hybridization with RNA detection probes for the Cancer Transcriptome Atlas was done at 37 °C overnight before stringent washing, blocking and morphology marker staining for Syto13 (DNA dye), pan-CK and CD45 at room temperature for 1 h in a humidified chamber. The slides were loaded into the GeoMx Digital Spatial Profiler (DSP) to select ROIs for instrument cycling and ROI collection per well into a 96-well plate. After data collection, the samples were amplified by PCR, pooled and assessed for RNA quality control before sequencing on the NextSeq 500 (Illumina) system at the SBP Genomics core. FastQ files were transferred back to the GeoMx DSP for data analysis that included quality control, scaling and normalization, visualizations and statistical tests.

### CUT&RUN, ATAC–seq and data analysis

The CUT&RUN, ATAC–seq and related data analysis were done in collaboration with M.M. and B.G.R. as previously described^101^. In brief, livers from mice were collected and directly processed for nucleus isolation using liver swelling buffer (10 mM Tris pH 7.5, 2 mM MgCl_2_, 3 mM CaCl_2_) with a douncer. Liver homogenates were passed through a 70-µm strainer, centrifuged (400*g*, 5 min, 4 °C) and the pellets were resuspended in lysis buffer. The samples were centrifuged (400*g*, 5 min, 4 °C) and the pellets washed twice in PBS. Nucleus numbers were counted and ready for OMICS sample preparation.

For CUT&RUN, 500,000 nuclei from livers of *TG*^*Alb-cre+*^ mice were used following standard protocol^101^. Primary antibody (rabbit anti-ATF6α, SAB biotech 32008 or rabbit anti-HA, Abcam, ab9110) or control (mouse IgG) was used (5 μg for target antibody and 1 μg for IgG). Libraries were sequenced using NextSeq 2000 P3 Reagents (50 Cycles) v3 (Illumina, 20046810) on the NextSeq 2000 platform (Illumina). Data were analysed using the nf-core/cutandrun pipeline v.3.2.2 with Nextflow v.24.04.2, using the default parameters and following software dependencies: bedtools (v.2.30.0), bowtie (v.2.4.4), deeptools (v.3.5.1), fastqc (v.0.12.1), picard (v.3.1.0), Python (v.3.9.12), samtools (v.1.17), Genrich (v.0.6.1), TrimGalore (v.0.6.6), ucsc (v.377). CUT&RUN analysis identified direct target genes by using the HOMER’s annotatePeaks.pl tool on the final peak set and selecting genes with a peak located within ±1 kb of their transcription start site.

For ATAC–seq, 50,000 nuclei from livers of *TG*^*Alb-cre−*^ or *TG*^*Alb-cre+*^ mice were used. Libraries were sequenced on the NextSeq 2000 platform (Illumina). ATAC–seq data were analysed using the nf-core/atacseq pipeline v.2.1.2 with Nextflow v.24.0.2 using the default parameters^101^.

### MIBI analysis

Tissue sections (4 μm) were cut from mouse liver/tumour FFPE tissue blocks and subjected to MIBI. Staining, acquisition and data analysis were performed in collaboration with F.J.H. as previously described^43^.

Tissue slides were deparaffinized by incubation at 70 °C for 20 min, followed by three xylene washes. Rehydration was done by graded ethanol series (twice with 100% ethanol; twice with 95% ethanol; once with 80% ethanol; once with 70% ethanol) followed by a wash with distilled water. Antigen retrieval used epitope retrieval buffer (pH 9) with slides incubated at 97 °C for 40 min and cooled to 65 °C using the Lab Vision PT Module (Thermo Fisher Scientific). The slides were washed with MIBI wash buffer, composed of low-barium PBS-based IHC Tween buffer supplemented with 0.1% BSA. Tissues were blocked for 1 h with 1× TBS IHC wash buffer with Tween-20, 2% donkey serum, 0.1% cold fish skin gelatin, 0.1% Triton X-100 and 0.05% sodium azide. Primary antibodies were diluted in 3% donkey serum TBS IHC wash buffer and filtered through a 0.1 µm PVDF membrane before staining. The slides were incubated with primary antibodies overnight at 4 °C. The slides were washed twice with MIBI wash buffer and fixed for 5 min in 2% glutaraldehyde in low-barium PBS. Finally, the slides were dehydrated through three washes in Tris buffer (0.1 M, pH 8.5) and two washes in distilled water followed by a graded ethanol series (once with 70%; once with 80%; twice with 95%; and twice with 100%). The slides were stored in a vacuum chamber until imaging.

Images were acquired using the MIBI in coarse mode, capturing fields of view of 800 µm × 800 µm per sample. Regions enriched in CD8^+^ T cells were selected based on visual inspection of corresponding IHC images. After image acquisition, raw ion count data were processed into multiplexed images using the toffy package for noise filtering, intensity normalization and channel compensation.

Cell segmentation was performed using Cellpose 2.0 with the ‘TN2’ model Python package for pretrained neural network segmentation. Single-cell marker intensities were calculated by marker expression average across all pixels within each segmented cell mask. Cells beyond the acceptable range were excluded (<71 pixels (0.1 percentile) or >3,318 pixels (99.5 percentile), nuclear sum intensity below 9.21 a.u., or nuclear proportion outside the range of 0.3% to 99.8%). Marker expression values were normalized and capped at the 99.9 percentile across all retained cells, multiplied by a factor of 10 and arcsinh-transformed. CD8^+^ T cells were identified using FlowSOM, based on CD45, CD3 and CD8 expression. FlowSOM clustering was implemented using the Python version of the algorithm. LDH expression was visualized at the single-cell level using contour plots, with cells from treatment and control groups colour-coded in blue and red, respectively. Statistical significance was assessed using the Mann–Whitney *U*-tests. Moreover, the distribution of LDH expression was visualized using overlaid kernel density estimation plots, comparing four experimental conditions. Differences in cumulative distributions were evaluated using the Kolmogorov–Smirnov test. Low-level processing is available at GitHub (https://github.com/a-ngelolab/toffy) and the cell segmentation pipeline available at GitHub (https://github.com/mouseland/cellpose).

### Statistical analyses

Pilot experiments and previously published results were used to estimate the sample size, such that appropriate statistical tests could yield significant results. No further statistical methods were used to predetermine sample size. Measurements were taken from distinct biological samples, unless otherwise indicated. Mice were randomly allocated into different groups to make sure that the phenotype was homogeneous across groups, and were then fed with appropriate diet and/or administered their respective treatment regimens. Randomization of human patients was not applicable as no prospective trial/study was performed and human samples evaluated were obtained from pre-existing human patient cohorts/databases, adhering to ethical guidelines. Investigators were blinded to group allocation for all experiments in which blinding was technically feasible. Blinding was not possible for studies comparing preclinical liver cancer mouse models in which features were visually distinguishable (for example, normal chow pellets were brown, HFD pellets were blue; obese versus lean phenotypes; liver tumours inherently visible during dissection). Human patient data underwent pseudonymisation and were blinded to the analyser.

Data were collected in Microsoft Excel. Unless otherwise indicated, data are presented as mean ± s.e.m. (for example, scatter dot plot data). Violin plot data are presented showing all points with a dotted line at the median. Box and whisker plot data are presented with boxes spanning the 25th to 75th percentiles, with a line at the median, and whiskers from the minimum to maximum value. Statistical analysis was performed using GraphPad Prism software v.9.3.1 and v.10.0.3 (GraphPad Software). The normality assumption of the data distribution was verified using the Shapiro–Wilk test before performing the suitable statistical tests. For two-group comparisons, normally distributed data were analysed using two-tailed unpaired* t*-tests, and non-normal data were analysed using two-tailed unpaired Mann–Whitney *U*-tests. For comparisons of more than two groups, data were analysed using one-way or two-way ANOVA and corrected for multiple comparisons using statistical hypothesis testing (Tukey’s post hoc test), where applicable. Tumour incidence was analysed by *χ*^2^ test for contingency. Kaplan–Meier survival curves were analysed by a log-rank (Mantel–Cox) test. Where appropriate, false-discovery rate (FDR) corrections for multiple-hypothesis testing were performed. Exact *P* values between 0.0001 and 0.05 are reported. Sample sizes and statistical tests used are indicated in the legends or in the subsection below.

### Sample sizes, biological replicates and statistical tests

For Fig. 1b,c, ATF6α^hi^,* n* = 185 patients; ATF6α^low^, *n* = 185 patients; data were obtained from the TCGA-LIHC database. For Fig. 1d,e, *n* = 473 patients, with *n* = 120 (ATF6α^−^) and *n* = 353 (ATF6α^+^) patients; ATF6α^low^, *n* = 130, with *n* = 46 (G1) and *n* = 84 (G2) patients; ATF6α^hi^, *n* = 223 with *n* = 22 (G1), *n* = 139 (G2), *n* = 58 (G3) and *n* = 4 (undefined) patients. For Fig. 1g, *n* = 7 patients each with NT (non-tumour) or T (tumour) samples. For Fig. 1i, non-tumour (*n* = 32), tumour margin (*n* = 34), tumour (*n* = 32). For Fig. 1k, ATF6α^low^, *n* = 8 ROIs; ATF6α^hi^, *n* = 16 ROIs. For Fig. 1o–q, *n* = 10 patients. For Fig. 1r, *n* = 83 patients. Scatter dot plot data are presented as mean ± s.e.m. Violin plot data are presented showing all points with a dotted line at the median. Box and whisker plot data are presented with boxes spanning the 25th to 75th percentiles, with a line at the median, and whiskers from the minimum to maximum values. Data in Fig. 1a were analysed using Pearson correlation coefficient with Fisher’s exact test. Data in Fig. 1b,c were analysed using median split and log-rank test. Data in Fig. 1g were analysed using two-way ANOVA. Data in Fig. 1i were analysed using one-way ANOVA with Geisser–Greenhouse correction with Tukey’s post hoc test. D’Agostino–Pearson, Shapiro–Wilk and Kolmogorov–Smirnov tests were performed to test for normal distribution. Data in Fig. 1p were analysed using two-tailed Mann–Whitney *U*-tests. Data in Fig. 1q were analysed using Wilcoxon tests and Mann–Whitney *U*-tests. Data in Fig. 1r were analysed using Wilcoxon tests.

For Fig. 2b,c, 3-month-old *TG*^*Alb-cre−*^ mice: *n* = 8, 5 male and 3 female mice; 6-month-old *TG*^*Alb-cre−*^ mice: *n* = 14, 9 male and 5 female mice; 3-month-old *TG*^*Alb-cre+*^ mice: *n* = 10, 7 male and 3 female mice; 6-month-old *TG*^*Alb-cre+*^ mice: *n* = 18, 10 male and 8 female mice. For Fig. 2e,g,h, *TG*^*Alb-cre−*^ mice: *n* = 5; *TG*^*Alb-cre+*^ mice: *n* = 7. For Fig. 2i, *n* = 6 mice per group. For Fig. 2j, CUT&RUN: *TG*^*Alb-cre+*^ mice: *n* = 5; ATAC–seq: *TG*^*Alb-cre−*^ mice: *n* = 4; *TG*^*Alb-cre+*^ mice: *n* = 5. For Fig. 2l,m, *TG*^*AAV-gfp*^ mice: *n* = 12; *TG*^*AAV-cre*^ mice: *n* = 9; and *TG*^*AAV-cre/fbp1*^ mice: *n* = 8. For Fig. 2n, *n* = 3 mice per group. For Fig. 2p, *n* = 4 mice per group. Scatter dot plot data are presented as mean ± s.e.m. Data in Fig. 2b,c were analysed using two-tailed unpaired *t*-tests between age-matched *TG*^*Alb-cre−*^ and *TG*^*Alb-cre+*^ mice at the designated timepoint. Data in Fig. 2l,m were analysed using one-way ANOVA.

For Fig. 3a, *TG*^*Alb-cre−*^ mice: *n* = 17, 8 male and 9 female mice; *TG*^*Alb-cre+*^ mice:* n* = 43, 17 male and 26 female mice. For Fig. 3b, *TG*^*Alb-cre−*^ mice: *n* = 35, 20 male and 15 female mice; *TG*^*Alb-cre+*^ mice: *n* = 30, 11 male and 19 female mice. For Fig. 3d,e,h, 9-month-old *TG*^*Alb-cre−*^ mice: *n* = 20, 10 male and 10 female mice; 9-month-old *TG*^*Alb-cre+*^ mice: *n* = 33, 13 male and 20 female mice; 12-month-old *TG*^*Alb-cre−*^ mice: *n* = 29, 12 male and 17 female mice; 12-month-old *TG*^*Alb-cre+*^ mice: *n* = 49, 25 male and 24 female mice. For Fig. 3i, 12-month-old *TG*^*Alb-cre+*^ mice: *n* = 6; samples from patients with HCC, 151. For Fig. 3k–o, *TG*^*AAV-gfp*^ mice: *n* = 11, 6 male and 5 female mice; *TG*^*AAV-cre*^ mice: *n* = 12, 7 male and 5 female mice. For Fig. 3p, *TG*^*AAV-gfp*^ mice: *n* = 3; *TG*^*AAV-cre*^ mice: *n* = 5. For Fig. 3q, *n* = 371 patients, data were obtained from the TCGA-LIHC database. Scatter dot plot data and line graph data are presented as mean ± s.e.m. Data in Fig. 3a were analysed using the log-rank (Mantel–Cox) test. Data in Fig. 3b were calculated for the area under the curve and analysed using two-tailed Student’s* t*-tests. Data in Fig. 3d,e,l,m were analysed using Mann–Whitney *U*-tests. Data in Fig. 3k,n were analysed using two-tailed unpaired *t*-tests. Data in Fig. 3h,o were analysed using *χ*^2^ tests for contingency. Data in Fig. 3i syntenic mouse–human cancer genome copy-number alteration (CNA) concordance was calculated by mapping mouse CNA-positive regions to their human homologues, determining for each pair whether the same CNA type exceeded a 5% frequency threshold in human samples, and summarizing all regions of a given CNA type into a contingency table analysed using two-tailed Fisher’s exact test. Data in Fig. 3q were analysed using empirical permutation and correlation measured by IC as further described in Methods: Gene signatures and database analyses.

For Fig. 4b,c, *Atf6*^*+/+*^ mice: *n* = 9; *Atf6*^−/−^ mice: *n* = 14. For Fig. 4f, *n* = 3 mice per group. For Fig. 4h,i, *Atf6*^*fl/fl*^ mice: *n* = 11; *Atf6*^Δ^^*Hep*^ mice: *n* = 12. For Fig. 4k, *Atf6*^*fl/fl*^ mice: *n* = 6; *Atf6*^Δ^^*Hep*^ mice: *n* = 5. Fig. 4l: *n* = 9 mice per group. For Fig. 4n, *n* = 10 mice per group. For Fig. 4t: untreated, *n* = 9 mice; GalNac-ASO-scramble: *n* = 7 mice; GalNac-ASO-*Atf6*: *n* = 6 mice. For Fig. 4w, GalNac-ASO-scramble: *n* = 8 mice; GalNac-ASO-*Atf6*: *n* = 12 mice. Scatter dot plot data are presented as mean ± s.e.m. Data in Fig. 4c,i,l,n,w were analysed using two-tailed unpaired *t*-tests or Mann–Whitney *U*-tests based on data normality distribution. Data in Fig. 4b,h were analysed using *χ*^2^ tests for contingency. Data in Fig. 4t were analysed using one-way ANOVA.

For Fig. 5a, 3-month-old *TG*^*Alb-cre−*^ mice: *n* = 5; *TG*^*Alb-cre+*^ mice: *n* = 7; 3-month-old *TG*^*AAV-cre*^ and *TG*^*AAV-cre/fbp1*^ mice: *n* = 4 mice per group; 30-week-old DEN/HFD-treated *TG*^*AAV-gfp*^ mice: *n* = 3; and *TG*^*AAV-cre*^ mice: *n* = 5 with pair-matched non-tumour and tumour samples; 38-week-old DEN/HFD-treated *Atf6*^*+/+*^ and *Atf6*^−/−^ mice: *n* = 3 mice per group. For Fig. 5b,c, *TG*^*Alb-cre−*^ mice: *n* = 6; *TG*^*Alb-cre+*^ mice: *n* = 7. For Fig. 5e–h, *n* = 4 mice per group. For Fig. 5j, anti-IgG: *n* = 8 mice; anti-PD-1: *n *= 10 mice. For Fig. 5l, *n* = 3 mice per group. For Fig. 5n: *TG*^*Alb-cre+*^ mice: *n* = 19, 9 male and 10 female; *TG:Pdcd1*^−/−^ mice: *n* = 18, 8 male and 10 female. For Fig. 5p, *TG*^*Alb-cre+*^ mice: *n* = 43; *TG:Pdcd1*^−/−^ mice: *n* = 53. Scatter dot plot data are presented as mean ± s.e.m. Data in Fig. 5a were analysed using nonparametric Wilcoxon and Kruskal–Wallis tests. Data in Fig. 5b,c,j were analysed using two-tailed unpaired *t*-tests. The distribution of LDH expression in Fig. 5l was visualized using overlaid KDE plots. Differences in cumulative distributions were evaluated using the Kolmogorov–Smirnov tests. Data in Fig. 5n were analysed using two-tailed unpaired Mann–Whitney *U*-tests. Data in Fig. 5p were analysed using the log-rank (Mantel–Cox) test.

Mouse icons used in Figs. 2–5 were created using BioRender (Heikenwälder, M. (2026); https://BioRender.com/lgjnsy9).

### Reporting summary

Further information on research design is available in the Nature Portfolio Reporting Summary linked to this article.

## Online content

Any methods, additional references, Nature Portfolio reporting summaries, source data, extended data, supplementary information, acknowledgements, peer review information; details of author contributions and competing interests; and statements of data and code availability are available at 10.1038/s41586-025-10036-8.

## Supplementary information

Supplementary Information Supplementary Figs. 1–11 and Supplementary Tables 1–3.

Reporting Summary

Supplementary Data Source data for Supplementary Figs. 1–7 and 9.

## Source data

Source Data Figs. 1–5.

Source Data Extended Data Figs. 1–12.

## Data Availability

The proteomics data are available at ProteomeXchange Consortium through the PRIDE database under project accession PXD045903. The bulk RNA-seq data are available under GEO SuperSeries GSE244344 (GSE244341: *TG*^*AAV-gfp*^, *TG*^*AAV-cre*^ and *TG*^*AAV-cre/fbp1*^; GSE244342: *TG*^*AAV-gfp*^ and *TG*^*AAV-cre*^ + DEN/HFD; GSE244343: *Atf6*^*+/+*^ and *Atf6*^−/−^ DEN/HFD), and GSE244212 (3M *TG*^*Alb-cre−*^ and *TG*^*Alb-cre+*^), GSE244213 (6M *TG*^*Alb-cre−*^ and *TG*^*Alb-cre+*^) and GSE285265 (*Atf6*^*fl/fl*^ and *Atf6*^*ΔHep*^ mice + CD-HFD). The scRNA-seq data are available under GEO GSE243826 and GSE285366. The CUT&RUN data are available under GEO GSE285262. The ATAC–seq data are available under GEO GSE285261. The array of CGH data is available under GEO GSE242831. The NMR-based metabolomics data and LC–MS/MS metabolic analysis are available at the Metabolights database through the study MTBLS13241. The results here are in part based on data generated by the TCGA Research Network (https://www.cancer.gov/tcga). Databases used in this Article included MSigDB (www.broadinstitute.org/msigdb). Source data are provided with this paper.
