# Supplementary material for: Activated ATF6α is a hepatic tumour driver restricting immunosurveillance
Source: Nature. 2026 Feb 4;651(8106):796–807. doi: 10.1038/s41586-025-10036-8 (PMC12999494; doi:10.1038/s41586-025-10036-8)
Supplement: Supplementary file 1 — Supplementary Figs. 1–11 and Supplementary Tables 1–3. [file 41586_2025_10036_MOESM1_ESM.pdf]

nature portfolio

https://doi.org/10.1038/s41586-025-10036-8

Supplementary information

Activated ATF6α is a hepatic tumour driver restricting immunosurveillance

In the format provided by the authors and unedited

Supplementary Fig. 1

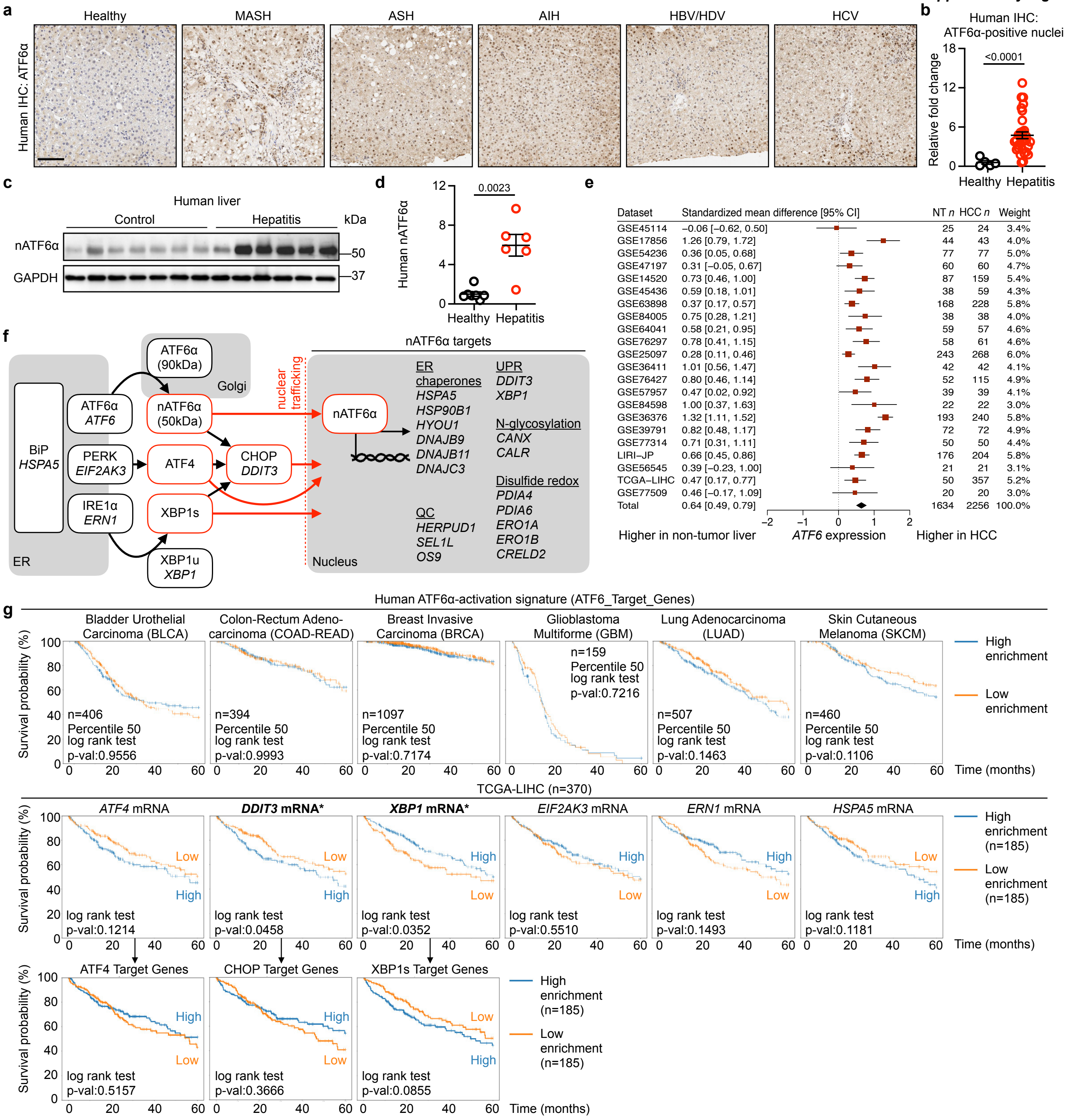

Supplementary Fig. 1: High levels of ATF6α-activation are found in human patients with chronic liver disease *en route* to hepatocellular carcinoma (HCC), Related to Figure 1.

(a) Representative IHC of ATF6α in healthy human liver (donors for liver transplantation, n=5) and diseased human liver tissue (scale bar = 200μm; MASH = Metabolic dysfunction-Associated Steatohepatitis, n=15; ASH = Alcoholic Steatohepatitis, n=5; AIH = Autoimmune hepatitis, n=6; HBV/HDV = Hepatitis B/D Virus, n=3; HCV = Hepatitis C Virus, n=4). **(b)** Quantification of ATF6α-positive nuclei by IHC in control (n=5) and hepatitis (n=33) human liver tissue. **(c-d)** Immunoblot analysis (**c**) and quantification (**d**) of nATF6α in control (n=7) and hepatitis (n=6) human liver tissue. Control: liver biopsy with NAS score≤3, no inflammation/steatohepatitis. Hepatitis: Liver biopsy from inflamed non-tumor tissue. GAPDH as loading control, ran on nATF6α blot. kDa: kilodalton. **(e)** Forest plot of *ATF6* mRNA expression in HCC versus non-tumor liver tissue in 22 datasets. **(f)** Schematic of activated unfolded protein response (UPR) signaling and the induction of nATF6α target genes (gene names italicized). **(g)** Kaplan-Meier survival curves for The Cancer Genome Atlas (TCGA) patients divided into two groups by median split based on high or low enrichment of the human ATF6α-activation signature (MSigDB Human Gene Set: ATF6_Target_Genes; top), UPR-related gene mRNA expression (middle) or enrichment of transcription factor target genes (MSigDB Human Gene Sets: ATF4_Q2, CHOP_01, XBP1_01; bottom). Cohort patient numbers (n) vary by dataset, as indicated within each plot. Bold labeling depicts significant genes. Scatter dot plot data are presented as mean values ± SEM. Data in 1b,d were analyzed by two-tailed Mann-Whitney test based on data normality distribution. Data in 1g were analyzed by median split and log-rank test.

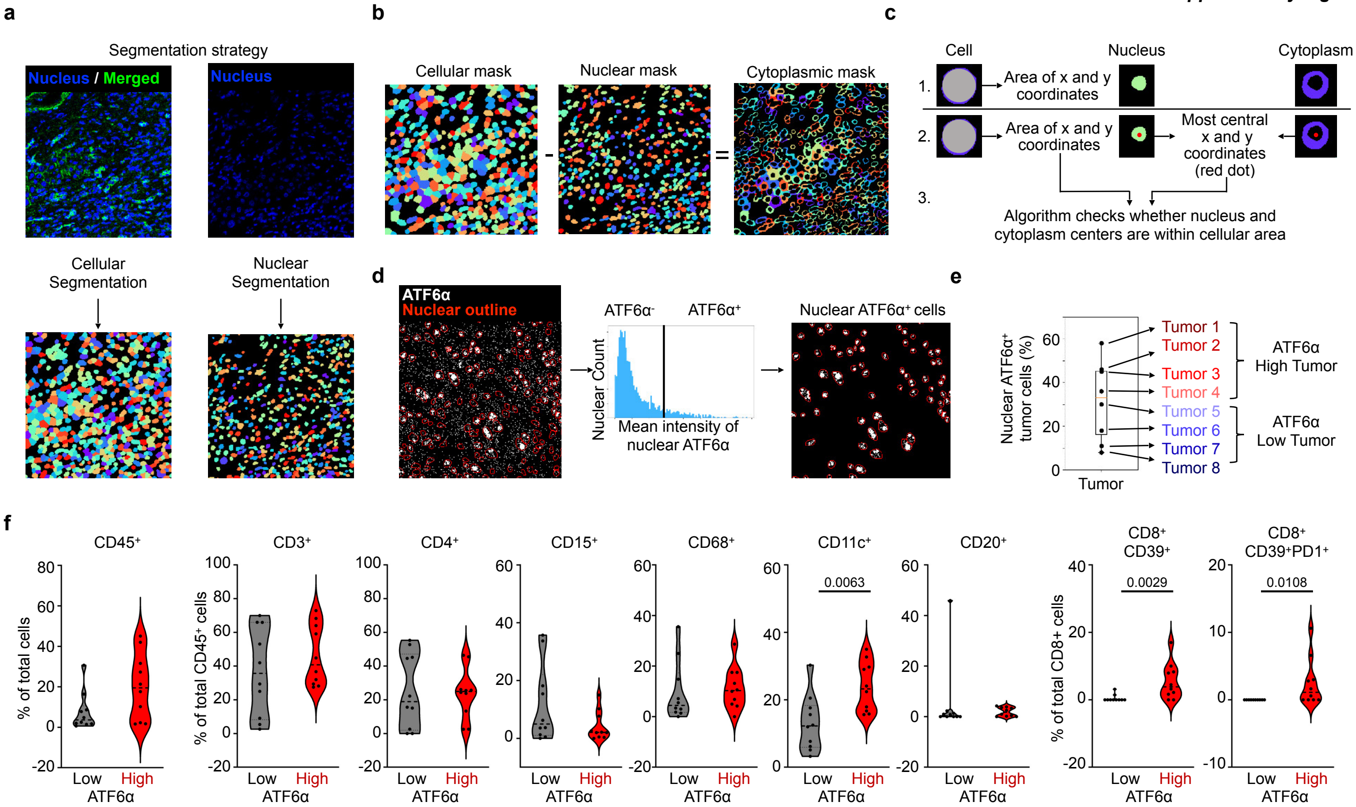

a
Segmentation strategy
Nucleus / Merged
Nucleus
Cellular Segmentation
Nuclear Segmentation
b
Cellular mask
Nuclear mask
Cytoplasmic mask
c
Cell
Nucleus
Cytoplasm
1.
Area of x and y coordinates
2.
Area of x and y coordinates
Most central x and y coordinates (red dot)
3.
Algorithm checks whether nucleus and cytoplasm centers are within cellular area
d
ATF6α
Nuclear outline
ATF6α-
ATF6α+
Nuclear Count
Mean intensity of nuclear ATF6α
Nuclear ATF6α+ cells
e
Nuclear ATF6α+ tumor cells (%)
Tumor
Tumor 1
Tumor 2
Tumor 3
Tumor 4
Tumor 5
Tumor 6
Tumor 7
Tumor 8
ATF6α High Tumor
ATF6α Low Tumor
f
CD45+
CD3+
CD4+
CD15+
CD68+
CD11c+
CD20+
CD8+ CD39+
CD8+ CD39+PD1+
% of total cells
% of total CD45+ cells
% of total CD8+ cells
0.0063
0.0029
0.0108
Low
High
ATF6α

Supplementary Fig. 2: Imaging mass cytometry (IMC) analysis of human ATF6α-high versus ATF6α-low HCC samples, Related to Figure 1.

(a) Initial segmentation masks consisting of cellular and nuclear segmentation masks. For cellular segmentation, both the nuclear channel (blue) and a merged channel of multiple other channels (green) for CD4, CD8, CD15, CD20, CD68, αSMA, e-cadherin and β-catenin were used. **(b)** The cytoplasmatic masks were derived from subtracting nuclear from cellular masks. **(c)** The area of a single cell was extracted from the mask in step 1. The most central x and y coordinates (red dot) of a nuclear and cytoplasm mask were extracted in step 2. The algorithm checks whether nucleus and cytoplasm centers are within the cellular area in step 3. **(d)** Visual gating strategy used to identify ATF6α (white) positive nuclei within (red) nuclear outlines. **(e)** Schematic representation of ATF6α-based classification of tumors by the percent of ATF6α-positive nuclei. The median was used to categorize ATF6α-high and ATF6α-low groups. **(f)** Quantification of indicated cell subset composition in the tumor tissue of ATF6α-low (n=10) and ATF6α-high (n=10) HCC patients. Data in 2f are presented as violin plots. Violin plot data are presented showing all points with a dotted line at the median. Data in 2f were analyzed by two-tailed unpaired t-test or Mann-Whitney test based on data normality distribution.

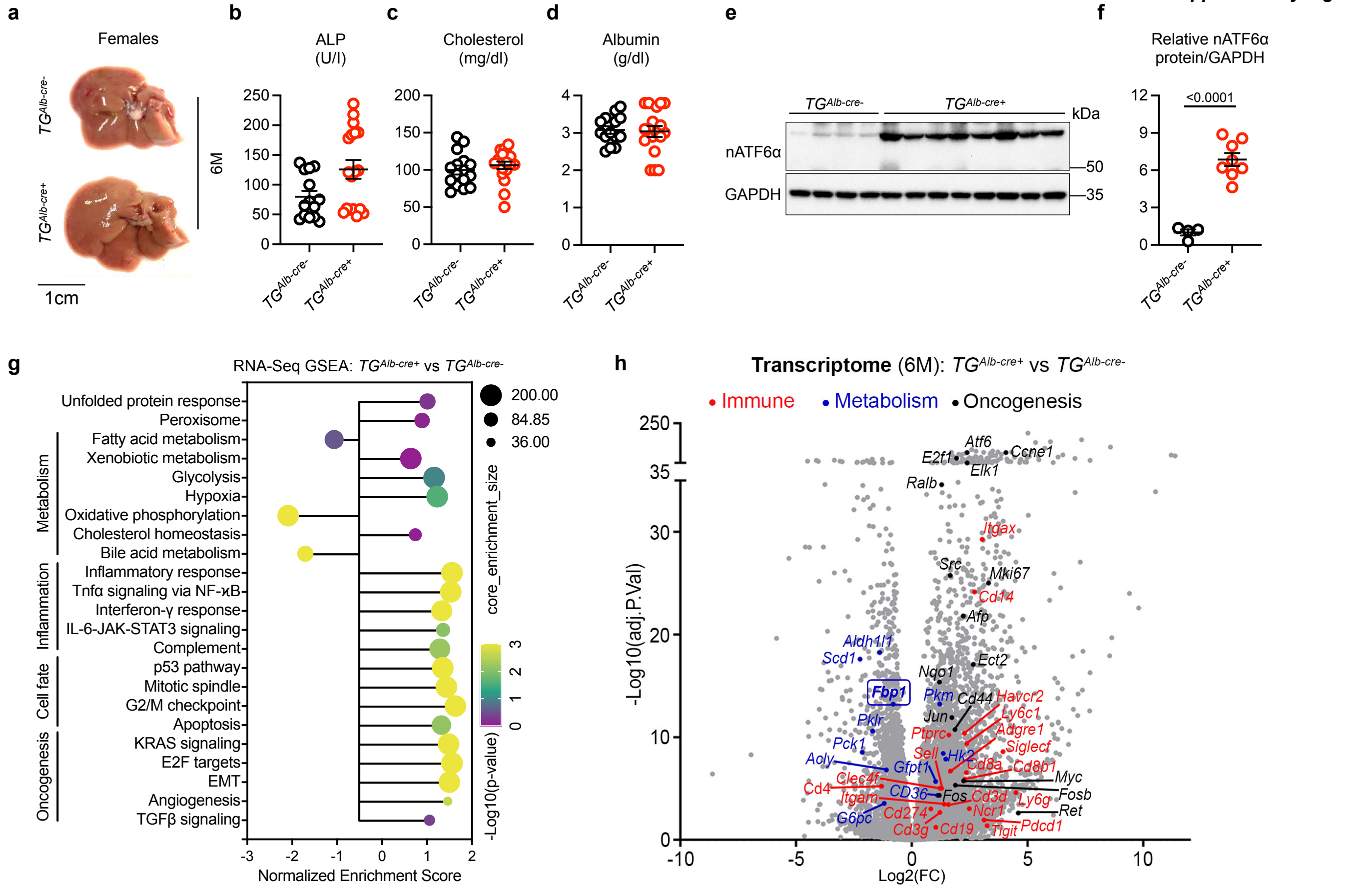
a
Females
TGAlb-cre-
TGAlb-cre+
6M
1cm
b
ALP
(U/l)
c
Cholesterol
(mg/dl)
d
Albumin
(g/dl)
e
TGAlb-cre-
TGAlb-cre+
kDa
nATF6α
GAPDH
50
35
f
Relative nATF6α
protein/GAPDH
<0.0001
g
RNA-Seq GSEA: TGAlb-cre+ vs TGAlb-cre-
Unfolded protein response
Peroxisome
Fatty acid metabolism
Xenobiotic metabolism
Glycolysis
Hypoxia
Oxidative phosphorylation
Cholesterol homeostasis
Bile acid metabolism
Inflammatory response
Tnfα signaling via NF-κB
Interferon-γ response
IL-6-JAK-STAT3 signaling
Complement
p53 pathway
Mitotic spindle
G2/M checkpoint
Apoptosis
KRAS signaling
E2F targets
EMT
Angiogenesis
TGFβ signaling
Metabolism
Inflammation
Cell fate
Oncogenesis
200.00
84.85
36.00
core_enrichment_size
-Log10(p-value)
Normalized Enrichment Score
h
Transcriptome (6M): TGAlb-cre+ vs TGAlb-cre-
Immune
Metabolism
Oncogenesis
-Log10(adj.P.Val)
Log2(FC)
Atf6
Ccne1
E2f1
Elk1
Ralb
Itgax
Src
Mki67
Cd14
Afp
Aldh1l1
Scd1
Ect2
Nqo1
Fbp1
Pkm
Cd44
Havcr2
Ly6c1
Jun
Pklr
Ptprc
Adgre1
Pck1
Siglecf
Sell
Hk2
Aoly
Gfpt1
Cd8a
Cd8b1
Myc
Clec4f
Cd4
CD36
Fos
Cd3d
Fosb
Itgam
Ly6g
G6pc
Cd274
Ncr1
Ret
Cd3g
Cd19
Tigit
Pdcd1

Supplementary Fig. 3: Persistent ATF6α-activation in hepatocytes of 6-month-old mice, Related to Figure 2.

(a) Representative liver macroscopic images of female $TG^{Alb\text{-}cre-}$ and $TG^{Alb\text{-}cre+}$ mice sacrificed at 6 months of age. **(b-d)** Serum ALP (**b**), cholesterol (**c**), and albumin (**d**) levels of 6-month-old $TG^{Alb\text{-}cre-}$ (n=14, 9 male and 5 female) and $TG^{Alb\text{-}cre+}$ (n=18, 10 male and 8 female) mice. **(e-f)** Representative immunoblot analysis (**e**) and quantification (**f**) of n-ATF6α in livers of 6-month-old $TG^{Alb\text{-}cre-}$ (n=4) and $TG^{Alb\text{-}cre+}$ (n=8) mice. GAPDH as loading control, ran on a separate blot, processed in parallel. kDa: kilodalton. **(g)** GSEA of RNA-seq data shows negatively and positively enriched pathways in livers of 6-month-old $TG^{Alb\text{-}cre+}$ (n=7) versus $TG^{Alb\text{-}cre-}$ (n=6) mice. **(h)** Volcano plot of RNA-seq data from livers of 6-month-old $TG^{Alb\text{-}cre+}$ (n=7) versus $TG^{Alb\text{-}cre-}$ (n=6) mice. Red dots indicate immune-related genes, blue dots indicate metabolic genes, and black dots indicate oncogenic genes. Scatter dot plot data are presented as mean values ± SEM. Data in 3b-d,f were analyzed by two-tailed unpaired t-test or Mann-Whitney test based on data normality distribution. Data in 3g were analyzed by GSEA based on the Kolmogorov-Smirnov test.

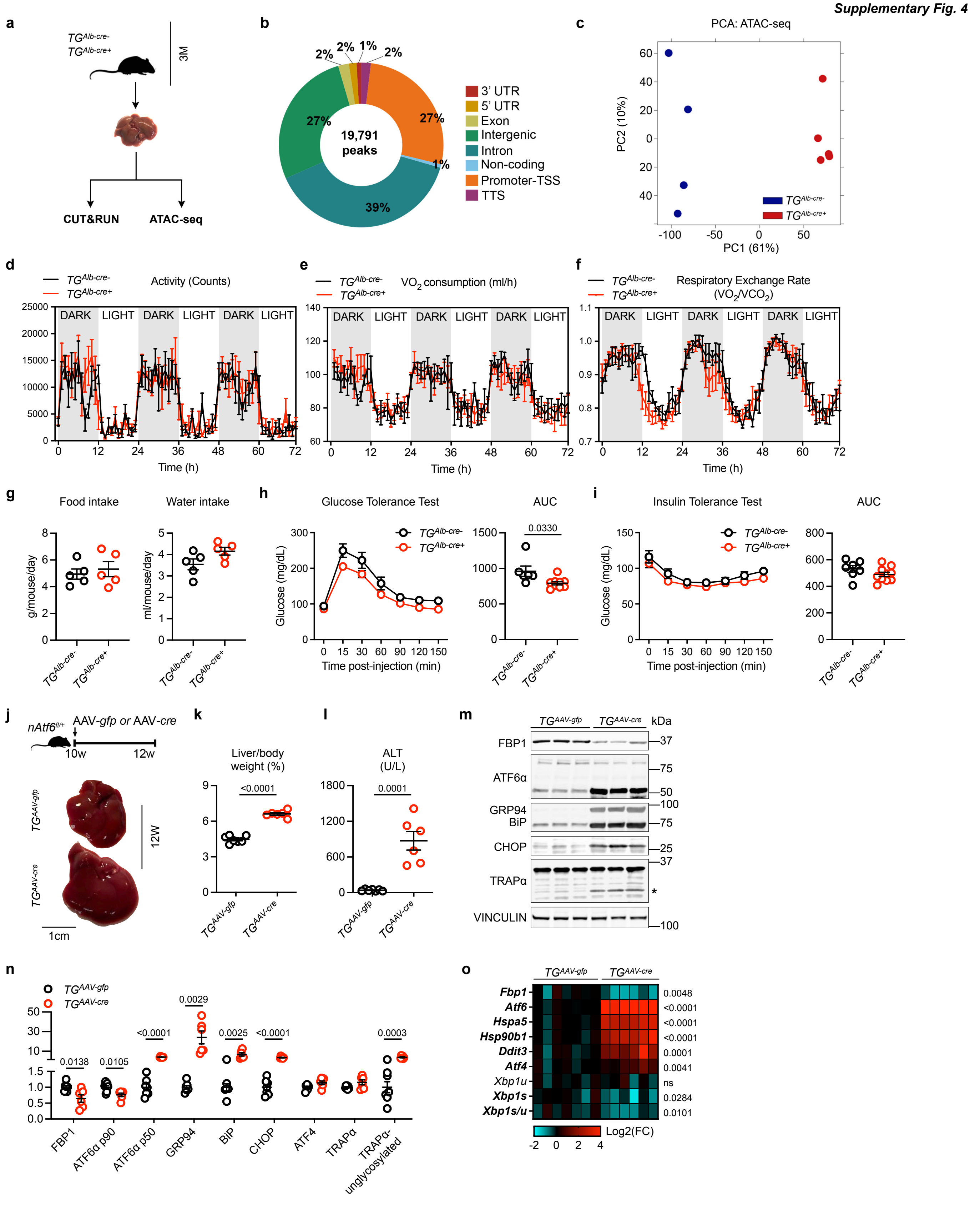
a
TGAlb-cre-
TGAlb-cre+
3M
CUT&RUN
ATAC-seq
b
19,791 peaks
3' UTR
5' UTR
Exon
Intergenic
Intron
Non-coding
Promoter-TSS
TTS
c
PCA: ATAC-seq
PC1 (61%)
PC2 (10%)
d
Activity (Counts)
DARK LIGHT DARK LIGHT DARK LIGHT
Time (h)
e
VO2 consumption (ml/h)
f
Respiratory Exchange Rate (VO2/VCO2)
g
Food intake
Water intake
g/mouse/day
ml/mouse/day
h
Glucose Tolerance Test
AUC
0.0330
Glucose (mg/dL)
Time post-injection (min)
i
Insulin Tolerance Test
AUC
j
nAtf6fl/+
AAV-gfp or AAV-cre
10w
12w
TGAAV-gfp
TGAAV-cre
12W
1cm
k
Liver/body weight (%)
<0.0001
l
ALT (U/L)
0.0001
m
TGAAV-gfp
TGAAV-cre
kDa
FBP1
ATF6α
GRP94
BiP
CHOP
TRAPα
VINCULIN
n
FBP1
ATF6α p90
ATF6α p50
GRP94
BiP
CHOP
ATF4
TRAPα
TRAPα-unglycosylated
0.0138
0.0105
<0.0001
0.0029
0.0025
<0.0001
0.0003
o
Fbp1 0.0048
Atf6 <0.0001
Hspa5 <0.0001
Hsp90b1 <0.0001
Ddit3 0.0001
Atf4 0.0041
Xbp1u ns
Xbp1s 0.0284
Xbp1s/u 0.0101
Log2(FC)

Supplementary Fig. 4. Hepatocyte ATF6α-activation reduces glucose tolerance and FBP1 expression while sustaining ATF6α-driven ER stress, Related to Figure 2.

(a) Scheme of livers harvested from 3-month-old *TG*$^{Alb\text{-}cre-}$ and *TG*$^{Alb\text{-}cre+}$ mice for CUT&RUN and ATAC-seq analyses. **(b)** Pie chart distribution of n-ATF6α chromatin binding regions in the genome by CUT&RUN (n=5, *TG*$^{Alb\text{-}cre+}$ mice). **(c)** Principal component analysis (PCA) of ATAC-seq showing unsupervised clustering of genomic profiles from livers of 3-month-old *TG*$^{Alb\text{-}cre-}$ (n=4) and *TG*$^{Alb\text{-}cre+}$ (n=5) mice. **(d-g)** Metabolic activity (**d**), oxygen consumption rate (**e**), respiratory exchange rate (**f**), food and water consumption (**g**) in 3-month-old *TG*$^{Alb\text{-}cre-}$ (n=5) and *TG*$^{Alb\text{-}cre+}$ (n=5) mice placed 72h in metabolic cages. **(h)** Glucose tolerance tests of 13h-fasted *TG*$^{Alb\text{-}cre-}$ (n=6) and *TG*$^{Alb\text{-}cre+}$ (n=9) mice at 3 months of age. **(i)** Insulin tolerance tests of 6h-fasted *TG*$^{Alb\text{-}cre-}$ (n=7) and *TG*$^{Alb\text{-}cre+}$ (n=10) mice at 3 months of age. **(j)** Representative liver macroscopic images of *nAtf6*$^{fl/+}$ mice sacrificed two weeks after AAV8-*gfp (TG*$^{AAV\text{-}gfp}$; n=7) or AAV8-*cre* (*TG*$^{AAV\text{-}cre}$; n=6) intravenous (i.v.) injection. **(k-l)** Liver-to-body weight (**k**) and serum ALT levels (**l**) of *TG*$^{AAV\text{-}gfp}$ (n=7) and *TG*$^{AAV\text{-}cre}$ (n=6) mice. **(m-n)** Representative immunoblot analysis (**m**) and quantification (**n**) of indicated proteins from livers of *TG*$^{AAV\text{-}gfp}$ (n=7) and *TG*$^{AAV\text{-}cre}$ (n=6) mice. VINCULIN as loading control, ran on a separate blot, processed in parallel. kDa: kilodalton. **(o)** qRT-PCR analysis of indicated mRNAs from livers of *TG*$^{AAV\text{-}gfp}$ (n=7) and *TG*$^{AAV\text{-}cre}$ (n=6) mice. Bold labeling depicts significant genes. Scatter dot plot and line graph data are presented as mean values ± SEM. Data in 4g-i,k,l,n,o were analyzed by two-tailed unpaired t-test or Mann-Whitney test based on data normality distribution. Mouse icons were created in BioRender. Heikenwälder, M. (2026) https://BioRender.com/lgjnsy9.

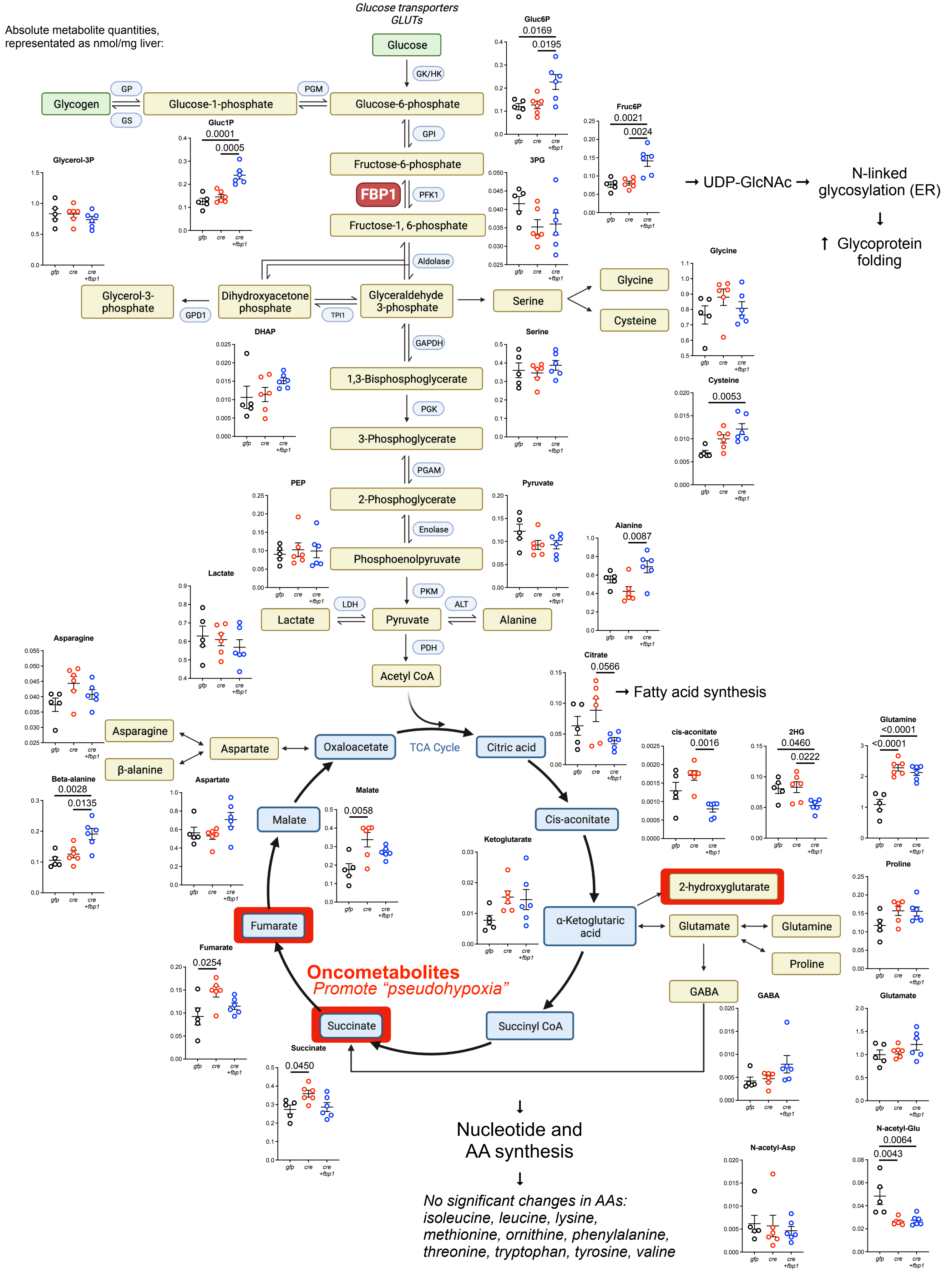

Absolute metabolite quantities, representated as nmol/mg liver:
Glucose transporters GLUTs
Glucose
GK/HK
Glycogen
GP
GS
Glucose-1-phosphate
PGM
Glucose-6-phosphate
GPI
Fructose-6-phosphate
FBP1
PFK1
Fructose-1, 6-phosphate
Aldolase
Glycerol-3-phosphate
GPD1
Dihydroxyacetone phosphate
TPI1
Glyceraldehyde 3-phosphate
Serine
Glycine
Cysteine
GAPDH
1,3-Bisphosphoglycerate
PGK
3-Phosphoglycerate
PGAM
2-Phosphoglycerate
Enolase
Phosphoenolpyruvate
PKM
LDH
ALT
Lactate
Pyruvate
Alanine
PDH
Acetyl CoA
→ UDP-GlcNAc →
N-linked glycosylation (ER)
↑ Glycoprotein folding
→ Fatty acid synthesis
Asparagine
β-alanine
Aspartate
Oxaloacetate
TCA Cycle
Citric acid
Cis-aconitate
Malate
α-Ketoglutaric acid
2-hydroxyglutarate
Glutamate
Glutamine
Proline
GABA
Fumarate
Succinate
Succinyl CoA
Oncometabolites
Promote “pseudohypoxia”
Nucleotide and AA synthesis
No significant changes in AAs: isoleucine, leucine, lysine, methionine, ornithine, phenylalanine, threonine, tryptophan, tyrosine, valine
Gluc6P 0.0169 0.0195
Fruc6P 0.0021 0.0024
Gluc1P 0.0001 0.0005
Glycerol-3P
3PG
Glycine
DHAP
Serine
Cysteine 0.0053
PEP
Pyruvate
Alanine 0.0087
Lactate
Asparagine
Citrate 0.0566
cis-aconitate 0.0016
2HG 0.0460 0.0222
Glutamine <0.0001 <0.0001
Beta-alanine 0.0028 0.0135
Aspartate
Malate 0.0058
Ketoglutarate
Proline
Fumarate 0.0254
GABA
Glutamate
Succinate 0.0450
N-acetyl-Asp
N-acetyl-Glu 0.0064 0.0043
gfp
cre
cre +fbp1

Supplementary Fig. 5. Catalytic activity of hepatocyte FBP1 promotes gluconeogenesis and increases hexose phosphates while preventing the ATF6α-activation-driven increase in TCA cycle-related oncometabolites, Related to Figure 2.

GC-MS analysis for metabolite quantities from livers of $TG^{AAV\text{-}gfp}$ (n=5), $TG^{AAV\text{-}cre}$ (n=6), and $TG^{AAV\text{-}cre/fbp1}$ (n=6) mice. Scatter dot plot data are presented as mean values ± SEM. Data were analyzed by one-way ANOVA and Tukey's post hoc test. Metabolite schematic was created in BioRender. Heikenwälder, M. (2026) https://BioRender.com/lgjnsy9.

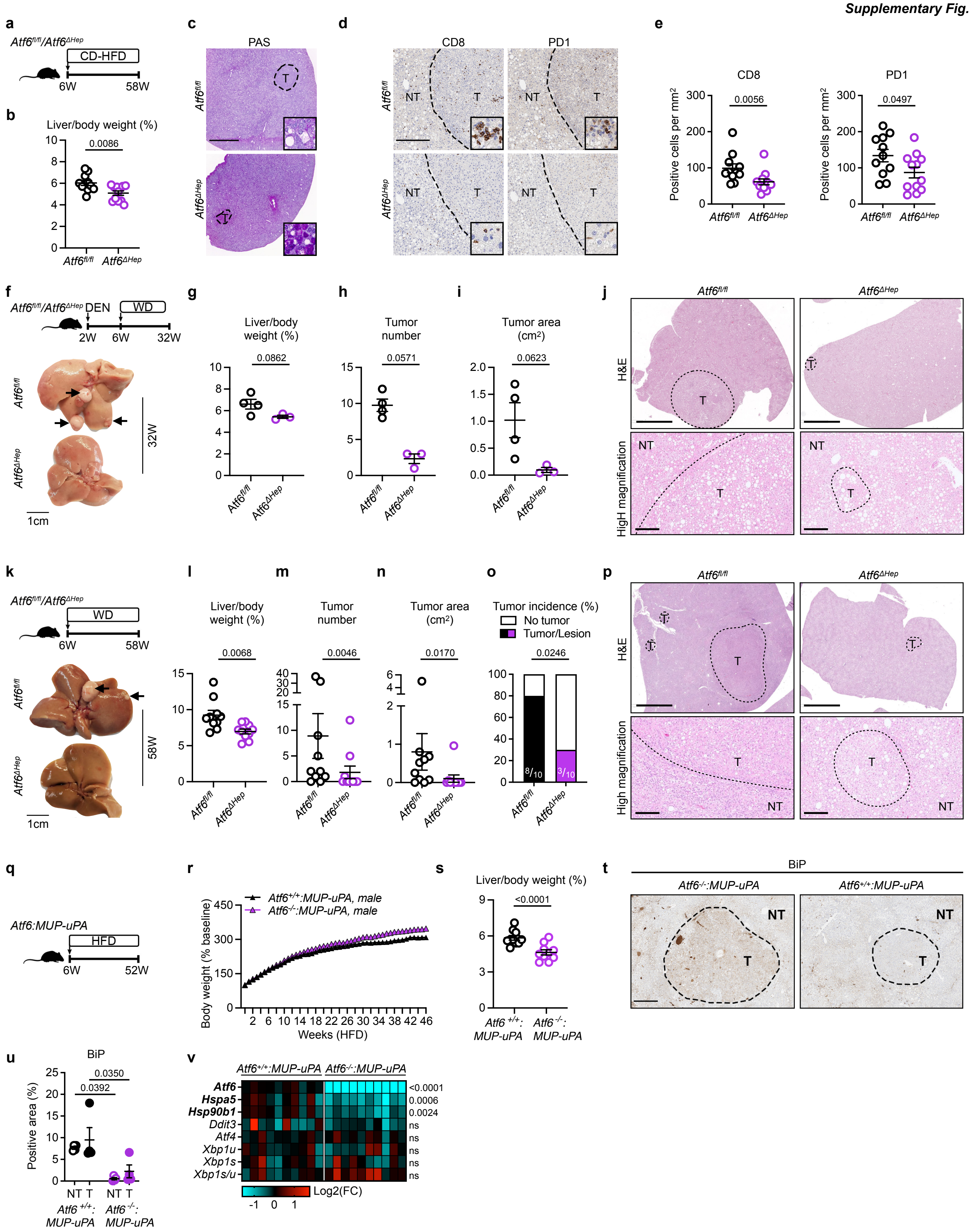
a
Atf6fl/fl/Atf6ΔHep
CD-HFD
6W
58W
b
Liver/body weight (%)
0.0086
c
PAS
d
CD8
PD1
NT
T
e
CD8
PD1
Positive cells per mm2
0.0056
0.0497
f
DEN
WD
2W 6W 32W
1cm
g
Liver/body weight (%)
0.0862
h
Tumor number
0.0571
i
Tumor area (cm2)
0.0623
j
H&E
HigH magnification
k
l
Liver/body weight (%)
0.0068
m
Tumor number
0.0046
n
Tumor area (cm2)
0.0170
o
Tumor incidence (%)
No tumor
Tumor/Lesion
0.0246
8/10
3/10
p
High magnification
q
Atf6:MUP-uPA
HFD
52W
r
Atf6+/+:MUP-uPA, male
Atf6-/-:MUP-uPA, male
Body weight (% baseline)
Weeks (HFD)
s
Liver/body weight (%)
<0.0001
t
BiP
Atf6-/-:MUP-uPA
Atf6+/+:MUP-uPA
u
BiP
Positive area (%)
0.0350
0.0392
v
Atf6
Hspa5
Hsp90b1
Ddit3
Atf4
Xbp1u
Xbp1s
Xbp1s/u
<0.0001
0.0006
0.0024
ns
Log2(FC)

Supplementary Fig. 6: Hepatocyte-specific *Atf6* deletion reduces tumor burden in obesogenic preclinical HCC models, Related to Figure 4.

(a) Scheme of *Atf6*$^{fl/fl}$ or *Atf6*$^{\Delta Hep}$ mice fed a choline-deficient high fat diet (CD-HFD) and sacrificed at 58 weeks of age. **(b)** Liver-to-body weight of CD-HFD-fed *Atf6*$^{fl/fl}$ (n=11) and *Atf6*$^{\Delta Hep}$ (n=12) mice. Scale bar = 3mm. **(c)** Representative liver PAS staining of CD-HFD-fed *Atf6*$^{fl/fl}$ and *Atf6*$^{\Delta Hep}$ mice. **(d-e)** Representative liver IHC of CD8 and PD-1 (**d**) with quantification (**e**) from CD-HFD-fed *Atf6*$^{fl/fl}$ (n=11) or *Atf6*$^{\Delta Hep}$ (n=12) mice. Scale bar = 300µm. **(f)** Scheme of *Atf6*$^{fl/fl}$ or *Atf6*$^{\Delta Hep}$ mice intraperitoneally injected with diethyl-nitrosamine (DEN; 25mg/kg) at 2 weeks of age prior to Western diet (WD)-feeding and sacrificed at 32 weeks of age (top), with representative macroscopic liver images (bottom); arrows indicate tumor nodules. **(g-i)** Liver-to-body weight (**g**), tumor number (**h**) and area (**i**, cm^2) per liver in DEN/WD-treated *Atf6*$^{fl/fl}$ (n=4) or *Atf6*$^{\Delta Hep}$ (n=3) mice. **(j)** Representative liver HE staining (scale bar = 3mm top, 200µm bottom) of DEN/WD-treated *Atf6*$^{fl/fl}$ or *Atf6*$^{\Delta Hep}$ mice. **(k)** Scheme of *Atf6*$^{fl/fl}$ or *Atf6*$^{\Delta Hep}$ mice fed a WD and sacrificed at 58 weeks of age (top), with representative macroscopic liver images (bottom); arrows indicate tumor nodules. **(l-o)** Liver-to-body weight (**l**), tumor number (**m**) and area (**n**, cm^2) per liver, or tumor incidence (**o**) in WD-fed *Atf6*$^{fl/fl}$ (n=10) and *Atf6*$^{\Delta Hep}$ (n=10) mice. **(p)** Representative liver HE staining (scale bar = 3mm top, 200µm bottom) of WD-fed *Atf6*$^{fl/fl}$ or *Atf6*$^{\Delta Hep}$ mice. **(q)** Scheme of *Atf6*$^{+/+}$*:MUP-uPA* and *Atf6*$^{-/-}$*:MUP-uPA* mice fed a HFD and sacrificed at 52 weeks of age. **(r)** Body weight gain during HFD-feeding of *Atf6*$^{+/+}$*:MUP-uPA* (n=10) and *Atf6*$^{-/-}$*:MUP-uPA* mice (n=10). **(s)** Liver-to-body weight of HFD-fed *Atf6*$^{+/+}$*:MUP-uPA* (n=10) and *Atf6*$^{-/-}$*:MUP-uPA* mice (n=10). **(t-u)** Representative BiP IHC (**t**) and quantification (**u**) from livers of HFD-fed *Atf6*$^{+/+}$*:MUP-uPA* (n=4) and *Atf6*$^{-/-}$*:MUP-uPA* mice (n=4). **(v)** qRT-PCR analysis of indicated mRNAs from livers of HFD-fed *Atf6*$^{+/+}$*:MUP-uPA* (n=10) and *Atf6*$^{-/-}$*:MUP-uPA* mice (n=10). Bold labeling depicts significant genes. T = tumor, NT = non-tumor. Scatter dot plot and line graph data are presented as mean values ± SEM. Data in 6b,e,g-i,l-n,s were analyzed by two-tailed unpaired t-test or Mann-Whitney test based on data normality distribution. Data in 6o were analyzed by Chi-square test for contingency. Data in 6u were analyzed by two-way ANOVA and Tukey's post hoc test. NT: non-tumor, T: tumor (within dotted lines). Mouse icons were created in BioRender. Heikenwälder, M. (2026) https://BioRender.com/lgjnsy9.

Supplementary Fig. 7

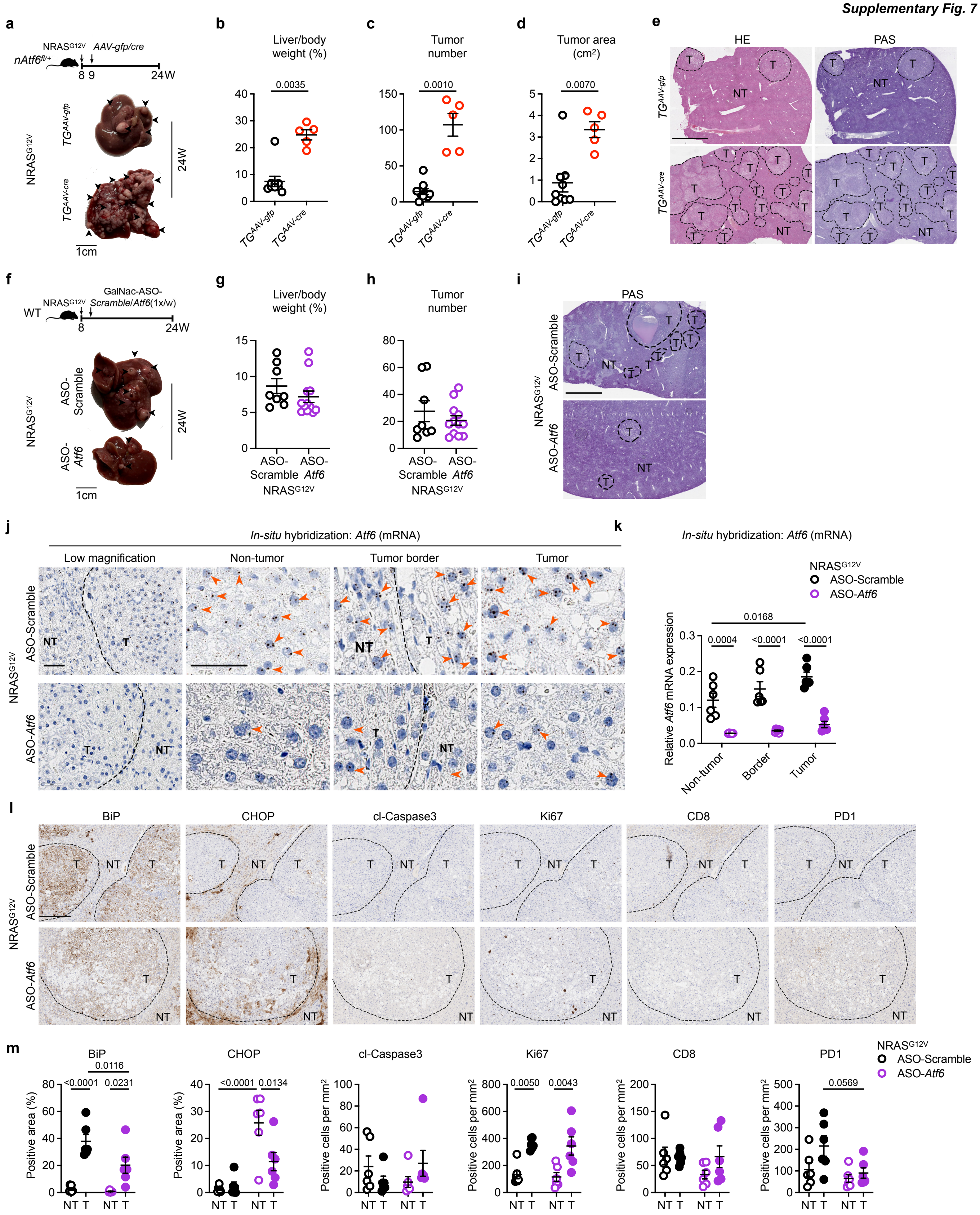

Supplementary Fig. 7: Targeting ATF6α-activation by hepatocyte-delivery of antisense-oligonucleotides (ASO) protects against oncogene-induced HCC, Related to Figure 4.

(a) Scheme of *nAtf6*$^{fl/+}$ mice injected with *AAV8-gfp* or *AAV8-cre* after hydrodynamic tail vein injection (HDTVi) of NRAS^{G12V} plasmid DNA (top), with representative macroscopic liver images (bottom). **(b-d)** Liver-to-body weight **(b)**, tumor number **(c)** and area **(d,** cm^2) per liver of NRAS^{G12V}-injected *TG*$^{AAV\text{-}gfp}$ (n=9) and *TG*$^{AAV\text{-}cre}$ (n=5) mice. **(e)** Representative liver HE and PAS staining of NRAS^{G12V}-injected *TG*$^{AAV\text{-}gfp}$ (n=9) and *TG*$^{AAV\text{-}cre}$ (n=5) mice. Scale bar = 2mm. **(f)** Scheme of C57BL6/J mice (WT) receiving GalNac-conjugated ASO-Scramble or ASO-*Atf6* (2.5mg/kg/week) following HDTVi of NRAS^{G12V} plasmid DNA (top), with representative macroscopic liver images (bottom). **(g-h)** Liver-to-body weight (**g**) and tumor number per liver (**h**) of NRAS^{G12V}-injected WT mice treated with ASO-Scramble (n=8) or ASO-*Atf6* (n=12). **(i)** Representative liver PAS staining of NRAS^{G12V}-injected WT mice treated with ASO-Scramble (n=8) or ASO-*Atf6* (n=12). **(j-k)** Representative *In-situ* hybridization (ISH) for *Atf6* mRNA (**j,** scale bar = 50µm), with quantification (**k**) in non-tumor liver, tumor border and tumor of NRAS^{G12V}-injected WT mice treated with ASO-Scramble (n=6) or ASO-*Atf6* (n=6). **(l-m)** Representative IHC for BiP, CHOP, cleaved-Caspase 3, Ki67, CD8, and PD-1 (**l**) with quantification (**m**) from livers of NRAS^{G12V}-injected WT mice treated with GalNac-conjugated ASO-*Scramble* (n=6) or ASO-*Atf6* (n=6). Scale bar = 300µm. Scatter dot plot data are presented as mean values ± SEM. Data in 7b-d,g,h were analyzed by two-tailed unpaired t-test or Mann-Whitney test based on data normality distribution. Data in 7k,m were analyzed by two-way ANOVA and Tukey's post hoc test. NT: non-tumor, T: tumor (within dotted lines). Mouse icons were created in BioRender. Heikenwälder, M. (2026) https://BioRender.com/lgjnsy9.

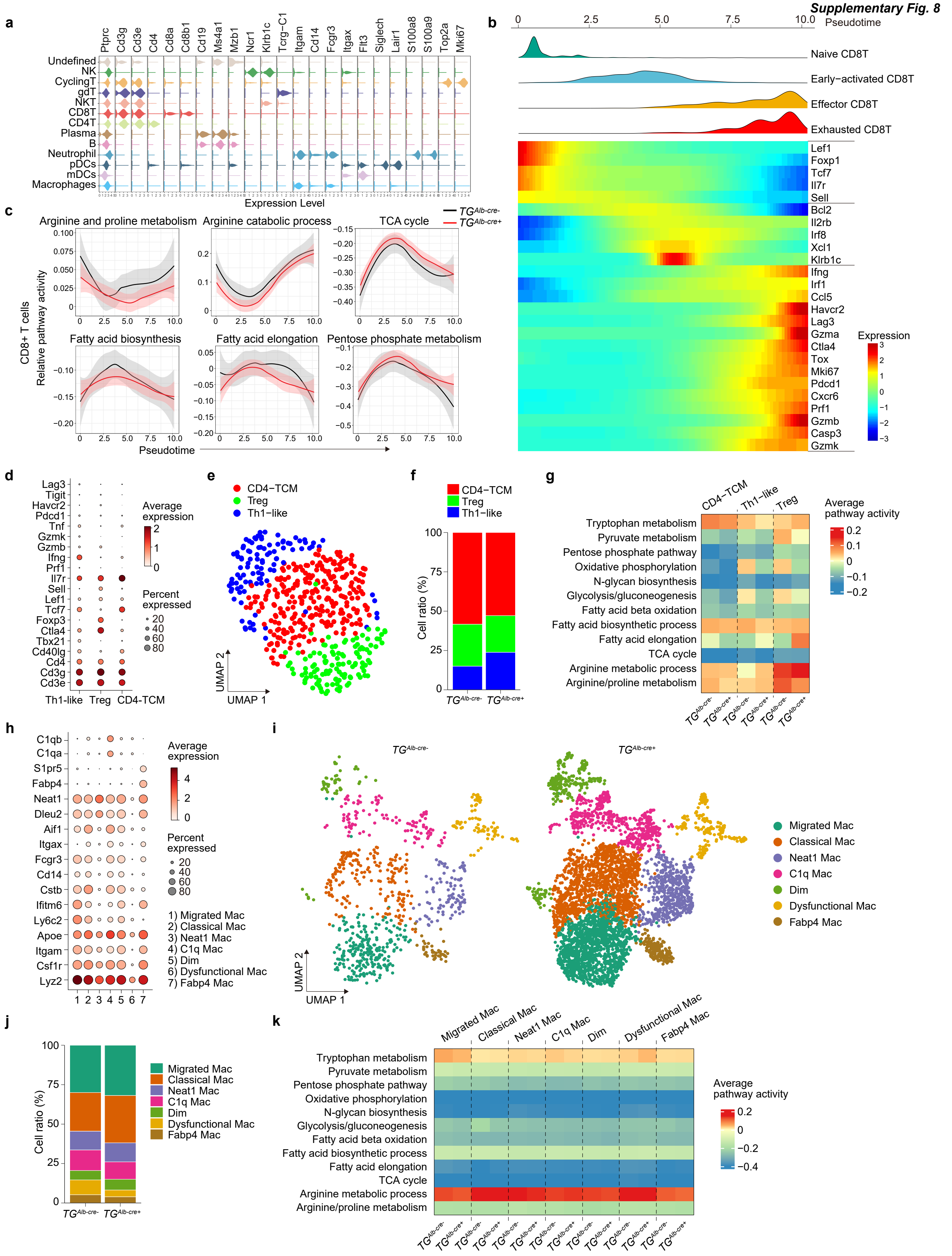
a
Ptprc
Cd3g
Cd3e
Cd4
Cd8a
Cd8b1
Cd19
Ms4a1
Mzb1
Ncr1
Klrb1c
Tcrg−C1
Itgam
Cd14
Fcgr3
Itgax
Flt3
Siglech
Lair1
S100a8
S100a9
Top2a
Mki67
Undefined
NK
CyclingT
gdT
NKT
CD8T
CD4T
Plasma
B
Neutrophil
pDCs
mDCs
Macrophages
Expression Level
b
Pseudotime
Naive CD8T
Early−activated CD8T
Effector CD8T
Exhausted CD8T
Lef1
Foxp1
Tcf7
Il7r
Sell
Bcl2
Il2rb
Irf8
Xcl1
Klrb1c
Ifng
Irf1
Ccl5
Havcr2
Lag3
Gzma
Ctla4
Tox
Mki67
Pdcd1
Cxcr6
Prf1
Gzmb
Casp3
Gzmk
Expression
c
TG^Alb-cre-
TG^Alb-cre+
Arginine and proline metabolism
Arginine catabolic process
TCA cycle
Fatty acid biosynthesis
Fatty acid elongation
Pentose phosphate metabolism
CD8+ T cells
Relative pathway activity
Pseudotime
d
Lag3
Tigit
Havcr2
Pdcd1
Tnf
Gzmk
Gzmb
Ifng
Prf1
Il7r
Sell
Lef1
Tcf7
Foxp3
Ctla4
Tbx21
Cd40lg
Cd4
Cd3g
Cd3e
Th1-like
Treg
CD4-TCM
Average expression
Percent expressed
e
CD4−TCM
Treg
Th1−like
UMAP 2
UMAP 1
f
CD4−TCM
Treg
Th1−like
Cell ratio (%)
TG^Alb-cre-
TG^Alb-cre+
g
CD4−TCM
Th1−like
Treg
Tryptophan metabolism
Pyruvate metabolism
Pentose phosphate pathway
Oxidative phosphorylation
N-glycan biosynthesis
Glycolysis/gluconeogenesis
Fatty acid beta oxidation
Fatty acid biosynthetic process
Fatty acid elongation
TCA cycle
Arginine metabolic process
Arginine/proline metabolism
Average pathway activity
h
C1qb
C1qa
S1pr5
Fabp4
Neat1
Dleu2
Aif1
Itgax
Fcgr3
Cd14
Cstb
Ifitm6
Ly6c2
Apoe
Itgam
Csf1r
Lyz2
Average expression
Percent expressed
1) Migrated Mac
2) Classical Mac
3) Neat1 Mac
4) C1q Mac
5) Dim
6) Dysfunctional Mac
7) Fabp4 Mac
i
TG^Alb-cre-
TG^Alb-cre+
Migrated Mac
Classical Mac
Neat1 Mac
C1q Mac
Dim
Dysfunctional Mac
Fabp4 Mac
UMAP 2
UMAP 1
j
Cell ratio (%)
Migrated Mac
Classical Mac
Neat1 Mac
C1q Mac
Dim
Dysfunctional Mac
Fabp4 Mac
TG^Alb-cre-
TG^Alb-cre+
k
Migrated Mac
Classical Mac
Neat1 Mac
C1q Mac
Dim
Dysfunctional Mac
Fabp4 Mac
Tryptophan metabolism
Pyruvate metabolism
Pentose phosphate pathway
Oxidative phosphorylation
N-glycan biosynthesis
Glycolysis/gluconeogenesis
Fatty acid beta oxidation
Fatty acid biosynthetic process
Fatty acid elongation
TCA cycle
Arginine metabolic process
Arginine/proline metabolism
Average pathway activity

Supplementary Fig. 8. Characterization of liver immune microenvironment in 6-month-old $TG^{Alb\text{-}cre-}$ and $TG^{Alb\text{-}cre+}$ mice by scRNA-seq, Related to Figure 5.

(a) Transcriptional expression (violin plot) of markers used to identify different cell clusters in scRNA-seq of $CD45^+$ cells isolated from livers of $TG^{Alb\text{-}cre-}$ and $TG^{Alb\text{-}cre+}$ mice. **(b)** Distribution of $CD8^+$ T-cell subtypes (labeled by colors, upper panel) along the pseudotime trajectory and related gene expression (lower panel) by scRNA-seq. **(c)** Metabolic pathway activity of CD8 T-cells in livers of $TG^{Alb\text{-}cre-}$ and $TG^{Alb\text{-}cre+}$ mice by scRNA-seq. **(d-f)** Marker gene expression of subclustered $CD4^+$ T-cells (**d**) with associated UMAP representations (**e**) and respective bar plots of cell type distribution (**f**) from livers of $TG^{Alb\text{-}cre-}$ and $TG^{Alb\text{-}cre+}$ mice. **(g)** Heatmap showing the overview of metabolic preferences in $CD4^+$ T-cells from livers of $TG^{Alb\text{-}cre-}$ and $TG^{Alb\text{-}cre+}$ mice. **(h-j)** Marker gene expression of subclustered myeloid cells (**h**) with associated UMAP representations (**i**) and respective bar plots of cell type distribution (**j**) from livers of $TG^{Alb\text{-}cre-}$ and $TG^{Alb\text{-}cre+}$ mice. **(k)** Plots showing the overview of metabolic preferences in myeloid cells from livers of $TG^{Alb\text{-}cre-}$ and $TG^{Alb\text{-}cre+}$ mice. n=4/group.

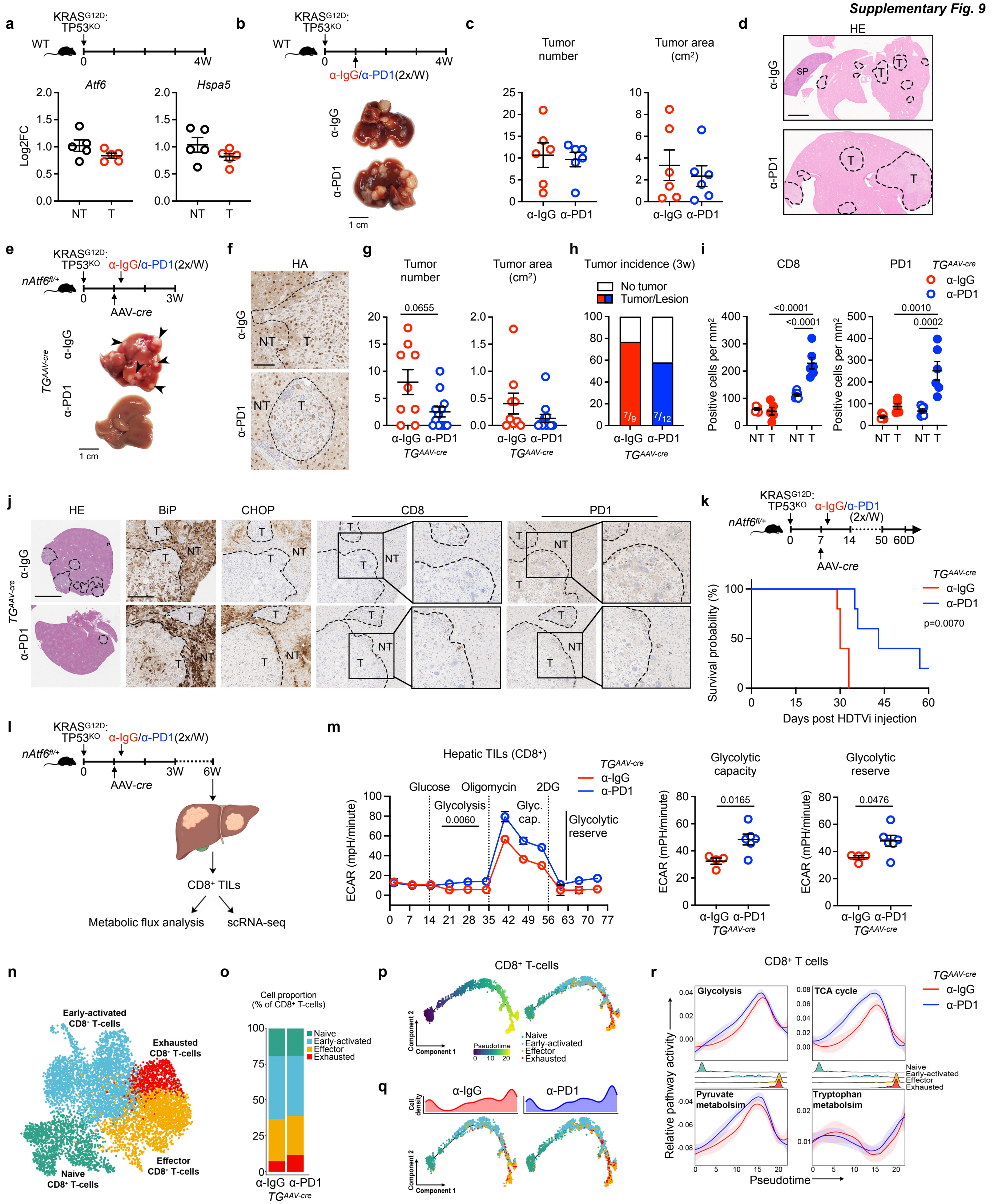
a
KRAS^G12D:TP53^KO
WT
0
4W
Atf6
Hspa5
Log2FC
NT T
b
α-IgG/α-PD1(2x/W)
1 cm
c
Tumor number
Tumor area (cm²)
α-IgG α-PD1
d
HE
SP
T
e
nAtf6fl/+
AAV-cre
3W
TGAAV-cre
f
HA
NT
g
0.0655
h
Tumor incidence (3w)
No tumor
Tumor/Lesion
7/9
7/12
i
CD8
PD1
Positive cells per mm²
<0.0001
0.0010
0.0002
j
BiP
CHOP
k
0 7 14 50 60D
Survival probability (%)
Days post HDTVi injection
p=0.0070
l
6W
CD8+ TILs
Metabolic flux analysis
scRNA-seq
m
Hepatic TILs (CD8+)
Glucose
Oligomycin
2DG
Glycolysis
0.0060
Glyc. cap.
Glycolytic reserve
ECAR (mpH/minute)
Glycolytic capacity
0.0165
0.0476
n
Early-activated CD8+ T-cells
Exhausted CD8+ T-cells
Naive CD8+ T-cells
Effector CD8+ T-cells
o
Cell proportion (% of CD8+ T-cells)
Naive
Early-activated
Effector
Exhausted
p
CD8+ T-cells
Component 1
Component 2
Pseudotime
q
Cell density
r
CD8+ T cells
Glycolysis
TCA cycle
Pyruvate metabolism
Tryptophan metabolsim
Relative pathway activity
Pseudotime

Supplementary Fig. 9: ATF6α-activation sensitizes KRAS^{G12D}:TP53^{KO} liver cancer preclinical model to anti-PD1 monotherapy, Related to Figure 5.

(a) Experimental scheme (top), and qRT-PCR analysis of *Atf6* or *Hspa5* mRNAs in non-tumor (NT) or tumor (T) livers from C57BL6/J (WT) mice (n=5) after HDTVi of KRAS^{G12D}:TP53^{KO} plasmid DNA. **(b)** Scheme of WT mice treated with anti-IgG (α-IgG, n=6) or anti-PD1 (α-PD1, n=6) after HDTVi of KRAS^{G12D}:TP53^{KO} plasmid DNA (top) with representative, macroscopic liver images at sacrifice (bottom). **(c)** Tumor number (left) and area (right, cm^2) per liver from α-IgG/PD1-treated WT mice after HDTVi of KRAS^{G12D}:TP53^{KO} plasmid DNA (n=6/group). **(d)** Representative HE staining of livers from α-IgG/PD1-treated WT mice after HDTVi of KRAS^{G12D}:TP53^{KO} plasmid DNA. Scale bar = 3mm. **(e)** Scheme of *nATF6$^{fl/+}$* mice administered AAV8-*cre* and treated with α-IgG/PD1 antibodies after HDTVi (KRAS^{G12D}:TP53^{KO}, top), with representative macroscopic liver images at sacrifice (bottom). **(f)** Representative liver IHC of HA from α-IgG/PD1-treated *nATF6$^{fl/+}$* mice after HDTVi of KRAS^{G12D}:TP53^{KO} plasmid DNA, (scale bar = 100µm). **(g)** Tumor number (left) and area (right, cm^2) per liver of KRAS^{G12D}:TP53^{KO}-injected *TG$^{AAV-cre}$* mice treated with α-IgG (n=9) or α-PD1 (n=12) antibodies. **(h)** Tumor incidence of KRAS^{G12D}:TP53^{KO}-injected *TG$^{AAV-cre}$* mice treated with α-IgG (n=9) or α-PD1 (n=12) antibodies. **(i-j)** Representative liver HE staining and IHC for BiP, CHOP, HA, CD8, and PD1 (**j**) in livers of KRAS^{G12D}:TP53^{KO}-injected *TG$^{AAV-cre}$* mice treated with α-IgG (n=5) or α-PD1 (n=6) antibodies, with quantification of CD8 and PD1 (**i**). Scale bar = 3mm (HE), scale bar = 200µm (IHC). **(k)** Experimental scheme (top) and Kaplan-Meier survival curves (bottom) of *TG$^{AAV-cre}$* mice treated with α-IgG (n=5) or α-PD1 (n=5) after HDTVi of KRAS^{G12D}:TP53^{KO} plasmid DNA. **(l)** Scheme of tumor-infiltrating lymphocytes (TILs) harvested from liver tumors of *TG$^{AAV-cre}$* mice treated with α-IgG/PD1 after HDTVi of KRAS^{G12D}:TP53^{KO} plasmid DNA, followed by metabolic flux analysis (seahorse bioanalyzer) and scRNA-seq analysis. **(m)** Metabolic flux analysis (left) and quantification (right) for extracellular acidification rates (ECAR) and associated glycolytic capacity and glycolytic reserve in TILs from α-IgG (n=4) or α-PD1 (n=6) treated KRAS^{G12D}:TP53^{KO} *TG$^{AAV-cre}$* mice. **(n-o)** scRNA-seq UMAP representations of subclustered CD8$^+$ T-cells (**n**) with respective cell-type distribution (**o**) from liver tumors of α-IgG or α-PD1 treated KRAS^{G12D}:TP53^{KO} *TG$^{AAV-cre}$* mice (n=3/group). **(p)** Trajectory analysis of CD8$^+$ T-cells from livers of α-IgG/PD1 treated KRAS^{G12D}:TP53^{KO} *TG$^{AAV-cre}$* mice, with pseudotime ordering (left) predicting CD8$^+$ T-cell

development by subtype (right). **(q)** Density distribution of TILs by state (top) and pseudotime-ordered TILs (bottom) from livers of α-IgG/PD1 treated $KRAS^{G12D}$:$TP53^{KO}$ $TG^{AAV\text{-}cre}$ mice. **(r)** Metabolic pathway activity of TILs by pseudotime in livers of α-IgG/PD1 treated $KRAS^{G12D}$:$TP53^{KO}$ $TG^{AAV\text{-}cre}$ mice. Scatter dot plot and line graph data are presented as mean values ± SEM. Data in 9a,c,g,m were analyzed by two-tailed unpaired t-test or Mann-Whitney test based on data normality distribution. Data in 9i were analyzed by two-way ANOVA and Tukey's post hoc test. Data in 9k were analyzed by log-rank (Mantel-Cox) test. NT: non-tumor, T: tumor (within dotted lines). Mouse icons and liver schematic were created in BioRender. Heikenwälder, M. (2026) https://BioRender.com/lgjnsy9.

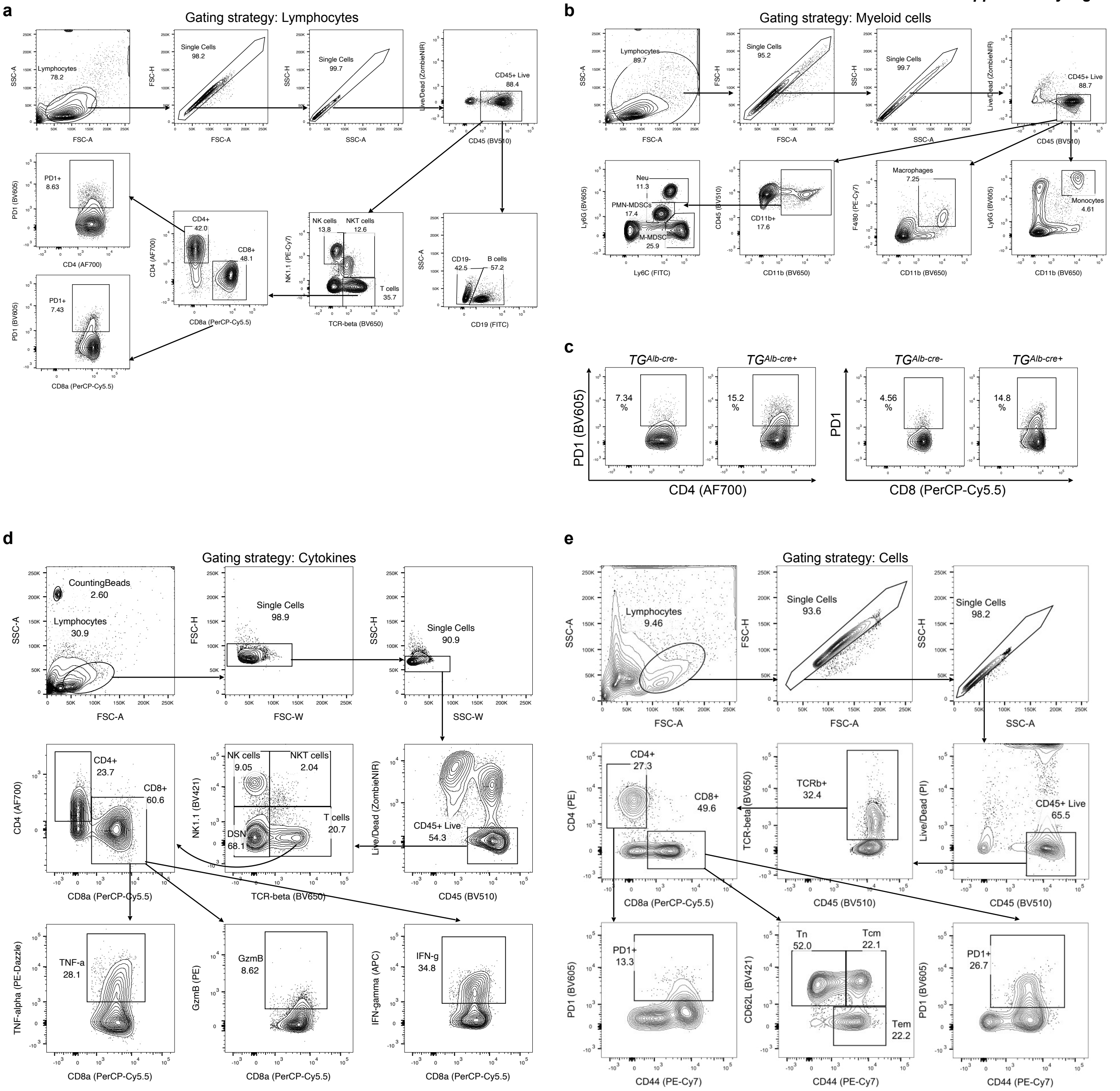
a
Gating strategy: Lymphocytes
Lymphocytes 78.2
Single Cells 98.2
Single Cells 99.7
CD45+ Live 88.4
PD1+ 8.63
CD4+ 42.0
CD8+ 48.1
NK cells 13.8
NKT cells 12.6
T cells 35.7
CD19- 42.5
B cells 57.2
PD1+ 7.43
b
Gating strategy: Myeloid cells
Lymphocytes 89.7
Single Cells 95.2
Single Cells 99.7
CD45+ Live 88.7
Neu 11.3
PMN-MDSCs 17.4
M-MDSC 25.9
CD11b+ 17.6
Macrophages 7.25
Monocytes 4.61
c
TGAlb-cre-
TGAlb-cre+
7.34 %
15.2 %
4.56 %
14.8 %
PD1 (BV605)
CD4 (AF700)
PD1
CD8 (PerCP-Cy5.5)
d
Gating strategy: Cytokines
CountingBeads 2.60
Lymphocytes 30.9
Single Cells 98.9
Single Cells 90.9
CD4+ 23.7
CD8+ 60.6
NK cells 9.05
NKT cells 2.04
T cells 20.7
DSN 68.1
CD45+ Live 54.3
TNF-a 28.1
GzmB 8.62
IFN-g 34.8
e
Gating strategy: Cells
Lymphocytes 9.46
Single Cells 93.6
Single Cells 98.2
CD4+ 27.3
CD8+ 49.6
TCRb+ 32.4
CD45+ Live 65.5
PD1+ 13.3
Tn 52.0
Tcm 22.1
Tem 22.2
PD1+ 26.7

Supplementary Fig. 10: FACS gating strategies.

(a-b) Gating strategy for lymphocytes (**a**) or myeloid cells (**b**) from livers of $TG^{Alb\text{-}cre+}$ and $TG^{Alb\text{-}cre-}$ mice by FACS, Related to **Figure 5**, and **Extended Data Figure 9. (c)** Representative FACS scatter plots showing $CD4^{+}PD1^{+}$ and $CD8^{+}PD1^{+}$ T-cells in livers of 6-month-old $TG^{Alb\text{-}cre-}$ and $TG^{Alb\text{-}cre+}$ mice. Related to **Figure 5**. **(d-e)** Gating strategy for cytokine-producing cells (**d**) or lymphocytes (**e**) from livers of 9-month-old $TG^{Alb\text{-}cre+}$ and $TG{:}Pdcd1^{-/-}$ mice (n=4/group). Related to **Figure 5**, and **Extended Data Figure 12**.

Supplementary Fig. 11a

As shown in Main Fig. 1f

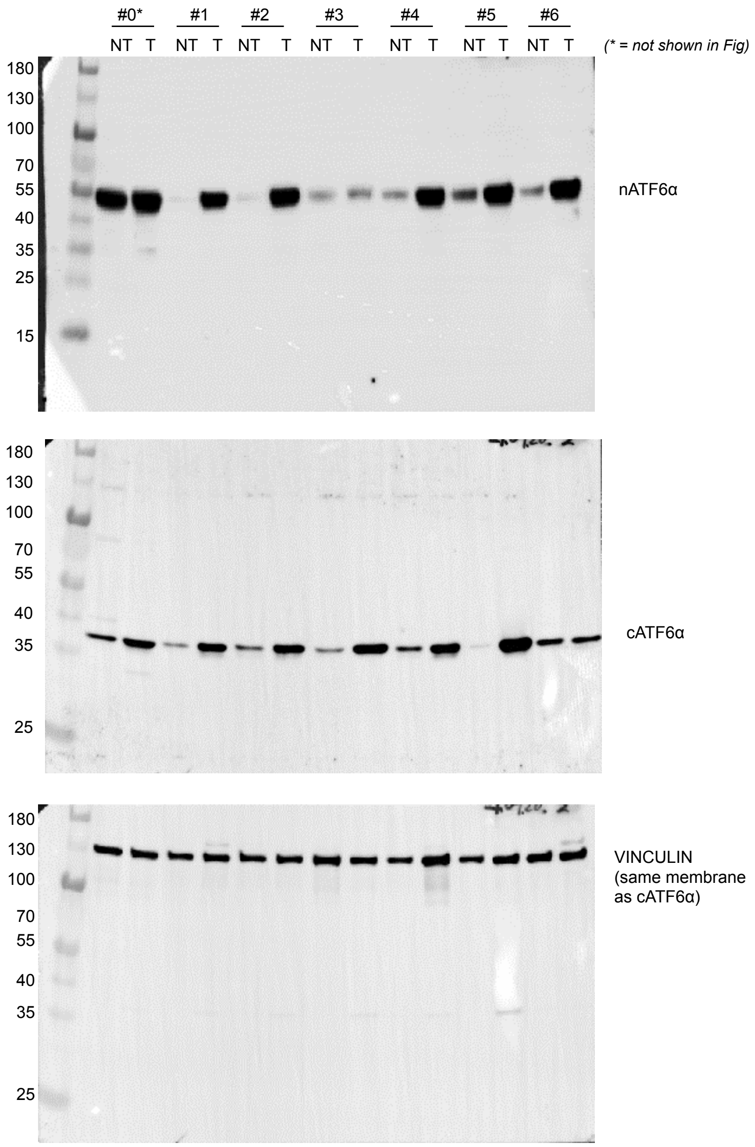

PRIMARY ANTIBODY TARGET	SOURCE	IDENTIFIER
ATF6α	Enzo	ADI-905-729-100
ATF6α	Signalway Antibody	32008
VINCULIN	Santa Cruz	sc-73614

Supplementary Fig. 11b

As shown in Main Fig. 2e

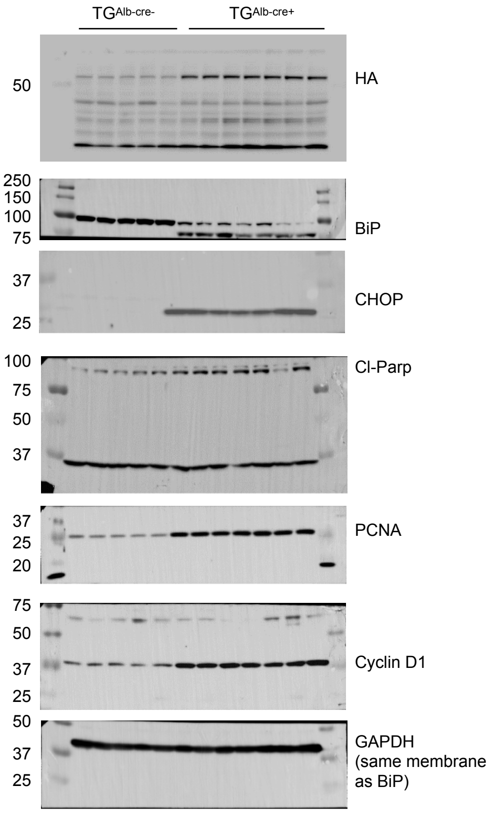

PRIMARY ANTIBODY TARGET	SOURCE	IDENTIFIER
HA	Abcam	9110
BiP	Cell Signaling Technology	3177
CHOP	Cell Signaling Technology	5554
PARP	Cell Signaling Technology	9532
PCNA	Cell Signaling Technology	13110
CyclinD1	Cell Signaling Technology	2978
GAPDH	Cell Signaling Technology	2118

As shown in Main Fig. 2n

Supplementary Fig. 11c

PRIMARY ANTIBODY TARGET	SOURCE	IDENTIFIER
VINCULIN	Sigma	V9131
TRAPα	Abcam	ab133238
ATF6α	Enzo	ADI-905-729-100
CHOP	Santa Cruz	sc-7351
FBP1	Sigma	HPA005857
KDEL ER marker (BiP and GRP94)	Santa Cruz	sc-58774

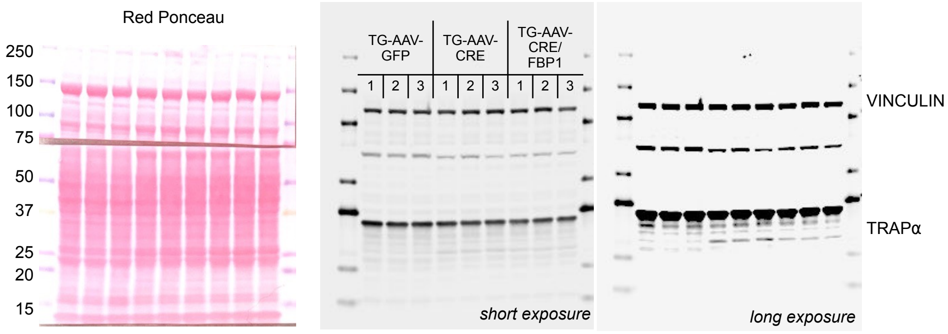

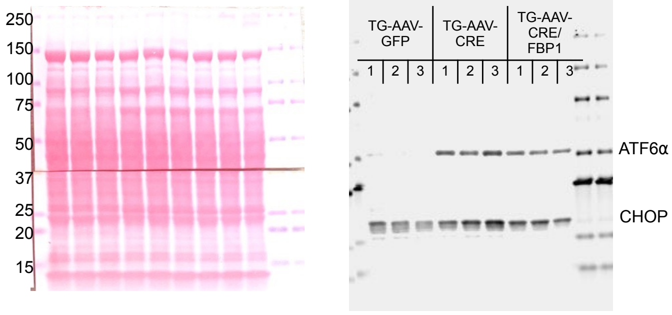

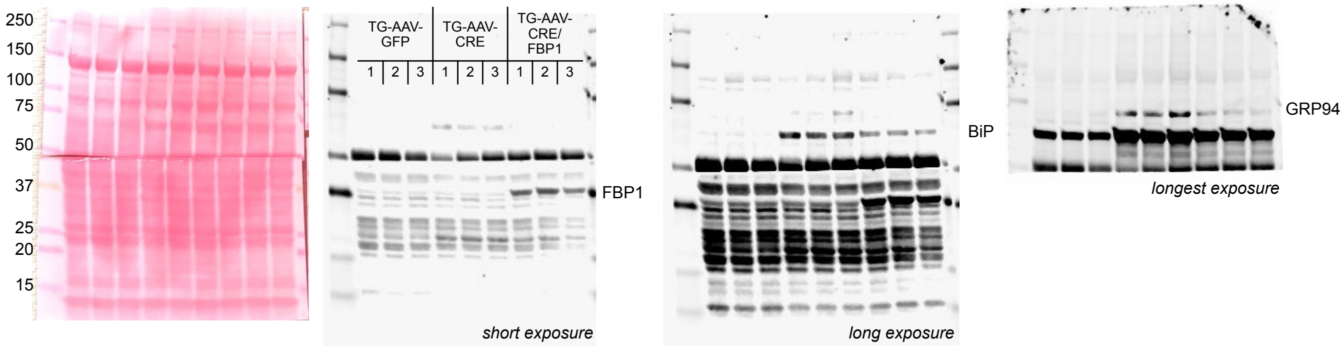

Supplementary Fig. 11d

As shown in Extended Fig. 2c

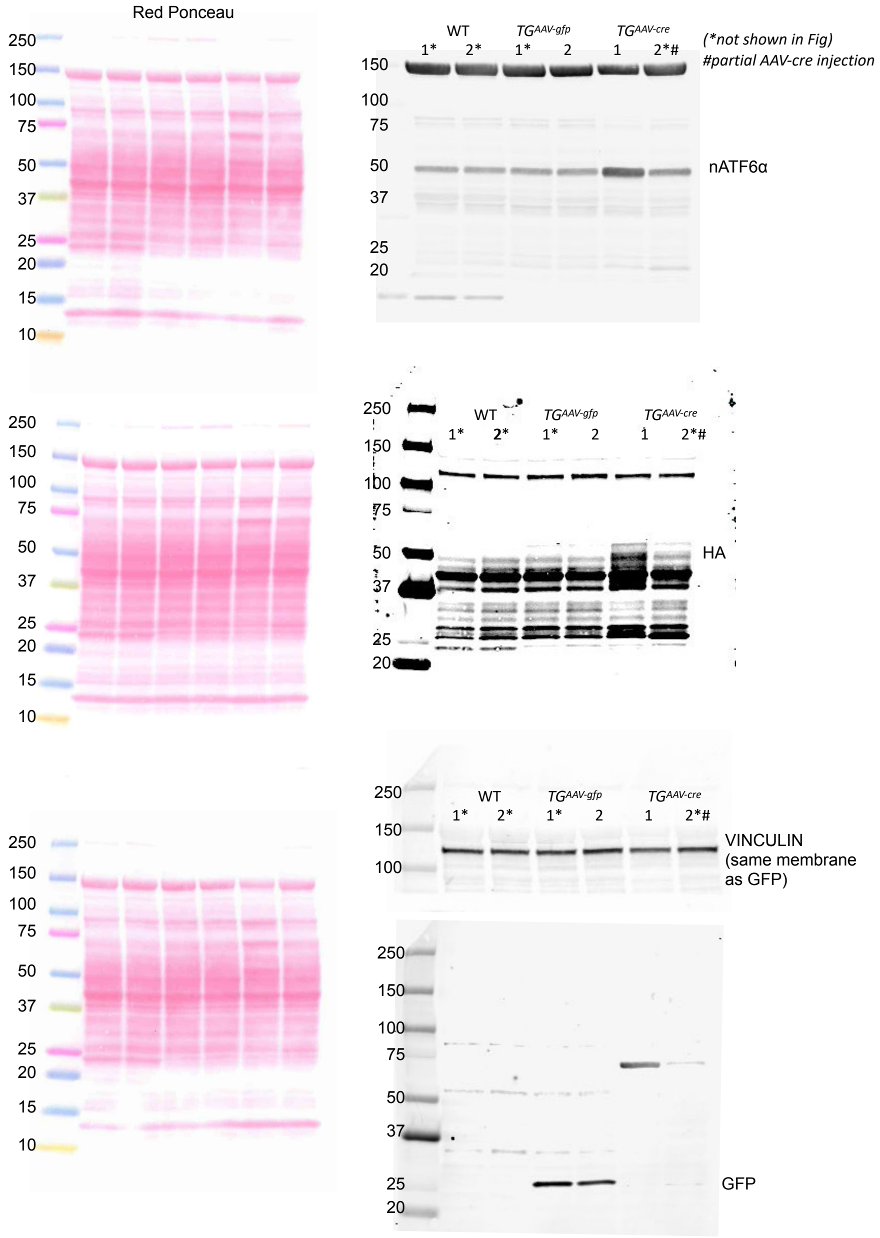

PRIMARY ANTIBODY TARGET	SOURCE	IDENTIFIER
ATF6α	Enzo	ADI-905-729-100
HA	Abcam	9110
VINCULIN	Sigma	V9131
GFP	Novus Biologicals	NB600-308

As shown in Extended Fig. 3l

Supplementary Fig. 11e

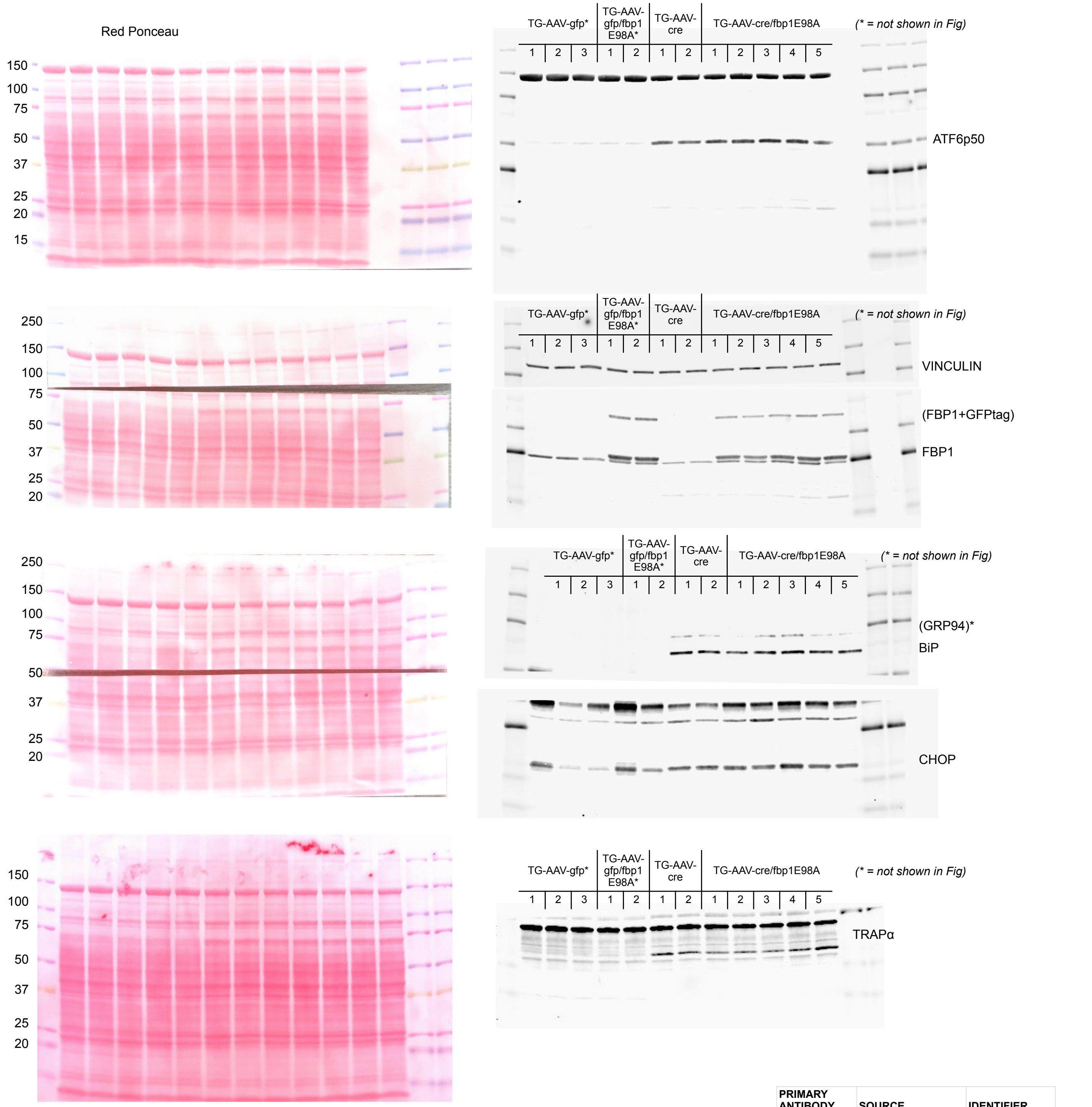

PRIMARY ANTIBODY TARGET	SOURCE	IDENTIFIER
ATF6α	Enzo	ADI-905-729-100
VINCULIN	Sigma	V9131
FBP1	Sigma	HPA005857
KDEL ER marker (BiP and GRP94)	Santa Cruz	sc-58774
CHOP	Santa Cruz	sc-7351
TRAPα	Abcam	ab133238

Supplementary Fig. 11f

PRIMARY ANTIBODY TARGET	SOURCE	IDENTIFIER
ATF6α	OriGene	TA336753
VINCULIN	Santa Cruz	sc-73614

As shown in Extended Fig. 4f

1-5 WT liver
6, 8, 10, 12 *TG*$^{Alb\text{-}cre}$ non-tumor liver
7, 9, 11, 13 *TG*$^{Alb\text{-}cre}$ tumor

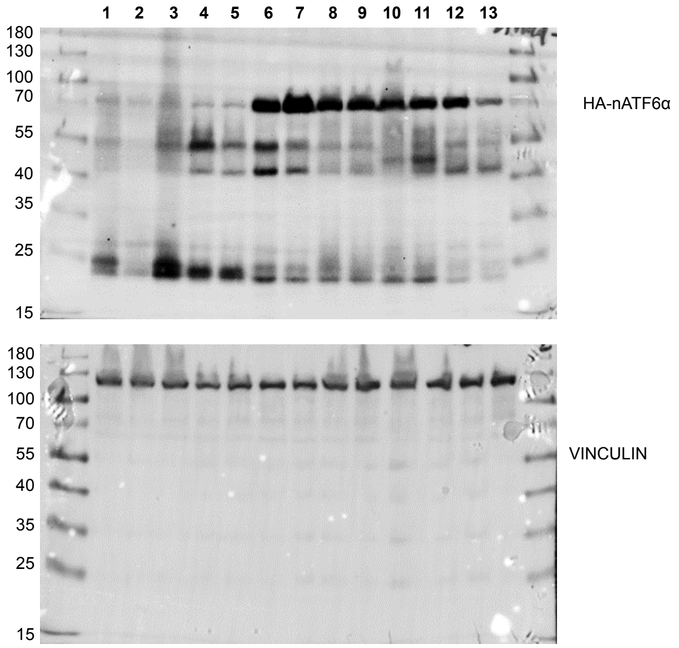

As shown in Extended Fig. 4h

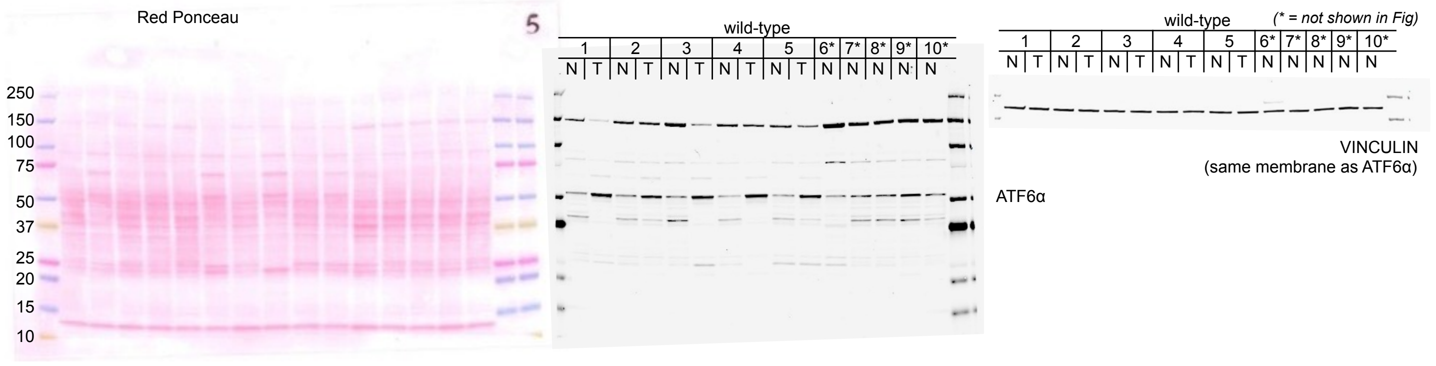

PRIMARY ANTIBODY TARGET	SOURCE	IDENTIFIER
ATF6α	Enzo	ADI-905-729-100
VINCULIN	Sigma	V9131

Supplementary Fig. 11h

As shown in Extended Fig. 5h

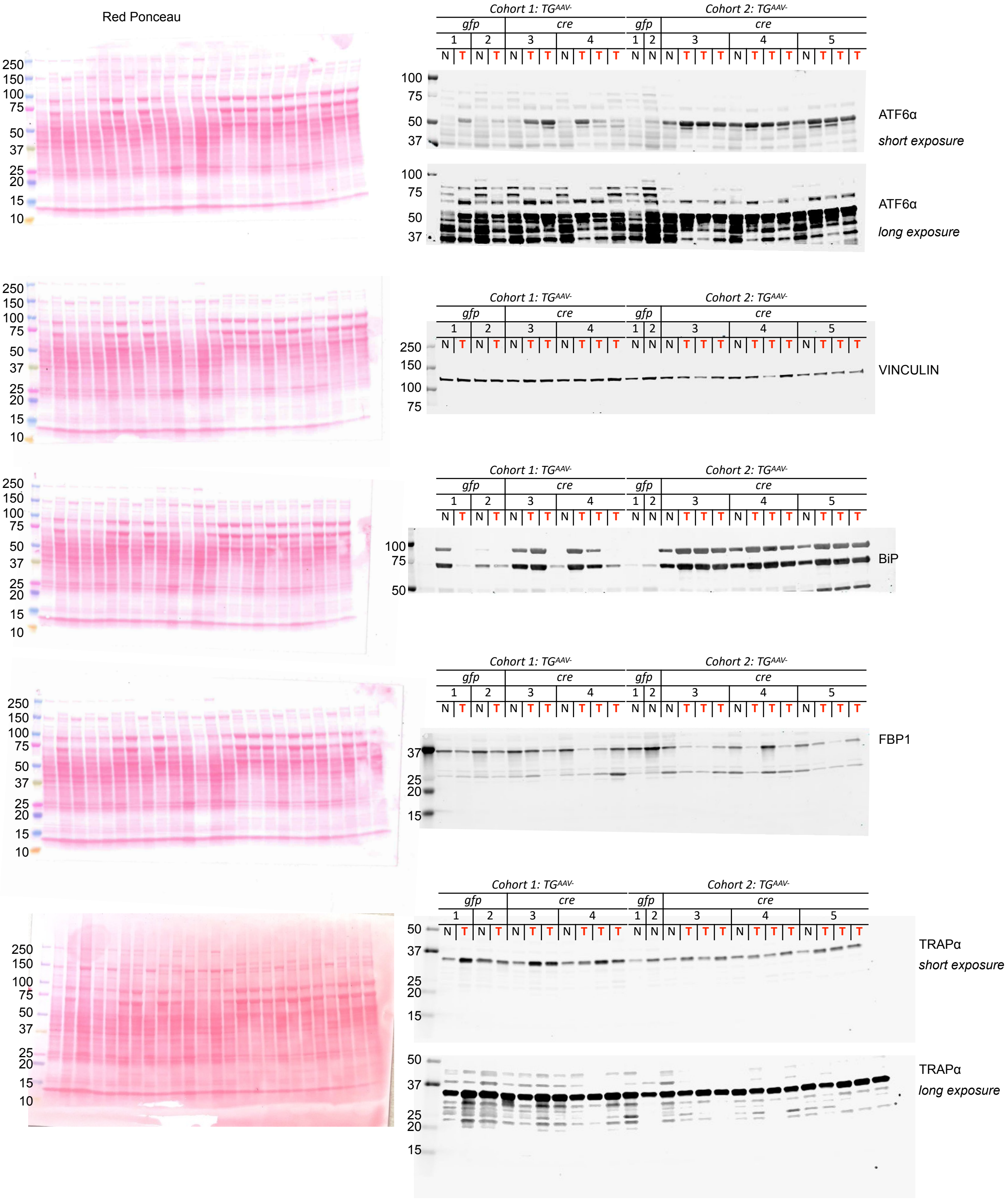

PRIMARY ANTIBODY TARGET	SOURCE	IDENTIFIER
ATF6α	Enzo	ADI-905-729-100
VINCULIN	Sigma	V9131
KDEL ER marker (BiP and GRP94)	Santa Cruz	sc-58774
FBP1	Sigma	HPA005857
TRAPα	Abcam	ab133238

Supplementary Fig. 11i

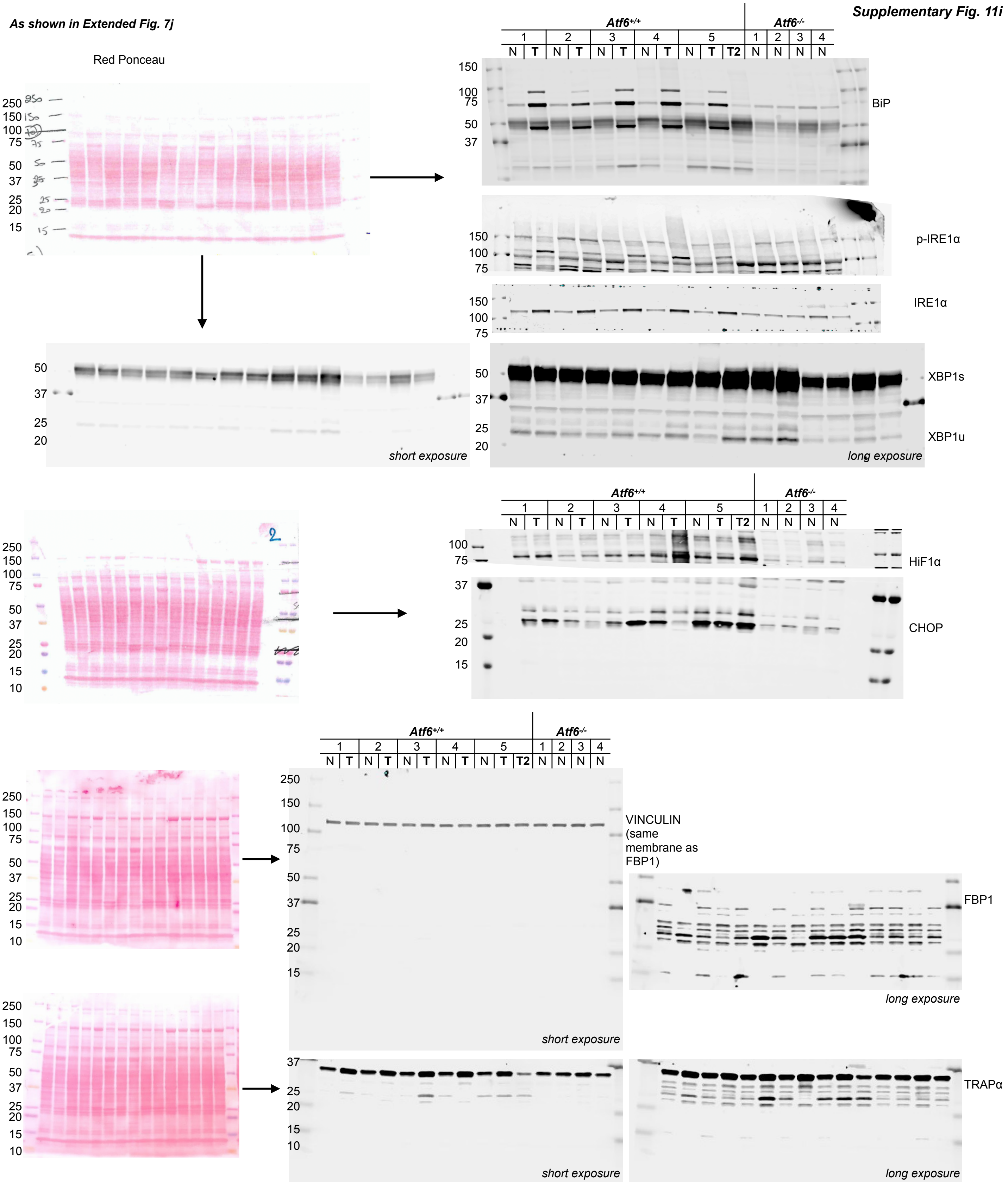

PRIMARY ANTIBODY TARGET	SOURCE	IDENTIFIER
KDEL ER marker (BiP and GRP94)	Santa Cruz	sc-58774
p-IRE1α	This paper	This paper
IRE1α	Cell Signaling Technology	3294
XBP-1	Santa Cruz	sc-8015
HIF-1 alpha	Novus Biologicals	NB100-105
CHOP	Santa Cruz	sc-7351
VINCULIN	Sigma	V9131
FBP1	Sigma	HPA005857
TRAPα	Abcam	ab133238

As shown in Extended Fig. 9c

Supplementary Fig. 11j

PRIMARY ANTIBODY TARGET	SOURCE	IDENTIFIER
PD-L1	Cell Signaling Technology	64988
GAPDH	Cell Signaling Technology	2118

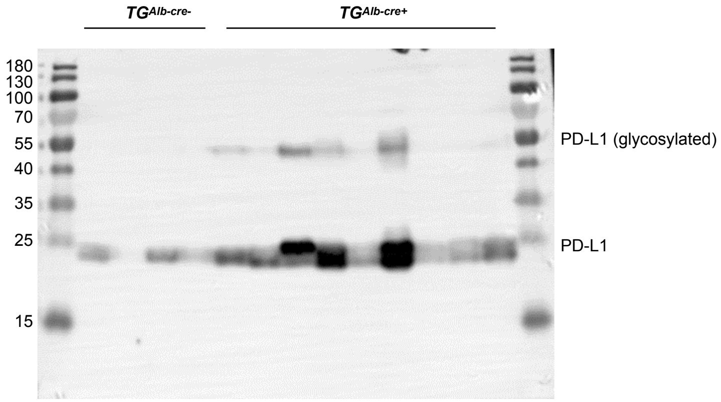

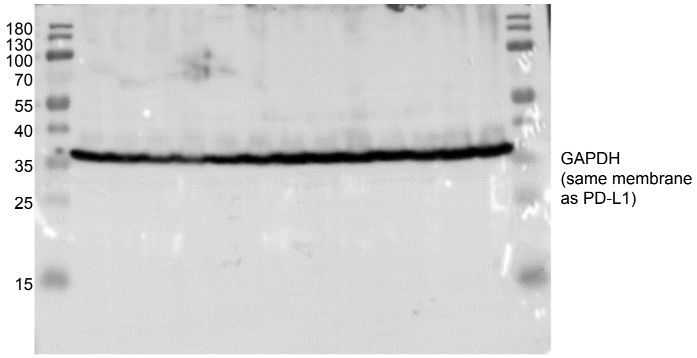

As shown in Supplementary Fig. 1c

Supplementary Fig. 11k

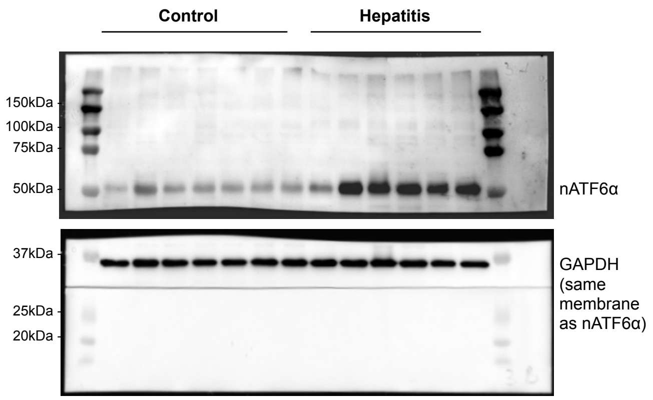

PRIMARY ANTIBODY TARGET	SOURCE	IDENTIFIER
ATF6α	Enzo	ADI-905-729-100
GAPDH	Cell Signaling Technology	2118

Supplementary Fig. 11l

As shown in Supplementary Fig. 3e

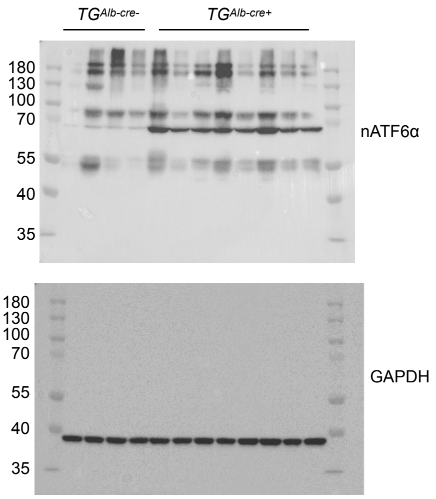

PRIMARY ANTIBODY TARGET	SOURCE	IDENTIFIER
ATF6α	OriGene	TA336753
GAPDH	Cell Signaling Technology	2118

Supplementary Fig. 11m

PRIMARY ANTIBODY TARGET	SOURCE	IDENTIFIER
ATF6α	Enzo	ADI-905-729-100
CHOP	Santa Cruz	sc-7351
KDEL ER marker (BiP and GRP94)	Santa Cruz	sc-58774
FBP1	Sigma	HPA005857
VINCULIN	Sigma	V9131
XBP-1	Santa Cruz	sc-8015
ATF4	Cell Signalling	11815
TRAPα	Abcam	ab133238

As shown in Supplementary Fig. 4m

Red Ponceau

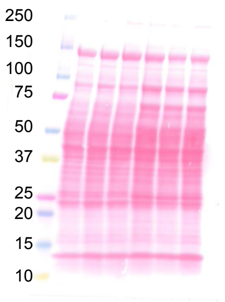

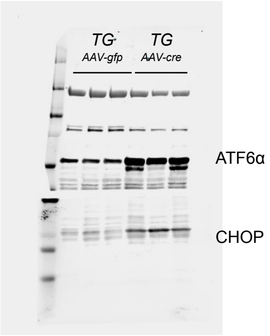

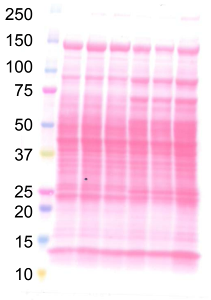

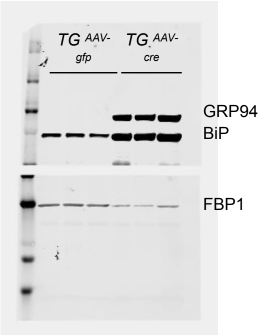

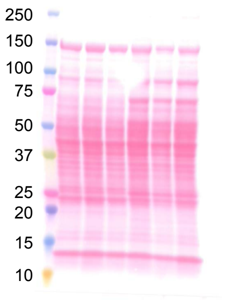

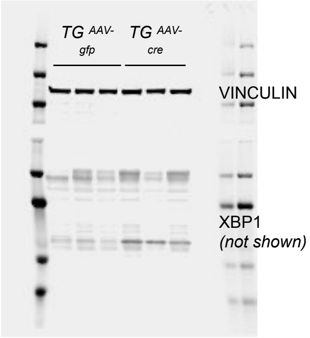

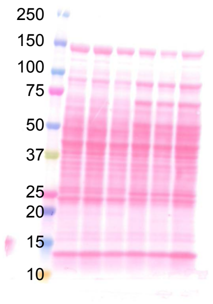

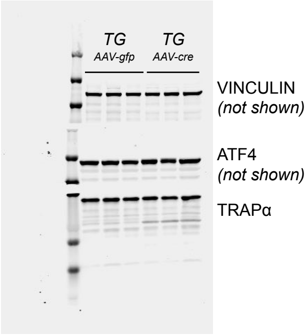

Supplementary Fig. 11a-m. Original/uncropped immunoblots.

Supplementary Table 1:
Nomenclature and description of breeding schemes or injections for ATF6α mouse models

Nomenclature	Description
nAtf6$^{fl/fl}$	R26-LSL-nATF6α-HA, homozygote
TG$^{Alb\text{-}cre-}$	Control, *nAtf6*$^{fl/fl}$ x Albumin Cre cross, Cre-
TG$^{Alb\text{-}cre+}$	*nAtf6*$^{fl/fl}$ x Albumin Cre cross, Cre+
TG$^{AAV\text{-}gfp}$	Control, *nAtf6*$^{fl/+}$ tail vein injected with AAV8-GFP
TG$^{AAV\text{-}cre}$	*nAtf6*$^{fl/+}$ tail vein injected with AAV8-CRE
TG$^{AAV\text{-}cre/fbp1}$	*nAtf6*$^{fl/+}$ tail vein injected with AAV8-CRE and AAV8-FBP1 (catalytically active)
TG$^{AAV\text{-}cre/fbp1E98A}$	*nAtf6*$^{fl/+}$ tail vein injected with AAV8-CRE and AAV8-FBP1^{E98A} (catalytically inactive mutant)
TG:Pdcd1$^{-/-}$	*TG*$^{Alb\text{-}cre+}$ x *Pdcd1* KO cross
Atf6$^{+/+}$	Control, *Atf6* KO breeding line
Atf6$^{-/-}$	*Atf6* KO
Atf6$^{fl/fl}$	Control, *Atf6* liver-specific KO breeding line
Atf6$^{\Delta Hep}$	*Atf6* liver-specific KO
MUP-uPA	uPA transgenic mice under the control of the mature hepatocyte-specific promoter for MUP
Atf6$^{+/+}$*:MUP-uPA*	*Atf6*$^{+/+}$ crossed with *MUP-uPA*
Atf6$^{-/-}$*:MUP-uPA*	*Atf6*$^{-/-}$ crossed with *MUP-uPA*
ASO-*Scramble*	Control, mice injected with GalNac-ASO-*Scramble*
ASO-*Atf6*	Mice injected with GalNac-ASO-*Atf6*

Supplementary Table 2: Human sample details *Li, Lebeaupin et al.*

patient ID	time point	age	sex	etiology	comorbidities	histologic specifications	treatment	site	IMC	nanostring
HCC#1	primary	59	f	trHCV	/	/	Baseline	liver	no	no
HCC#1	primary	59	f	trHCV	/	/	Baseline	tumor	no	no
HCC#1	recidive	63	f	trHCV	/	/	after resection and TACE of primary HCC	liver	no	no
HCC#1	recidive	63	f	trHCV	/	/	after resection and TACE of primary HCC	margin	yes	no
HCC#2	recidive	57	f	trHCV	HIV (controlled)	/	after resection of primary HCC	liver	no	no
HCC#2	recidive	57	f	trHCV	HIV (controlled)	/	after resection of primary HCC	margin	no	no
HCC#2	recidive	57	f	trHCV	HIV (controlled)	/	after resection of primary HCC	tumor	no	no
HCC#3	primary	60	m	MASH	/	/	Baseline	margin	no	no
HCC#3	primary	60	m	MASH	/	/	Baseline	tumor	no	no
HCC#3	recidive	64	m	MASH	/	/	after resection of primary HCC	liver	no	no
HCC#3	recidive	64	m	MASH	/	/	after resection of primary HCC	margin	yes	no
HCC#3	recidive	64	m	MASH	/	/	after resection of primary HCC	tumor	no	yes
HCC#4	primary	37	m	cHBV	/	/	Baseline	liver	yes	yes
HCC#4	primary	37	m	cHBV	/	/	Baseline	margin	yes	no
HCC#4	primary	37	m	cHBV	/	/	Baseline	tumor	yes	yes
HCC#5	primary	52	m	trHCV, steatosis hepatitis	/	/	Baseline	liver	yes	yes
HCC#5	primary	52	m	trHCV, steatosis hepatitis	/	/	Baseline	margin	yes	no
HCC#5	primary	52	m	trHCV, steatosis hepatitis	/	/	Baseline	tumor	yes	yes
HCC#6	primary	70	m	ASH	/	/	Baseline	tumor	no	no
HCC#7	primary	81	m	a.e. ASH	/	/	Baseline	tumor	no	no
HCC#8	primary	60	m	cHCV	/	/	Baseline	liver	no	yes
HCC#8	primary	60	m	cHCV	/	/	Baseline	margin	yes	no
HCC#8	primary	60	m	cHCV	/	/	Baseline	tumor	no	yes
HCC#9	primary	76	m	steatosis hepatitis, a.e. MASH	RCC FD 2016	/	after neoadjuvant TACE	liver	no	yes
HCC#9	primary	76	m	steatosis hepatitis, a.e. MASH	RCC FD 2016	/	after neoadjuvant TACE	margin	no	no
HCC#9	primary	76	m	steatosis hepatitis, a.e. MASH	RCC FD 2016	/	after neoadjuvant TACE	tumor	no	yes
HCC#9	recidive	79	m	steatosis hepatitis, a.e. MASH	RCC FD 2016	/	after neoadjuvant TACE and resection of primary HCC	margin	yes	no
HCC#9	recidive	79	m	steatosis hepatitis, a.e. MASH	RCC FD 2016	/	Baseline	tumor	no	no
HCC#10	primary	68	m	ASH	/	/	Baseline	liver	no	yes
HCC#10	primary	68	m	ASH	/	/	Baseline	margin	no	no
HCC#10	primary	68	m	ASH	/	/	Baseline	tumor	no	yes
HCC#11	primary	63	m	cHBV	NTX 02/2019 (IgA nephritis)	/	Baseline	liver	no	yes
HCC#11	primary	63	m	cHBV	NTX 02/2019 (IgA nephritis)	/	Baseline	margin	no	no
HCC#11	primary	63	m	cHBV	NTX 02/2019 (IgA nephritis)	/	Baseline	tumor	no	yes
HCC#12	primary	71	m	HBV	/	/	Baseline	liver	no	no
HCC#12	primary	71	m	HBV	/	/	Baseline	margin	no	no
HCC#12	primary	71	m	HBV	/	/	Baseline	tumor	no	no
HCC#13	primary	53	f	n.d.	/	HCC from adenoma	Baseline	liver	no	no
HCC#13	primary	53	f	n.d.	/	HCC from adenoma	Baseline	margin	no	no
HCC#13	primary	53	f	n.d.	/	HCC from adenoma	Baseline	tumor	no	no
HCC#14	primary	66	m	ASH	/	/	Baseline	liver	no	yes
HCC#14	primary	66	m	ASH	/	/	Baseline	margin	yes	no
HCC#14	primary	66	m	ASH	/	/	Baseline	tumor	no	yes
HCC#15	primary	57	m	trHCV	HIV (controlled)	/	Baseline	liver	no	no
HCC#15	primary	57	m	trHCV	HIV (controlled)	/	Baseline	tumor	no	no
HCC#15	recidive	59	m	trHCV	HIV (controlled)	/	after resection of primary HCC	liver	no	no
HCC#15	recidive	59	m	trHCV	HIV (controlled)	/	after resection of primary HCC	margin	no	no
HCC#15	recidive	59	m	trHCV	HIV (controlled)	/	after resection of primary HCC	tumor	no	yes
HCC#16	primary	69	f	cHCV	/	/	Baseline	liver	no	no
HCC#16	primary	69	f	cHCV	/	/	Baseline	margin	no	no
HCC#17	primary	53	m	cHCV	/	/	Baseline	liver	no	yes
HCC#17	primary	53	m	cHCV	/	/	Baseline	margin	no	no
HCC#17	primary	53	m	cHCV	/	/	Baseline	tumor	no	yes
HCC#18	primary	57	m	cHCV, ASH	/	/	Baseline	liver	no	no
HCC#19	primary	75	m	ASH	/	/	Baseline	liver	no	yes
HCC#19	primary	75	m	ASH	/	/	Baseline	margin	no	no
HCC#19	primary	75	m	ASH	/	/	Baseline	tumor	no	yes
HCC#20	primary	70	m	ASH	/	/	Baseline	margin	no	no
HCC#21	primary	87	m	steatosis a.e. MASH	/	/	Baseline	liver	yes	no
HCC#21	primary	87	m	steatosis a.e. MASH	/	/	Baseline	margin	yes	no
HCC#21	primary	87	m	steatosis a.e. MASH	/	/	Baseline	tumor	yes	no
HCC#22	primary	76	m	ASH	/	/	Baseline	liver	no	yes
HCC#22	primary	76	m	ASH	/	/	Baseline	margin	yes	no

HCC#22	primary	76	m	ASH	/	/	Baseline	tumor	no	yes
HCC#23	primary	81	m	a.e. MASH	/	/	Baseline	liver	no	yes
HCC#23	primary	81	m	a.e. MASH	/	/	Baseline	margin	yes	no
HCC#23	primary	81	m	a.e. MASH	/	/	Baseline	tumor	no	yes
HCC#24	primary	60	m	ASH	/	/	Baseline	liver	yes	no
HCC#24	primary	60	m	ASH	/	/	Baseline	margin	yes	no
HCC#24	primary	60	m	ASH	/	/	Baseline	tumor	yes	no
HCC#24	recidive	63	m	ASH	/	/	after resection of primary HCC	liver	no	no
HCC#24	recidive	63	m	ASH	/	/	after resection of primary HCC	margin	no	no
HCC#24	recidive	63	m	ASH	/	/	after resection of primary HCC	tumor	no	yes
HCC#25	primary	65	m	MASH	/	HCC/CCC mix	Baseline	liver	no	no
HCC#25	primary	65	m	MASH	/	HCC/CCC mix	Baseline	margin	no	no
HCC#26	primary	74	m	n.d.	/	/	Baseline	liver	yes	yes
HCC#26	primary	74	m	n.d.	/	/	Baseline	margin	yes	no
HCC#26	primary	74	m	n.d.	/	/	Baseline	tumor	yes	yes
HCC#27	primary	62	f	cHCV	/	/	Baseline	liver	no	yes
HCC#27	primary	62	f	cHCV	/	/	Baseline	margin	no	no
HCC#27	primary	62	f	cHCV	/	/	Baseline	tumor	no	yes
HCC#28	primary	81	f	trHCV	/	/	Baseline	liver	no	no
HCC#28	primary	81	f	trHCV	/	/	Baseline	margin	yes	no
HCC#29	primary	60	f	MASH	/	/	Baseline	liver	no	no
HCC#29	primary	60	f	MASH	/	/	Baseline	margin	no	no
HCC#30	primary	54	m	MASH, ASH, trHCV	/	/	Baseline	liver	no	yes
HCC#30	primary	54	m	MASH, ASH, trHCV	/	/	Baseline	margin	yes	no
HCC#30	primary	54	m	MASH, ASH, trHCV	/	/	Baseline	tumor	no	yes
HCC#31	primary	65	m	MASH	rectal cancer FD 2018	/	Baseline	liver	yes	yes
HCC#31	primary	65	m	MASH	rectal cancer FD 2018	/	Baseline	margin	yes	no
HCC#31	primary	65	m	MASH	rectal cancer FD 2018	/	Baseline	tumor	yes	yes
HCC#32	primary	76	m	steatosis hepatitis, a.e. MASH	cured prostate cancer >10 y ago	/	Baseline	liver	no	yes
HCC#32	primary	76	m	steatosis hepatitis, a.e. MASH	cured prostate cancer >10 y ago	/	Baseline	margin	yes	no
HCC#32	primary	76	m	steatosis hepatitis, a.e. MASH	cured prostate cancer >10 y ago	/	Baseline	tumor	no	yes
HCC#33	primary	75	m	steatosis hepatitis, a.e. MASH		/	Baseline	liver	no	yes
HCC#33	primary	75	m	steatosis hepatitis, a.e. MASH		/	Baseline	margin	no	no
HCC#33	primary	75	m	steatosis hepatitis, a.e. MASH		/	Baseline	tumor	no	yes
HCC#34	primary	65	m	n.d.	/	/	Baseline	liver	no	yes
HCC#34	primary	65	m	n.d.	/	/	Baseline	margin	no	no
HCC#34	primary	65	m	n.d.	/	/	Baseline	tumor	no	yes
HCC#35	primary	30	m	cHBV	/	HCC/CCC mix	Baseline	liver	no	yes
HCC#35	primary	30	m	cHBV	/	HCC/CCC mix	Baseline	margin	no	no
HCC#35	primary	30	m	cHBV	/	HCC/CCC mix	Baseline	tumor	no	yes
HCC#36	primary	70	m	ASH	/	/	Baseline	liver	no	yes
HCC#36	primary	70	m	ASH	/	/	Baseline	margin	no	no
HCC#36	primary	70	m	ASH	/	/	Baseline	tumor	no	yes
HCC#36	recidive	72	m	ASH	/	/	after resection of primary HCC	liver	no	no
HCC#36	recidive	72	m	ASH	/	/	after resection of primary HCC	margin	no	no
HCC#37	primary	73	f	none	cured malignant melanoma 2017	/	Baseline	margin	no	no
HCC#37	primary	73	f	none	cured malignant melanoma 2017	/	Baseline	tumor	no	no
HCC#38	primary	64	m	steatosis	/	/	Baseline	liver	no	no
HCC#38	primary	64	m	steatosis	/	/	Baseline	margin	no	no
HCC#39	primary	75	m	MASH	/	/	Baseline	tumor	no	no
HCC#39	recidive	78	m	MASH	/	/	after resection and TACE of primary HCC and first recidive	liver	no	no
HCC#39	recidive	78	m	MASH	/	/	after resection and TACE of primary HCC and first recidive	margin	no	no

REAGENT or RESOURCE	SOURCE	IDENTIFIER
Antibodies		
Primary antibodies:		
4HNE	Abcam	ab48506
AFP	R&D	AF5369
APC anti-mouse IFN-γ Antibody	BioLegend	505810
ATF4	Cell Signalling	11815
ATF6α	Enzo	ADI-905-729-100
ATF6α	OriGene	TA336753
ATF6α n-ATF6α	Signalway Antibody	32008
ATF6α n-ATF6α-Human reactivity	Novus Biologicals	40256
ATF6α-Human reactivity	Abnova	H00022926
B220	BD	553084
BiP	Cell Signaling Technology	3177
CCL2-FITC	ThermoFisher	11-7076-81
CCL5-PE	BioLegend	149103
CD11b-BV650	BioLegend	101239
CD11b-BV711	BioLegend	101242
CD11c-BV421	BioLegend	117343
CD11c-PE.Dazzle	BioLegend	117348
CD19-BV421	BioLegend	115527
CD19-FITC	BioLegend	115505
CD1d-PE	BioLegend	123509
CD200r3-PE	BioLegend	142205
CD206	Proteintech	18704-I-AP
CD206-PE.Dazzle	BioLegend	141731
CD3	Invitrogen	MA1-90582
CD4	eBioscience	14-9766
CD4-AlexaFluor700	BioLegend	100536
CD4-PE	BioLegend	100512
CD44-APC	BioLegend	103012
CD44-PE.Cy7	BioLegend	103029
CD45-BV510	BioLegend	103138
CD62L-PE.Dazzle	BioLegend	104448
CD62L-BV421	BioLegend	104436
CD8	Invitrogen	14-0808-82
CD86-AlexaFluor700	BioLegend	105023
CD8α-PerCP.Cy5	BioLegend	100734
CHOP	Cell Signaling Technology	5554
CHOP/GADD153	Santa Cruz	sc-7351
CK19	Developmental Studies Hybridoma Bank	TROMA-III AB_2133570
clCASP3	Cell Signaling Technology	9661
CLEC4F	R&D	AF2784-SP
Coll-IV	Cedarlane	CL50451AP-1
CyclinD1	Cell Signaling Technology	2978
Eif2α-Mouse	Cell Signaling Technology	9722
F4/80-Mouse	Linaris	T-2006
F480-PE.Cy7	BioLegend	123114
FBP1	Sigma	HPA005857
GAPDH	Cell Signaling Technology	2118
GFP	Novus Biologicals	NB600-308
GP73	Santa Cruz	sc-48011
Granzyme B Monoclonal Antibody (NGZB) - PE	eBioscience	12-8898-82
GS	Abcam	ab16802
HA	Abcam	9110
HIF-1 alpha	Novus Biologicals	NB100-105
IRE1α	Cell Signaling Technology	3294
IFN-γ - APC	BioLegend	505810
KDEL ER marker	Santa Cruz	sc-58774
Ki67	Thermo Scientific	RM-9106-S1
Ki67-BV711	BD	563755
Ly6C- FITC	BioLegend	128006
Ly6G-BV605	BioLegend	127639
MHCII- PerCP.Cy5	BioLegend	107625
Nk1.1- PE.Cy7	BioLegend	108713
NK1.1- BV421	BioLegend	108741
p-Eif2α	Cell Signaling Technology	3868
p-IRE1α	This paper	This paper
p21	Abcam	ab188224
p62	Biozol Diagnostica	MBL-PM045
PARP	Cell Signaling Technology	9532
pcJUN	Cell Signaling Technology	3270
PCNA	Cell Signaling Technology	13110
PD1	R&D	AF1021

PD1-BV605	BioLegend	135220
PD-L1	Cell Signaling Technology	64988
PE/Dazzle™ 594 anti-mouse TNF-α Antibody	BioLegend	506346
SiglecF-APC	BioLegend	155507
TCRβ-BV650	BD	742483
TNFα- PerCP.Cy5	BD	506322
TNF-α -PE/Dazzle	BioLegend	506346
TRAPα	Abcam	ab133238
TXNIP	Cell Signaling Technology	14715
VINCULIN	Sigma	V9131
VINCULIN	Santa Cruz	sc-73614
XBP-1	Santa Cruz	sc-8015
α-PD1	BioCell	BE-0146
α-IgG	BioCell	BE-0090
γ-H2AX	Novus Biologicals	NB100-2280
Secondary antibodies:		
IRDye 680RD Donkey anti-Rabbit IgG	Licor	926-68073
IRDye® 800CW Donkey anti-Rabbit IgG	Licor	926-32213
IRDye 680RD Goat anti-Mouse IgG	Licor	926-68070
IRDye® 800CW Goat anti-Mouse IgG	Licor	926-32210
anti-rabbit-HRP	Cell Signaling Technology	7074
anti-mouse-HRP	Cell Signaling Technology	7076
anti-rat-HRP	Cell Signaling Technology	7077
anti-goat-HRP	Santa Cruz Biotechnology	sc-2354
anti-goat	DAKO	P 0449
anti-mouse	Abcam	ab125904
anti-rat	Jackson Immuno Research	312-005-045
Antibodies *(used in IMC analysis)*		
CD45	Cell Signaling Technology	13917BF
CD68	BioLegend	916104
HLA-DR	abcam	ab176408
SMA	fluidigm	3141017D
CD15	BioLegend	301902
CD3	Cell Signaling Technology	85061BF
CD39	abcam	ab236038
CD163	fluidigm	3147021D
CD11c	abcam	ab52632
CXCR5	Cell Signaling Technology	721725
ATF-6	Abnova	H00022926_M03
TCF1	Cell Signaling Technology	2203BF
TOX	abcam	ab237009
FoxP3	Thermo fisher	14-4777-82
CD4	abcam	ab181724
TCRgd	abcam	ab185753
E-cadherin	Cell Signaling Technology	3195BF
b-Catenin	Cell Signaling Technology	Cat#33893
CD20	BD	555677
CD8a	BioLegend	372902
PD-1	Cell Signaling Technology	86163BF
CD204	invitrogen	14-9054-82
GranzymeB	fluidigm	3167021D
Collagen	fluidigm	3169023D
CD45RO	BioLegend	304202
CD34	abcam	ab198395
HH3	Cell Signaling Technology	4499BF
Opal 520 Reagent Pack	Akoya Biosciences, Inc.	FP1487001KT
Opal 540 Reagent Pack	Akoya Biosciences, Inc.	FP1494001KT
Opal 570 Reagent Pack	Akoya Biosciences, Inc.	FP1488001KT
Opal 620 Reagent Pack	Akoya Biosciences, Inc.	FP1495001KT
Opal 650 Reagent Pack	Akoya Biosciences, Inc.	FP1496001KT
Antibodies (used in animal treatments)		
InVivoMAb rat IgG2a isotype control	Bio X Cell	BXC-BE0089-100MG
InVivoMAb anti-mouse PD-1 (CD279)	Bio X Cell	BXC-BE0146-100MG
Chemicals, peptides, and recombinant proteins		
0.9% NaCl	Growcells	MSDW-0100
10X Spectral DAPI	Akoya Biosciences, Inc.	FP1490
Collagenase IV	Sigma Aldrich	11 088 866 001
Compensation Beads	LIFE Technologies	01-2222-42
Countbright Beads	LIFE Technologies	C36950
Cytokine Cocktail with Brefeldin A	BioLegend	423304
DEN	Sigma Aldrich	N0756

DNAse I	Sigma Aldrich	D5025-150KU
Eosin solution (HE)	Leica Biosystems	3801602E
Erythrolysis reagent	BioLegend	420301
F12K Nut Mix	LIFE Technologies	21127030
Fc-Block CD16/CD32	BioLegend EUROPE B.V	101302
FetalClone II Serum	Cytiva HyClone™	SH3006603
Fix-Perm Buffer	LIFE Technologies	00-5123-43
FoxP3 Fix/Perm Buffer	BioLegend	421403
Galloflavin	BIOTREND CHEMIKALIEN	AOB1024-10
Glucose	Agilent	103577-100
Hematoxylin solution-Mayers (HE)	Leica Biosystems	3801582E
HEPES	Gibco	15630-056
IC Fix Solution	Invitrogen	00-8222-49
Monensin	BioLegend	420701
Non Essential Aminoacids (NEAA)	Gibco	11140-035
Penicillin/Streptomycin	Sigma- Aldrich	P0781-100ML
Percoll	Ge Healthcare	17-0891-01
Perm Buffer	Invitogen	00-8333-56
RPMI 1640	LIFE Technologies	11879020
RPMI 1640+ GlutaMax-I	LIFE Technologies	61870044
Schiff reagent (PAS stainings)	Leica Biosystems	3803800E
Sodium Pyruvate	Gibco	11360-039
SYBR Green (ROX)	Sigma Aldrich	4913914001
Zombie NIR™ Fixable Viability Kit	BioLegend	423106
Western blot reagents		
10X Tris-Buffered Saline	This paper	This paper
10X Tris/Glycine/SDS Migration Buffer	Bio-Rad	1610732
30% Acrylamide solution	Roth- Carl Roth	A124.2
4X Laemmli Buffer	This paper	This paper
Bovine Serum Albumin	Sigma-Aldrich	A2153
Bovine Serum Albumin	Roth	8076.4
Citerion TGX Precast Gels 4-20% 18 well	Bio-Rad	5671094
Citerion TGX Precast Gels 4-20% 26 well	Bio-Rad	5671095
Clarity Western ECL Subs, 500ml	Bio-Rad Laboratories	1705061
Developing machine	Bio-Rad	ChemiDoc™ Touch Gel Imaging System
Halt™ Protease and Phosphatase Inhibitor Cocktail (100X)	Thermo Scientific	78440
Membrane (Nitrocellulose 0.22)	Bio-Rad	1704159
Membrane (PVDF 0.2)	Thermo Scientific	88520
Membrane (PVDF 0.45)	Immobilon	IPVH00010
Odyssey imaging system	Licor	1708370
Phos-STOP Tablets	Roche	4906845001
Protease-STOP	Roche	4693159001
RIPA Buffer	Cell Signaling Technology	9806S
SDS	Sigma-Aldrich	L3771
Skim milk	Serva Electrophoresis	42590.01
Stripping buffer	LIFE Technologies	46430
T-PER	Thermo Scientific	78510
TEMED	CarlRoth	2367.3
Trans-Blot Turbo Transfer system	Bio-Rad	1704150
Transfer Buffer 10X	LIFE Technologies	35040
Tween 20 Detergent	Fisher reagents	BP337-500
Critical commercial assays		
Albumin	FUJIFILM	2050
ALP	FUJIFILM	3550
ALT	FUJIFILM	3250
AST	FUJIFILM	3150
CFX384 Real-time PCR system	Thermo Scientific	QuantStudio 5 Real-Time PCR Systems
Cholesterol	FUJIFILM	1450
Cholesterol Assay	Thermo Scientific	TR13421
Chromium Next GEM Chip G Single Kit	10x Genomics	PN-1000127
Chromium Next GEM Single Cell 3’ GEM, Library and Gel Bead Kit v3.1	10x Genomics	PN - 1000121
Chromium Single Cell 3’ Reagents Kits	10x Genomics	CG000204
E-Plate 96 PET for the xCELLigence	Agilent	300600910
Glucose HK Assay kit	Sigma Aldrich	GAHK20
High-Capacity cDNA Reverse Transcription Kit with RNase Inhibitor	Thermo Scientific	4374967
iTaq Universal SYBR Green Supermix	Bio-Rad	1725124
Lipid extraction kit	BioVision	K216-50
Microvette Z-Gel tubes	Sarstedt	20.1344
Mouse Insulin ELISA	Mercodia	10-1247-01
NEBNext Multiplex Oligos for Illumina (96 Index Primers)	New England Biolabs, Inc.	E6609S
NEBNext Ultra II DNA Library Prep Kit for Illumina	New England Biolabs, Inc.	E7645L
NextSeq 2000 P3 Reagents (50 Cycles) v3	Illumina	20046810
Pierce BCA Protein Assay	Thermo Scientific	23225

Reverse Transcription kit	Sigma Aldrich	4913914001
RNase-free DNAse kit	Qiagen	79254
RNeasy Kit	Qiagen	74106
Seahorse XF glycolysis stress kit	Agilent Technologies	103020-100
Seahorse XFe96 FluxPak mini	Agilent Technologies	102601-100
Serology machine	FUJIFILM	DRI-CHEM NX500i
Triglyceride Assay	Thermo Scientific	TR22421
Triglycerides	FUJIFILM	1650
TURBO-DNA free kit	Thermo Scientific	AM1907
Vetscan Mammalian Liver profile rotor	Abaxis	500-0040-12
Vetscan VS2 Chemistry Analyzer	Abaxis	VS2
Deposited data		
Raw and analyzed RNAseq data	This paper	SuperSeries GSE244344 (GSE244341 = TG-AAV-GFP/CRE/FBP1; GSE244342 = TG-AAV-GFP/CRE DEN/HFD; GSE244343 = Atf6 KO DEN/HFD)；GSE244212 =3M TG$^{Albcre-}$/TG$^{Albcre+}$; GSE244213 =6M TG$^{Albcre-}$/TG$^{Albcre+}$; GSE285265=Atf6$^{\Delta Hep}$, CD-HFD
Raw and analyzed scRNAseq data	This paper	GSE243826; GSE285366
Raw and analyzed aCGH data	This paper	GSE242831
Raw and analyzed proteomic data	This paper	PXD045903
Raw and analyzed CUT&RUN data	This paper	GSE285262
Raw and analyzed ATAC-seq data	This paper	GSE285261
Raw metabolic data	This paper	MTBLS13241
Experimental models: Cell lines		
Colo800	Kind gift from Dr. Rafael Carretero	This paper
FL83B	ATCC	CRL-2390
HLE	originated from JCRB	This paper
Oligonucleotides (Mouse)		
Gene	*Sequence*	
Acta2 (FW)	TCACCATTGGAAACGAACGC	
Acta2 (RV)	GCTGTTATAGGTGGTTTCGTGG	
Actin-b (FW)	GTA CTC TGT GTG GAT CGG TGG	
Actin-b (RV)	AAC GCA GCT CAG TAA CAG TCC	
Afp (FW)	GGA CTG CTC GAA ACA TCC CA	
Afp (RV)	CTT TGG ACG CAG CGA AAT GT	
AldoA (FW)	CACGAGACACTGTACCAGAAGG	
AldoA (RV)	TTGTCTCGCCATTGGTTCCTGC	
Atf4 (FW)	ATGGCCGGCTATGGATGAT	
Atf4 (RV)	CGAAGTCAAACTCTTTCAGATCCATT	
Atf6 (FW)	CTTCCTCCAGTTGCTCCATC	
Atf6 (RV)	CAACTCCTCAGGAACGTGCT	
Ccl2 (FW)	GCATCCACGTGTTGGCTCA	
Ccl2 (RV)	CTCCAGCCTACTCATTGGGATCA	
Cdh1 (FW)	CATGTTCACTGTCAATAGGG	
Cdh1 (RV)	GTGTATGTAGGGTAACTCTCTC	
Creld2 (FW)	CAACACGGCCAGGAAGAATTT	
Creld2 (RV)	CATGATCTCCAGAAGCCGGAT	
Cxcl10 (FW)	CGATGACGGGCCAGTGAGAATG	
Cxcl10 (RV)	TCAACACGTGGGCAGGATAGGCT	
Ddit3 (FW)	CTGCCTTTCACCTTGGAGAC	
Ddit3 (RV)	CGTTTCCTGGGGATGAGATA	
Dnajb9 (FW)	CGGGGCGCACAGGTTATTAG	
Dnajb9 (RV)	AGCTTTTGGAGGCCAGGATT	
Dnajc3 (FW)	GGCGCTGAGTGTGGAGTAAAT	
Dnajc3 (RV)	GCGTGAAACTGTGATAAGGCG	
Eno1 (FW)	TACCGCCACATTGCTGACTTGG	
Eno1 (RV)	GCTTGTTGCCAGCATGAGAACC	
Eno2 (FW)	TGGCAAGGATGCCACTAACGTG	
Eno2 (RV)	AACTCAGAGGCAGCCACATCCA	
Ero1l (FW)	TGTGGACTTACTCCTTAACC	
Ero1l (RV)	TTGTCTGTGGCTTAAAACAG	
Fbp1 (FW)	AAGTACTGATGAGCCTTCTG	
Fbp1 (RV)	GTCCACCATAATGAATTCTCC	
Fos (FW)	GAAGGGAACGGAATAAGATG	
Fos (RV)	CATCTTCAAGTTGATCTGTCTC	
Gfpt1 (FW)	AATGCTGGTCCTGAGATTGGCG	
Gfpt1 (RV)	CCGAGCATGATCTCTTTGCGTC	
Gpc3 (FW)	CGGTGATGAAGATGAATG	
Gpc3 (RV)	CTCGTTGTCCTTCTGATTTC	
Gpi1 (FW)	CCATCAAGGTGGACGGCAAAGA	
Gpi1(RV)	CCGTGATGGATTTGCCAGTGTAC	

Gys1 (FW)	CACAGAACGGTTGTCGGACTTG	
Gys1 (RV)	AGGTGAAGTGGTCTGGAAAGGC	
Herpud1 (FW)	GATGGGAGAACATCTCTAGG	
Herpud1 (RV)	GCAGCTAAGTATTGCATGTAG	
Hsp90b1 (FW)	CGAGGTCTGTTTGATGAATATG	
Hsp90b1 (RV)	GAATCCACAACACCTTTGAC	
Hspa5 (FW)	ACTTGGGGACCACCTATTCCT	
Hspa5 (RV)	ATCGCCAATCAGACGCTCC	
Hyou (FW)	GAAGATTTTGCTGAACAACC	
Hyou (RV)	GGAATTGATATCTTTCCGGC	
Il23 (FW)	CTGTGCCTAGGAGTAGCAG	
Il23 (RV)	TAGTAGATTCATATGTCCCGCTG	
Myc (FW)	TTTTGTCTATTTGGGGACAG	
Myc (RV)	CATAGTTCCTGTTGGTGAAG	
Pdia4 (FW)	CTTTGATTACAGAGCTGCTAC	
Pdia4 (RV)	AAGGTATACTCAGGGAAGTC	
Pdia6 (FW)	ACAGAAGTAAAGGAGCAAAC	
Pdia6 (RV)	GAATCCTTTAATCCCGTATCG	
Pfkfb4 (FW)	GAGCCAGATGAAGAGGACGATC	
Pfkfb4 (RV)	GCAAACTCCAGCGGGTAGTGAT	
Pfkl (FW)	CCGCACCTTGAGCATAGACA	
Pfkl (RV)	CGAGTTCCCCTTGAGTTCCC	
Pgk1 (FW)	GATGCTTTCCGAGCCTCACTGT	
Pgk1 (RV)	ACCAGCCTTCTGTGGCAGATTC	
Pkm (FW)	CAGAGAAGGTCTTCCTGGCTCA	
Pkm (RV)	GCCACATCACTGCCTTCAGCAC	
Sel1l (FW)	CTCTTGTTTGGTGATTACCTC	
Sel1l (RV)	AATATACAAGAGCCTTTGCC	
Slc2α1 (FW)	ACCTCTTCCGAACCGACAGAT	
Slc2α1 (RV)	TCTGGAGCCATCAAAGTCCTG	
Slc2α10 (FW)	GGACCAGTTCTGAACACTGCCT	
Slc2α10 (RV)	TCTCACTGAGGACCAACCAGGT	
Slc2α6 (FW)	GGCTCCTATCTGTGCTGATTGC	
Slc2α6 (RV)	CCTTGGCACAAACTGGACGTAG	
Slc2α8 (FW)	CCTTCGTGACTGGCTTTGCTGT	
Slc2α8 (RV)	TGGGTAGGCGATTTCCGAGATG	
Tnf (FW)	CCCTCACACTCAGATCATCTTCT	
Tnf (RV)	GCTACGACGTGGGCTACAG	
uPA (FW)	GCGCCTTGGTGGTGAAAAAC	
uPA (RV)	GACACGCATACACCTCCGTT	
Xbp1s (FW)	GAGTCCGCAGCAGGTG	
Xbp1s (RV)	GTGTCAGAGTCCATGGGA	
Xbp1u (FW)	AAGAACACGCTTGGGAATGG	
Xbp1u (RV)	ACT CCC CTT GGC CTC CAC	
GalNac-ASO-Scramble	Gen 2.5 ASO (16mer 3-10-3);<br>CGCCGATAAGGTACAC (GalNac on 5')	This paper (Ionis Pharmaceuticals)
GalNac-ASO-Atf6	Gen 2.5 ASO (16mer 3-10-3);<br>GAATTTTTCAGCAAGG (GalNac on 5')	This paper (Ionis Pharmaceuticals)
Recombinant DNA		
AAV8-TBG-iCRE	Vector Biolabs	VB1724
AAV8-Fbp1 or AAV8-Fbp1E98A	Kind gift from Dr. M. Karin, amplified for use *in vivo* by SBP Viral Vector Core	This paper
AAV8-TBG-eGFP	Vector Biolabs	VB1743
pCMV-SB13	Kind gift from D.Tschaharganeh, DKFZ, Heidelberg	This paper
pCMV(CAT)T7-SB100	Addgene	34879
pT/Caggs-NRASV12	Addgene	20205
pT3-EF1α-KRASG12D-EGFP	Kind gift from D.Tschaharganeh, DKFZ, Heidelberg	This paper
pT3-EF1α-MYC	Kind gift from D.Tschaharganeh, DKFZ, Heidelberg	This paper
px330-sgTp53	Addgene	59910
Software and algorithms *(see Methods for additional software, code source, and associated details)*		
Agilent Wave (Seahorse Bioanalyzer)	https://www.agilent.com/en/products/cell-analysis/software-download-for-wave-	
FlowJo	https://www.flowjo.com/solutions/flowjo/ downloads	
GSEA/MSigDB	https://www.gsea-msigdb.org/gsea/index.jsp	
GraphPad Prism	https://www.graphpad.com/scientific-software/prism/	
ImageJ	https://imagej.nih.gov/ij/download.html	
Qiagen IPA	https://analysis.ingenuity.com/pa/installer/select	
QuPath	https://qupath.github.io	
