# Supplementary material for: Activated ATF6α is a hepatic tumour driver restricting immunosurveillance
Source: Nature. 2026 Feb 4;651(8106):796–807. doi: 10.1038/s41586-025-10036-8 (PMC12999494; doi:10.1038/s41586-025-10036-8)
Supplement: Supplementary file 2 — Reporting Summary [file 41586_2025_10036_MOESM2_ESM.pdf]

nature portfolio

Corresponding author(s): Prof. Dr. Mathias Heikenwälder

Last updated by author(s): Nov 26, 2025

Reporting Summary

Nature Portfolio wishes to improve the reproducibility of the work that we publish. This form provides structure for consistency and transparency in reporting. For further information on Nature Portfolio policies, see our Editorial Policies and the Editorial Policy Checklist.

Statistics

For all statistical analyses, confirm that the following items are present in the figure legend, table legend, main text, or Methods section.

n/a	Confirmed	
☐	☒	The exact sample size (*n*) for each experimental group/condition, given as a discrete number and unit of measurement
☐	☒	A statement on whether measurements were taken from distinct samples or whether the same sample was measured repeatedly
☐	☒	The statistical test(s) used AND whether they are one- or two-sided<br>*Only common tests should be described solely by name; describe more complex techniques in the Methods section.*
☐	☒	A description of all covariates tested
☐	☒	A description of any assumptions or corrections, such as tests of normality and adjustment for multiple comparisons
☐	☒	A full description of the statistical parameters including central tendency (e.g. means) or other basic estimates (e.g. regression coefficient) AND variation (e.g. standard deviation) or associated estimates of uncertainty (e.g. confidence intervals)
☐	☒	For null hypothesis testing, the test statistic (e.g. *F*, *t*, *r*) with confidence intervals, effect sizes, degrees of freedom and *P* value noted<br>*Give P values as exact values whenever suitable.*
☒	☐	For Bayesian analysis, information on the choice of priors and Markov chain Monte Carlo settings
☒	☐	For hierarchical and complex designs, identification of the appropriate level for tests and full reporting of outcomes
☐	☒	Estimates of effect sizes (e.g. Cohen's *d*, Pearson's *r*), indicating how they were calculated

Our web collection on statistics for biologists contains articles on many of the points above.

Software and code

Policy information about availability of computer code

Data collection	Data were collected with Microsoft Excel, Office 2019 (v16).
Data analysis	All relevant data analysis have been described in detail in the Methods section of the manuscript.<br><br>For softwares:<br><br>GraphPad Prism software (version 9.3.1 or 10.0.3) was used for statistical analysis (generally presented as mean ±SEM). Sample sizes and statistical tests used are indicated in the legends. Exact p-values over p<0.0001 are reported.<br><br>FlowJo (version 10) was used to analyze FACS data obatined from BD FACSFortessa machine.<br><br>Data from Metabolic Flux Analysis (Seahorse bioanalyzer) were calculated with Agilent Wave Software V2.6.<br><br>Histological staining and in-situ hybridization slides were analyzed by macro-based analysis by ImageJ (1.54g) or QuPath (v0.5.1).<br><br>10x Genomics cellranger-count pipeline (v5.0.1), FastQC (version v0.11.8), MultiQC (v1.7), the Scanpy package (v1.9.2), the Seurat R package (v2.4.3), the monocle package (v2.24.0) and the GSVA package (v1.48.3) was utilized to proceed and analyze the mouse 10x Genomics single-cell RNA sequencing data.<br><br>For Western blot analysis, quantification of bands of interest in the linear range of exposure was performed by densitometry using imageJ (1.53k).

The Maxquant9 (V2.4.3) and Persues R package were used to analyze the proteomics data.

Cutadapt v2.3, STAR aligner v2.7.0d_022111, RSEM v1.3.1, Ensembl v84 gene annotations, FastQC v0.11.5, MultiQC v1.8, the R Bioconductor package DESeq2 v1.22.2, the Ingenuity Pathway Analysis (Qiagen, Redwood City, USA) were used to proceed and analyze bulk RNA-seq data at the SBP Genomics Core.

FastQC (v0.11.9), Trim Galore (v0.6.5), STAR alinger (V2.7.10), the subread R package (v1.6.4), dupRader, RSeQC(V2.6.4), the DEseq2 R package (V1.40.2), the dist R (V3.8), and the gProfiler2 R (V0.2.0) package were used to proceed and analyze bulk RNA-seq data at the Genomics & Proteomics Core Facility in DKFZ.

The Catalyst R package, the Cellprofiler Cellpose 2.0 plugin and OMIQ (https://www.omiq.ai/) platform were used to analyze data obtained from imaging mass cytometry.

NMR-based metabolimic data were proceed and analyzed with Bruker TopSpin 3.6.1 software, ChenomX NMR suite 8.5 software and the MetaboAnalyst 5.0 online platform (www.metaboanalyst.ca).

LC-MS metabolic data analysis was performed using MS-Dial 4.9.2, enabling compound identification based on accurate mass and MS2 spectra, supported by an in-house retention time library.

The “SCAN.UPC” R/Bioconductor package, the “GSVA” (Gene Set Variation Analysis) Bioconductor package, the “metafor” R package, and the “sva” R package were used for analyzing the human ATF6α-activation signature in different databases. The Kaplan-Meier survival analysis was performed in the Python using the lifelines package and visualized using matplotlib.

CUT&RUN data were analyzed using the nf-core/cutandrun pipeline v3.2.2 with Nextflow version 24.04.2, keeping default parameters and following software dependencies: bedtools (v2.30.0), bowtie (v2.4.4), deeptools (v3.5.1), fastqc (v0.12.1), picard (v3.1.0), python (v3.9.12), samtools (v1.17), Genrich (v0.6.1), TrimGalore (v0.6.6), ucsc (v377). CUT&RUN analysis identified direct target genes by using the HOMER’s annotatePeaks.pl tool.

ATAC-Seq data were analyzed using the nf-core/atacseq pipeline v2.1.2 with Nextflow version 24.0.2, using default parameters.

Pipelines and code used in data analysis are listed below:

Multiplexed ion beam imaging (MIBI): Low level processing is available at https://github.com/a-ngelolab/toffy and cell segmentation pipeline available at https://github.com/mouseland/cellpose.

Bulk RNA-seq: ENCODE long RNA-seq pipeline (https://github.com/ENCODE-DCC/long-rna-seq-pipeline) and Trim Galore (v0.6.5) (https://github.com/FelixKrueger/TrimGalore).

IMC: Correction for signal spillover of the metal isotopes were compensated using the Catalyst package in R with the script available at https://github.com/NiklasVesper/ImagingCytometryTools. The full CellProfiler pipeline for image segmentation with detailed settings and structure can be found upon request under: https://github.com/NiklasVesper/ImagingCytometryTools. All scripts for Neighborhood analysis are available upon request at: https://github.com/NiklasVesper/ImagingCytometryTools.

For manuscripts utilizing custom algorithms or software that are central to the research but not yet described in published literature, software must be made available to editors and reviewers. We strongly encourage code deposition in a community repository (e.g. GitHub). See the Nature Portfolio guidelines for submitting code & software for further information.

Data

Policy information about availability of data

All manuscripts must include a data availability statement. This statement should provide the following information, where applicable:

- Accession codes, unique identifiers, or web links for publicly available datasets
- A description of any restrictions on data availability
- For clinical datasets or third party data, please ensure that the statement adheres to our policy

For data-sets generated in this study:
The proteomics data are available at ProteomeXchange Consortium via the PRIDE database, under the Project accession: PXD045903. The bulk-RNA-seq data are available at SuperSeries GSE244344 (GSE244341 = TGAAV-gfp, TGAAV-cre or TGAAV-cre/fbp1; GSE244342 = TGAAV-gfp or TGAAV-cre + DEN/HFD; GSE244343 = Atf6+/+ or Atf6-/- DEN/HFD), and GSE244212 (3M TGAlbcre- or TGAlbcre+), GSE244213 (6M TGAlbcre- or TGAlbcre+), GSE285265 (Atf6fl/fl or Atf6ΔHep mice + CD-HFD). Raw reads were aligned to the reference mouse genome: Mus musculus (house mouse) genome assembly GRCm38 (mm10) from Genome Reference Consortium [GCA_000001635 2 GCF_000001635.20). The single-cell RNA-seq data are available at GEO Submission (GSE243826) and GEO Submission (GSE285366).The single-cell RNA-seq data are available at GEO Submission (GSE243826) and GEO Submission (GSE285366). The CUT&RUN data are available at GEO Submission (GSE285262). The ATAC-seq data are available at GEO Submission (GSE285261). The array of comparative genomic hybridization data is available at GEO Submission (GSE242831). The NMR-based metabolomics data and LC-MS/MS metabolic analysis are available at the Metabolights database, via the study MTBLS13241.

For previously published datasets:
The human ATF6α-activation signature was derived from the Molecular Signatures Database (MSigDB, www.msigdb.org) Human Gene Set: ATF6_TARGET_GENES. 22 datasets with extensive whole-genome RNA expression data were chosen (TCGA-LIHC, GSE77314, LIRI-JP, GSE56545, GSE77509, GSE54236, GSE17856, GSE47197, GSE14520, GSE45436, GSE63898, GSE84005, GSE64041, GSE76297, GSE25097, GSE45114, GSE76427, GSE36376, GSE39791, GSE36411, GSE57957, GSE84598) to evaluate the correlation between the expression levels of genes and gene signatures in human HCC. The results here are in part based upon data generated by the TCGA Research Network: https://www.cancer.gov/tcga. We also used data-sets from the following publications: Villanueva et al., 2015 (https://doi.org/10.1002/hep.27732); Montironi et al., 2023 (https://doi.org:10.1136/gutjnl-2021-325918), Haber et al., 2023 (https://doi.org/10.1053/

j.gastro.2022.09.005), and Jiang et al., 2019 (https://doi.org/10.1038/s41586-019-0987-8).

Research involving human participants, their data, or biological material

Policy information about studies with human participants or human data. See also policy information about sex, gender (identity/presentation), and sexual orientation and race, ethnicity and racism.

Reporting on sex and gender	The respective sex and gender information are reported in Supplementary Information Table 2.
Reporting on race, ethnicity, or other socially relevant groupings	No further information about race, ethnicity, or other socially relevant groupings are reported.
Population characteristics	Human characteristics are reported in Supplementary Information Table 2.
Recruitment	No active recruitment of human participants was done in the frame of this study. The clinical samples and data we used—including tissue microarrays from HCC patients and their control group, as well as liver tissue sections and frozen samples from patients with liver disease or HCC—were all obtained from existing patient samples provided by collaborating hospitals or related institutions, in strict accordance with their relevant ethical regulations. The authors and collaborators of this study conducted research and analyses on the acquired samples and data, all accessible samples were analyzed, and there was no self-selection. The transcriptomic and proteomic data used in this study for liver cancer patients were all from previously published datasets.
Ethics oversight	The respective ethics committee is listed in the Methods section.<br><br>Human HCC tissue microarrays (TMAs) used in this study were obtained with informed patient consent from Prof. Kai Breuhahn as described by him previously (https://doi.org:10.1186/s12964-023-01169-2). The use of these TMAs in current study is approved by the institutional ethics committee of the Medical Faculty of Heidelberg University (S-206/2005).<br><br>Liver sections and snap-frozen tissue samples from healthy donors and patients with hepatitis were obtained from Dr. Mohammad Rahbari and Dr. Nuh Rahbari with the approved institutional review board (IRB) protocol (2012-293N-MA) from the University Hospital Mannheim; from Dr. Susanne Roth in Department of Surgery at Heidelberg University Hospital with the approved ethical protocol S-629/2013; from Dr. Achim Weber with the approved application number KEK-ZH-Nr. 2013-0382 by the local ethics committee (Kantonale Ethikkommission Zurich) in University Hospital Zurich.<br><br>Human liver sections involved in Spatial Biology and Imaging Mass Cytometry analysis were obtained from Dr. Maike Hofmann. Written informed consent was obtained in all cases and the study was conducted according to the Declaration of Helsinki (1975), federal guidelines and local ethics committee regulations (Albert-Ludwigs-University, Freiburg, Germany, approvals 20-1066).

Note that full information on the approval of the study protocol must also be provided in the manuscript.

Field-specific reporting

Please select the one below that is the best fit for your research. If you are not sure, read the appropriate sections before making your selection.

☒ Life sciences ☐ Behavioural & social sciences ☐ Ecological, evolutionary & environmental sciences

For a reference copy of the document with all sections, see nature.com/documents/nr-reporting-summary-flat.pdf

Life sciences study design

All studies must disclose on these points even when the disclosure is negative.

Sample size	Plot experiments and previously published results (e g. Pfister et al, Nature 2021; Dudek et al, Nature 2021) were used to estimate the sample size, such that appropriate statistical tests could yield significant results. No further statistical methods were used to predetermine sample size. The sample size and exact 'n' numbers are indicated in the Methods and Figure Legends.
Data exclusions	No data exclusion of human data. Mice with obvious cholemic phenotype were excluded from dietary experiment.
Replication	All experiments presented were conducted with sufficient mouse numbers to ensure statistical significance could be reached, particularly for experiments involving tumor studies. Biochemical or image based data were reproduced in multiple mice: e.g., Weight analysis of mice measuring transaminase levels, liver tumor development characterization, flow cytometry analyses, immunohistochemical staining, confocal analysis. Mouse in vivo and in vitro experiments were repeated at least three times. All attempts of replicating data were successful.
Randomization	Mice were randomly allocated into different groups to make sure the phenotype was homogeneous across groups, and were then fed with appropriate diet and/or administered their respective treatment regimens. Randomization of human patients was not applicable as no prospective trial/study was performed (no active recruitment of human patients, as indicated above) and human samples evaluated were obtained from pre-existing human patient cohorts/databases, adhering to ethical guidelines.
Blinding	Investigators were blinded to group allocation for all experiments in which blinding was technically feasible. Experiments of mice with

Blinding

different genotypes on the same diet were blinded. Quantitative analyses, including serum transaminase measurements, IC/IF quantification, immunoblot densitometry, and flow cytometry (FACS) analysis, were conducted using sample code labeling. Blinding was not possible for studies comparing preclinical liver cancer mouse models in which features were visually distinguishable (e.g., normal chow pellets were brown, high-fat diet pellets were blue; obese vs. lean phenotypes; liver tumors inherently visible during dissection). Human patient data underwent pseudonymisation and was blinded to the analyzer.

Reporting for specific materials, systems and methods

We require information from authors about some types of materials, experimental systems and methods used in many studies. Here, indicate whether each material, system or method listed is relevant to your study. If you are not sure if a list item applies to your research, read the appropriate section before selecting a response.

Materials & experimental systems

n/a	Involved in the study
☐	☒ Antibodies
☐	☒ Eukaryotic cell lines
☒	☐ Palaeontology and archaeology
☐	☒ Animals and other organisms
☐	☒ Clinical data
☒	☐ Dual use research of concern
☒	☐ Plants

Methods

n/a	Involved in the study
☒	☐ ChIP-seq
☐	☒ Flow cytometry
☒	☐ MRI-based neuroimaging

Antibodies

Antibodies used

Antibodies
Primary antibodies: (Antibody dilutions followed manufacturer's guidelines)
4HNE Abcam ab48506
AFP R&D AF5369
APC anti-mouse IFN-γ Antibody BioLegend 505810
ATF4 Cell Signalling 11815
ATF6α Enzo ADI-905-729-100
ATF6α OriGene TA336753
ATF6α n-ATF6α Signalway Antibody 32008
ATF6α n-ATF6α-Human reactivity Novus Biologicals 40256
ATF6α-Human reactivity Abnova H00022926
B220 BD 553084
BiP Cell Signaling Technology 3177
CCL2-FITC ThermoFisher 11-7076-81
CCL5-PE BioLegend 149103
CD11b-BV650 BioLegend 101239
CD11b-BV711 BioLegend 101242
CD11c-BV421 BioLegend 117343
CD11c-PE.Dazzle BioLegend 117348
CD19-BV421 BioLegend 115527
CD19-FITC BioLegend 115505
CD1d-PE BioLegend 123509
CD200r3-PE BioLegend 142205
CD206 Proteintech 18704-l-AP
CD206-PE.Dazzle BioLegend 141731
CD3 Invitrogen MA1-90582
CD4 eBioscience 14-9766
CD4-AlexaFluor700 BioLegend 100536
CD4-PE BioLegend 100512
CD44-APC BioLegend 103012
CD44-PE.Cy7 BioLegend 103029
CD45-BV510 BioLegend 103138
CD62L-PE.Dazzle BioLegend 104448
CD62L-BV421 BioLegend 104436
CD8 Invitrogen 14-0808-82
CD86-AlexaFluor700 BioLegend 105023
CD8α-PerCP.Cy5 BioLegend 100734
CHOP Cell Signaling Technology 5554
CHOP/GADD153 Santa Cruz sc-7351
CK19 Developmental Studies Hybridoma Bank TROMA-III AB_2133570
clCASP3 Cell Signaling Technology 9661
CLEC4F R&D AF2784-SP
Coll-IV Cedarlane CL50451AP-1
CyclinD1 Cell Signaling Technology 2978
Eif2α-Mouse Cell Signaling Technology 9722

F4/80-Mouse Linaris T-2006
F480-PE.Cy7 BioLegend 123114
FBP1 Sigma HPA005857
GAPDH Cell Signaling Technology 2118
GFP Novus Biologicals NB600-308
GP73 Santa Cruz sc-48011
Granzyme B Monoclonal Antibody (NGZB) - PE eBioscience 12-8898-82
GS Abcam ab16802
HA Abcam 9110
HIF-1 alpha Novus Biologicals NB100-105
IRE1α Cell Signaling Technology 3294
IFN-γ - APC BioLegend 505810
KDEL ER marker Santa Cruz sc-58774
Ki67 Thermo Scientific RM-9106-S1
Ki67-BV711 BD 563755
Ly6C- FITC BioLegend 128006
Ly6G-BV605 BioLegend 127639
MHCII- PerCP.Cy5 BioLegend 107625
Nk1.1- PE.Cy7 BioLegend 108713
NK1.1- BV421 BioLegend 108741
p-Eif2α Cell Signaling Technology 3868
p-IRE1α This paper This paper
p21 Abcam ab188224
p62 Biozol Diagnostica MBL-PM045
PARP Cell Signaling Technology 9532
pcJUN Cell Signaling Technology 3270
PCNA Cell Signaling Technology 13110
PD1 R&D AF1021
PD1-BV605 BioLegend 135220
PD-L1 Cell Signaling Technology 64988
PE/Dazzle™ 594 anti-mouse TNF-α Antibody BioLegend 506346
SiglecF-APC BioLegend 155507
TCRβ-BV650 BD 742483
TNFα- PerCP.Cy5 BD 506322
TNF-α -PE/Dazzle BioLegend 506346
TRAPα Abcam ab133238
TXNIP Cell Signaling Technology 14715
VINCULIN Sigma V9131
VINCULIN Santa Cruz sc-73614
XBP-1 Santa Cruz sc-8015
α-PD1 BioCell BE-0146
α-IgG BioCell BE-0090
γ-H2AX Novus Biologicals NB100-2280

Secondary antibodies:
IRDye 680RD Donkey anti-Rabbit IgG Licor 926-68073
IRDye® 800CW Donkey anti-Rabbit IgG Licor 926-32213
IRDye 680RD Goat anti-Mouse IgG Licor 926-68070
IRDye® 800CW Goat anti-Mouse IgG Licor 926-32210
anti-rabbit-HRP Cell Signaling Technology 7074
anti-mouse-HRP Cell Signaling Technology 7076
anti-rat-HRP Cell Signaling Technology 7077
anti-goat-HRP Santa Cruz Biotechnology sc-2354
anti-goat DAKO P 0449
anti-mouse Abcam ab125904
anti-rat Jackson Immuno Research 312-005-045

Antibodies (used in IMC analysis)
CD45 Cell Signaling Technology 13917BF
CD68 BioLegend 916104
HLA-DR abcam ab176408
SMA fluidigm 3141017D
CD15 BioLegend 301902
CD3 Cell Signaling Technology 85061BF
CD39 abcam ab236038
CD163 fluidigm 3147021D
CD11c abcam ab52632
CXCR5 Cell Signaling Technology 721725
ATF-6 Abnova H00022926_M03
TCF1 Cell Signaling Technology 2203BF
TOX abcam ab237009
FoxP3 Thermo fisher 14-4777-82
CD4 abcam ab181724
TCRgd abcam ab185753
E-cadherin Cell Signaling Technology 3195BF
b-Catenin Cell Signaling Technology Cat#33893

	CD20 BD 555677<br>CD8a BioLegend 372902<br>PD-1 Cell Signaling Technology 86163BF<br>CD204 invitrogen 14-9054-82<br>GranzymeB fluidigm 3167021D<br>Collagen fluidigm 3169023D<br>CD45RO BioLegend 304202<br>CD34 abcam ab198395<br>HH3 Cell Signaling Technology 4499BF<br><br>Opal 520 Reagent Pack Akoya Biosciences, Inc. FP1487001KT<br>Opal 540 Reagent Pack Akoya Biosciences, Inc. FP1494001KT<br>Opal 570 Reagent Pack Akoya Biosciences, Inc. FP1488001KT<br>Opal 620 Reagent Pack Akoya Biosciences, Inc. FP1495001KT<br>Opal 650 Reagent Pack Akoya Biosciences, Inc. FP1496001KT<br><br>Antibodies (used in animal treatments)<br>InVivoMAb rat IgG2a isotype control Bio X Cell BXC-BE0089-100MG<br>InVivoMAb anti-mouse PD-1 (CD279) Bio X Cell BXC-BE0146-100MG
Validation	Validation of commercial antibodies was done on a regular quality control of each lot by the manufacturer (e.g. Biolegend "The antibody was purified by affinity chromatography and conjugated with PE under optimal conditions"; "Each lot of this antibody is quality control tested by immunofluorescent staining with flow cytometric analysis."; "Every lot of product is quality tested against a "gold standard" reference lot. A new lot is only released based on our defined QC specifications to ensure lot to lot reproducibility and reliability. Biolegend guarantees the stability and performance of all our products shipped at room temperature. Wherever possible, knock-out validated antibodies were used. At CST, "we validate all of the antibodies we sell in-house using rigorous, application-specific testing". At Abcam "Biophysical QC enables confirmation of antibody identity at a molecular level, delivering robust, reproducible results for the best lot-to-lot consistency". "We can use this data to validate subsequent batches guaranteeing the highest specificity, sensitivity and consistency every time".

Eukaryotic cell lines

Policy information about cell lines and Sex and Gender in Research

Cell line source(s)	Col0800: Kind gift from Dr. Rafael Carretero<br>FL83B: ATCC CRL-2390<br>HLE: Riken BRC Cell Bank
Authentication	FL83B were authenticated with purchase from ATCC (STR profiling for the cell line verifies its authenticity). HLE and Colos00 cells were not authenticated beyond in-house cell morphology validation.
Mycoplasma contamination	All cell lines are tested negative for mycoplasma contamination by ATCC and by Microbiological diagnostics DKFZ.
Commonly misidentified lines<br>(See ICLAC register)	No commonly misidentified cell lines were used in this study.

Animals and other research organisms

Policy information about studies involving animals; ARRIVE guidelines recommended for reporting animal research, and Sex and Gender in Research

Laboratory animals	A animals in this study were described in Methods and Supplementary information. All mouse lines were either on a pure C57BL/6J background or crossed into in for at least 10 generations. Experimental schemes with timelines for treatment regimens are shown in corresponding Figures. Specifically, where indicated, DEN injections were done at 2 weeks old, special diets were started after 6 weeks old, AAV/oncogene injections were done between 6-10 weeks old, ASO injections were started at 4, 9 or 30 weeks old. Anti-IgG/PD1 treatment were done on 9-month-old Transgenic mice.
Wild animals	not used
Reporting on sex	All mice used in this study were age, gender, and genetic background matched. Detailed sex information of experimental animals were provided in respective figure legend or methods.
Field-collected samples	not used
Ethics oversight	Regierungspräsidium Karlsruhe, Karlsruhe, Germany, or National Institute of Health (NIH) guidelines of the United States, with approval from the Sanford Burnham Prebys Institutional Animal Care and Use Committee (IACUC), San Diego, CA, USA.

Note that full information on the approval of the study protocol must also be provided in the manuscript.

Clinical data

Policy information about clinical studies

All manuscripts should comply with the ICMJE guidelines for publication of clinical research and a completed CONSORT checklist must be included with all submissions.

Clinical trial registration	This study was not performed in active clinical trials.
Study protocol	This study was not performed in active clinical trials.
Data collection	The respective data collection (fresh/retrospective cohort analysis/analyses of published data) process is listed in the Methods section.
Outcomes	This study was not performed in active clinical trials.

Plants

Seed stocks	No plant material used in this study.
Novel plant genotypes	No plant material used in this study.
Authentication	No plant material used in this study.

Flow Cytometry

Plots

Confirm that:

☒ The axis labels state the marker and fluorochrome used (e.g. CD4-FITC).

☒ The axis scales are clearly visible. Include numbers along axes only for bottom left plot of group (a 'group' is an analysis of identical markers).

☒ All plots are contour plots with outliers or pseudocolor plots.

☒ A numerical value for number of cells or percentage (with statistics) is provided.

Methodology

Sample preparation	Please see Methods and Supplementary Information.<br>The isolation and staining of lymphocytes for flow cytometry followed the protocol described in Methods. Animals were sacrificed and livers perfused with 0.9% NaCl buffer. Livers were collected, minced, digested with Collagenase and DNase, and subsequently passed through a 100µm filter. Hepatic lymphocytes were then purified by a 2-step Percoll gradient. Spleens were passed through 100µm mesh and washed to isolate splenic lymphocytes. The samples were treated with red blood cell lysis buffer for 5 minutes at RT, followed by a washing step.<br>For lymphocyte stimulation, cells were cultured in RPMI 1640 supplemented with 2%(v/v) FBS. Cell Activation Cocktail with Brefeldin A (Biolegend #423304) and Monensin Solution (Biolegend #420701) were diluted in the medium at the rate of 1:500 and 1:1000, respectively. Antibody staining was done in the presence of Fc receptor blockade in flow cytometry-activated cell sorting (FACS) buffer. For live/dead cell discrimination, the ZombieDyeNIR dye was used according to the manufacturer's guidelines. After washing with FACS buffer and centrifugation (400g, 5min, 4°C), cells were stained for 40min at 4°C with 25µl of titrated antibody master mix and then washed. For sorting experiments, the samples were then sorted using FACS. eBioscience IC fixation (#00-8222-49) was used to fix samples for samples requiring only surface staining for flow cytometry, as directed by the manufacturer's guidelines. For samples requiring intracellular staining, eBioscience Perm buffer (#00-8333-56) was used. BD FACSFortessa was used to analyze the stained cells, and FlowJo was used to analyze data. In collaboration with the DKFZ FACS core facility, a FACS Aria II machine and a FACS Aria FUSION machine were employed for sorting.
Instrument	Cells were analyzed using BD FACSFortessa or BD FACSSymphony. For sorting, a FACS Aria II and a FACSAria FUSION in collaboration with the DKFZ FACS core facility were used.
Software	Collected data was analyzed by FlowJo V10.2.
Cell population abundance	Absolute quantification by using CountBright™ Absolute Counting Beads.
Gating strategy	Gating strategy were provided in Supplementary Figure.

Gating strategy

Debry esclusion byFSC-A/SSC-A. Doublets were excluded by using FSC-A/FSC-H and SSC-A/SSC-H gates. Life/Dead exclusion was performed. Remaining cells were analyzed according to displayed markers.

☒ Tick this box to confirm that a figure exemplifying the gating strategy is provided in the Supplementary Information.
